# Active mode of excretion across digestive tissues predates the origin of excretory organs

**DOI:** 10.1371/journal.pbio.3000408

**Published:** 2019-07-29

**Authors:** Carmen Andrikou, Daniel Thiel, Juan A. Ruiz-Santiesteban, Andreas Hejnol

**Affiliations:** Sars International Centre for Marine Molecular Biology, University of Bergen, Bergen, Norway; New York University, UNITED STATES

## Abstract

Most bilaterian animals excrete toxic metabolites through specialized organs, such as nephridia and kidneys, which share morphological and functional correspondences. In contrast, excretion in non-nephrozoans is largely unknown, and therefore the reconstruction of ancestral excretory mechanisms is problematic. Here, we investigated the excretory mode of members of the Xenacoelomorpha, the sister group to Nephrozoa, and Cnidaria, the sister group to Bilateria. By combining gene expression, inhibitor experiments, and exposure to varying environmental ammonia conditions, we show that both Xenacoelomorpha and Cnidaria are able to excrete across digestive-associated tissues. However, although the cnidarian *Nematostella vectensis* seems to use diffusion as its main excretory mode, the two xenacoelomorphs use both active transport and diffusion mechanisms. Based on these results, we propose that digestive-associated tissues functioned as excretory sites before the evolution of specialized organs in nephrozoans. We conclude that the emergence of a compact, multiple-layered bilaterian body plan necessitated the evolution of active transport mechanisms, which were later recruited into the specialized excretory organs.

## Abstract 2

Most animals possess specialized excretory organs to deposit toxic waste products from their body; others are assumed to use passive diffusion via the skin. However, investigation of one cnidarian and two acoelomorph species, which do not possess excretory organs, shows that the gut tissue is the most active excretory site and that acoelomorphs use active transport.

## Introduction

**Fig 1:**
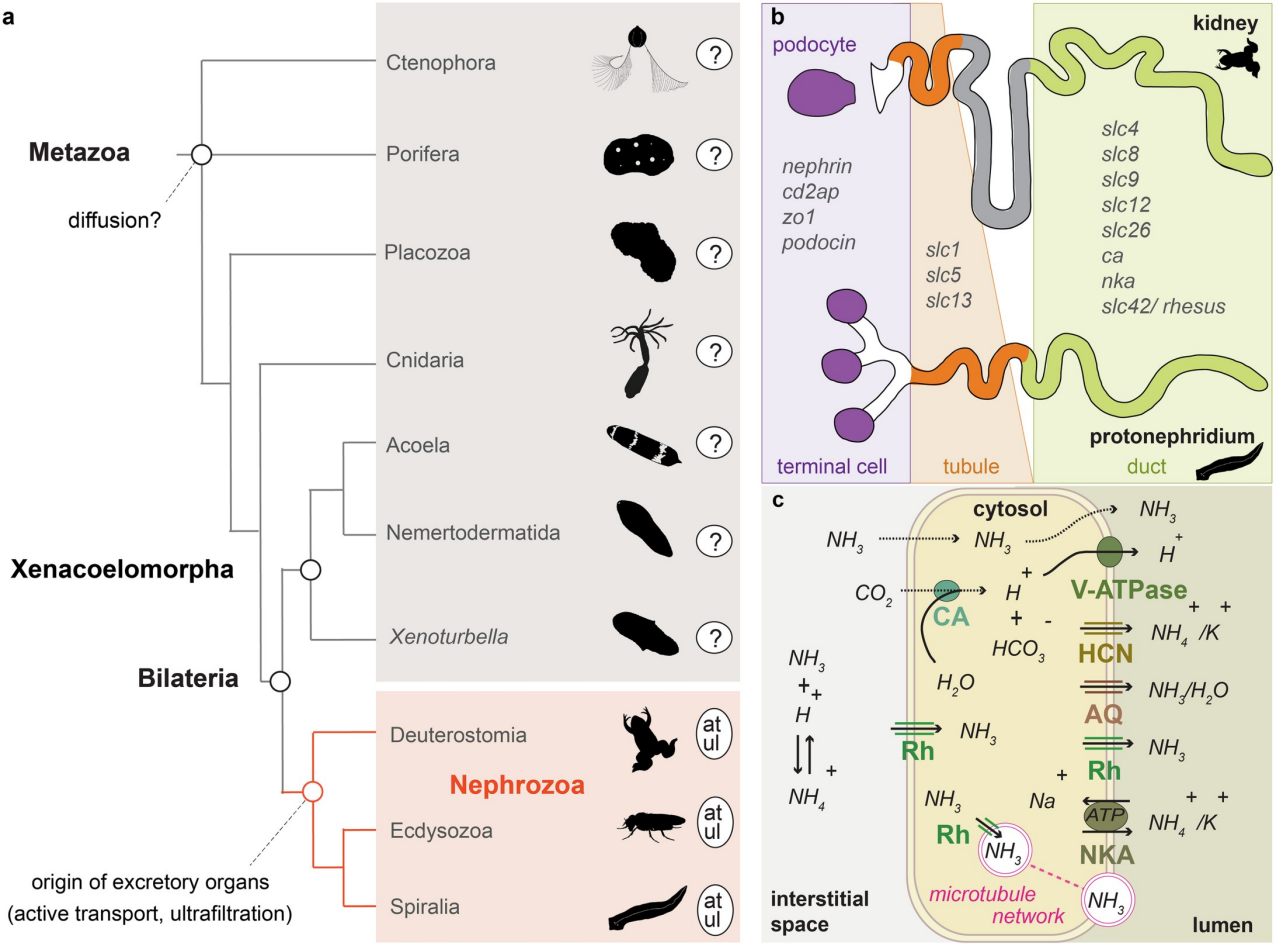
Traditional diffusion hypothesis, ammonia transport mechanism, and structural and functional correspondences between protonephridial and metanephridial systems. (a) Illustrated phylogenetic relationship between Nephrozoa, Xenacoelomorpha, and non-bilaterians [13]. Excretory organs or specialized excretory cells/tissues using active transport and ultrafiltration are so far only reported in the group of Nephrozoa. (b) Cartoon depiction of the structural components of metanephridia (podocyte, duct, tubule) in comparison to protonephridia (terminal cell, duct, tubule) and summary of the expression domains of orthologous selected genes in relation to their components. (c) NH_3_ cellular transport. NH_3_ is secreted into the lumen fluid via parallel H^+^ and NH_3_ transport. This involves passive diffusion through the cell membrane (dashed lines), facilitated diffusion via the Rh, active transport via the NKA, the hyperpolarization-activated cyclic nucleotide-gated HCN, and AQ as well as the generation of H^+^ gradient by a v-ATPase and the CA, which transforms CO_2_ into H^+^ and HCO_3_^−^. Vesicular ammonia-trapping mechanism is also illustrated. at, active transport; AQ, aquaporin transporter; CA, carbonic anhydrase; cd2ap, CD2-associated protein; HCN, K^+^[NH4^+^] channel; NH_3_, ammonia; NKA, Na^+^/K^+^[NH4^+^] ATPase; Rh, Rhesus glycoprotein; slc, solute carrier transporter; ul, ultrafiltration; v-ATPase, vacuolar H^+^-ATPase proton pump; zo1, zonula occludens 1. *Animal depictions are from phylopic*.*org and are not copyright protected (Public Domain Mark 1*.*0 license)*.

Excretory organs are specialized organs that remove toxic metabolic waste products and control water and ion balance in animals based on the principles of ultrafiltration, active transport, and passive transport/diffusion [1]. They are only present in Nephrozoa (Deuterostomia + Protostomia) [2] (Fig 1a) and, based on morphological correspondences, can be grouped into two major architectural units: the protonephridia, only found in Protostomia, and the metanephridia, present in both Deuterostomia and Protostomia [3,4]. Both organs are organized into functionally similar compartments: the terminal cells of protonephridia and the podocytes associated to metanephridial systems conduct ultrafiltration, and the tubule and duct cells modify the filtrate through a series of selective reabsorption and secretion, via passive and active transport mechanisms [5] (Fig 1b). There exist also other, taxon-specific excretory organs and excretory sites, which perform either ultrafiltration (such as the nephrocytes of insects [6] and the rhogocytes of gastropods [7]) or absorption and secretion (such as the malpighian tubules of various tardigrades, arachnids, and insects [8]; the excretory cells of nematodes [9]; the gills of fish, shore crabs, and annelids [10,11]; and the epidermis of planarians [12]).

Molecular studies have shown that a suite of orthologous genes is involved in the excretory mechanisms of different nephrozoan species, regardless of whether they possess specialized excretory organs [9–12,14–31] (see also S1 Table). The passive ammonia transporters Rhesus/AMTs, the active transporter Na^+^/K^+^[NH_4_^+^] ATPase (NKA), the hyperpolarization-activated cyclic nucleotide-gated K^+^[NH_4_^+^] channels (HCN), the vacuolar H^+^-ATPase (v-ATPase subunits A and B), members of the alpha-carbonic anhydrase (CA) group, and the water/glycerol/ammonia channels (aquaporins) are commonly used for excreting ammonia, the most toxic metabolite (Fig 1c) (summarized in [5,32]). Also, a group of orthologous slit diaphragm structural components (nephrin/kirre, CD2-associated protein [cd2ap], zonula occludens 1 [zo1], stomatin/podocin), whose function is associated with the maintenance of the ultrafiltration apparatus by interacting with the actin cytoskeleton and forming tight junctions [33], is localized at the ultrafiltration site of the podocytes of the rodent kidney [34] (Fig 1b) as well as at the *Drosophila* nephrocytes [35] and the rhogocytes of gastropods [7]. Finally, a number of ion transporters (solute carrier transporters [SLCs]) are spatially expressed in the corresponding compartments of protonephridia of planarians and metanephridia (e.g., kidneys) of vertebrates [36–38] (Fig 1b).

The excretory sites and mechanisms in non-nephrozoans, however, are largely unknown. It is commonly stated that excretion is presumably occurring via diffusion across the body wall because of the loose (e.g., sponges) or single-epithelial (cnidarians and ctenophores) cellular organization of these animals [1,39,40] (Fig 1a) (herein stated as “diffusion hypothesis”). Based on this idea, it was hypothesized that the emergence of the first excretory organs coincided with the evolution of multilayered, solid parenchymes and increased body sizes because of the need of more elaborate excretory mechanisms [41,42]. However, because excretion in non-nephrozoans was never investigated in detail, the ancestral mechanisms of excretion and the evolutionary origin of excretory organs remain unresolved [1,41–46].

An important animal group for our understanding nephrozoan evolution is their bilaterian sister group [2,13,47], the Xenacoelomorpha (*Xenoturbella* + [Nemertodermatida + Acoela]). These small, worm-like animals exhibit a bilaterally symmetric, multilayered body plan, but except for a special cell type with a putative excretory function (dermonephridia) [48] that seems unique to the acoel *Paratomella*, xenacoelomorphs lack excretory organs and no defined excretory sites have yet been described. To understand the excretory mechanisms outside Nephrozoa and gain insights into ancient excretory mechanisms, we therefore investigated the excretory modes of two xenacoelomorph species and compared our findings with the non-bilaterian cnidarian *Nematostella vectensis*.

## Results

### Most genes involved in excretion in nephrozoa are already present in non-nephrozoan and non-bilaterian animals

To get an overview of the presence of excretion-related genes in xenacoelomorphs and non-bilaterian animals, we first searched for the orthologous sequences of 20 nephrozoan candidate genes in the available transcriptomes and draft genomes of 13 xenacoelomorph species as well as in representatives of cnidarians, placozoans, and sponges (S1a and S2 Figs). We found that most of these genes were present in almost all groups with the exceptions of *slc5* (a sodium glucose cotransporter), which was only present in Cnidaria and Bilateria, and the ultrafiltration component *nephrin*/*kirre*, which was only present in Bilateria (S1 Table, S1a Fig). This analysis also revealed that the last common ancestor of placozoans, cnidarians, and bilaterians already had at least two paralogs of *amts* (*amt2/3* and *am1/4*) and one *rhesus*, with independent duplications of one or both of these genes in various animal lineages (S2 Fig). To identify potential excretory sites in xenacoelomorphs, we examined the expression of the entire set of these candidate excretion-related genes in the acoel *Isodiametra pulchra* and the nemertodermatid *Meara stichopi*, which differ in their digestive system composition (*I*. *pulchra* has a syncytial, lumenless gut, whereas *M*. *stichopi* has an epithelia-lined, cellular gut [49] (S1b Fig).

### Genes encoding slit diaphragm–related components and SLCs are expressed broadly in *I*. *pulchra* and *M*. *stichopi*

Genes related to ultrafiltration sites (*nephrin*/*kirre*, *cd2ap*, *zo1*, *stomatin/podocin*) and SLCs (*slc1*, *slc4*, *slc5*, *slc8*, *slc9*, *slc12*, *slc13*, *slc26*) were broadly expressed within neural (brain and nerve cords), parenchymal/subepidermal, digestive, and gonadal-associated cells in both animals (S3 and S4 Figs). A summary of these expression patterns is summarized in Table 1. The broad expression of ultrafiltration-related components and SLCs in acoelomorphs shows that they are not part of defined excretory domains, as in nephrozoans, thus suggesting that the spatial arrangement of these genes resulting in the formation of specialized excretory compartments (e.g., nephridial compartments) took place in the nephrozoan lineage.

**Table 1:** Summary of expression patterns of excretion-related genes in *I*. *pulchra* and *M*. *stichopi*.

	*I*. *pulchra*	*M*. *stichopi*
*nephrin/kirre*	*nephrin/kirre1*: brain, male gonopore	*nephrin/kirre1*: proximal lateral rows (nerve cords?)
	*nephrin/kirre2*: scattered cells (neurons?)	*nephrin/kirre2*: proximal lateral rows (nerve cords?)
	*nephrin/kirre3*: scattered cells (neurons?)	*nephrin/kirre3*: proximal lateral rows (nerve cords?)
*cd2ap*	scattered cells (neurons?)	subepidermal cells, mouth, posterior lateral rows of cells
*zo1*	scattered cells (neurons?), mouth, anterior cells (brain?)	gut-affiliated cells, mouth
*stom/pod*	*stom/pod a*: brain	*stom/pod a*: subepidermal cells, proximal lateral rows (nerve cords?)
	*stom/pod b*: digestive syncytium	*stom/pod b*: subepidermal cells, mouth
	*stom/pod c*: brain	*stom/pod c*: subepidermal cells
		*stom/pod d*: subepidermal cells
		*stom/pod e*: proximal lateral rows (nerve cords?)
*rhesus*	anterior cells, gut-affiliated cells, posterior ventral epidermis	
*hcn*	brain	gut-affiliated cells
*amts*	*amt-like*: brain	epidermis
	*amt1/4 a*: brain, mouth	
	*amt1/4 b*: parenchymal cells	
	*amt2/3 a*: scattered cells (neurons?)	
	*amt2/3 b*: brain, parenchyme	
	*amt2/3 c*: scattered cells (neurons?)	
*nka*	*nka a*: gut-wrapping cells	gut-affiliated cells
	*nka b*: gut-wrapping cells	
*v-ATPase B*	digestive syncytium	*v-ATPase B1*: gut-affiliated cells, proximal lateral rows (nerve cords?)
		*v-ATPase B2*: gut-affiliated cells, subepidermal cells
*alpha-ca*	*ca a*: anterior cells, male gonopore, mouth, parenchymal cells	*ca a*: gut-affiliated cells
	*ca b*: scattered cells (neurons?)	*ca b*: gut-wrapping cells
	*ca c*: scattered cells (neurons?)	*ca c*: gut-affiliated cells
	*ca d*: anterior cells, parenchymal cells	*ca d*: scattered cells (neurons?), posterior epidermis
	*ca h*: scattered cells (neurons?)	
	*ca x*: brain	
*aqs*	*aq a*: brain	*aq a*: proximal lateral rows (nerve cords?)
	*aq b*: digestive syncytium	*aq b*: scattered cells (neurons?)
	*aq c*: gut-wrapping cells, scattered cells (neurons?)	*aq c*: proximal lateral rows (nerve cords?)
	*aq e*: parenchymal cells	*aq d*: gut-wrapping cells
	*aq f*: gut-wrapping cells, scattered cells (neurons?)	*aq e*: anterior cells, scattered cells (neurons?)
	*aq g*: gut-wrapping cells, female gonads	*aq f*: gut-wrapping cells
*slc1*	*slc1a*: brain	scattered cells (neurons?)
	*slc1b*: male gonopore	
	*slc1c*: brain, male gonopore	
*slc4*	*slc4a*: anterior cells	*slc4a*: gut-wrapping cells, mouth
	*slc4b*: brain, parenchymal cells	*slc4b*: nerve cords
	*slc4c*: anterior cells, parenchymal cells	*slc4c*: subepidermal cells, female gonads
*slc5*	*slc5a*: male gonopore	subepidermal cells, posterior lateral rows of cells
	*slc5b*: brain, parenchymal cells	
*slc8*	anterior cells	gut epithelium, mouth, posterior lateral rows of cells
*slc9*	mouth	proximal lateral rows (nerve cords?)
*slc12*	*slc12a*: brain, parenchymal cells	*slc12a*: male gonads, female gonads
	*slc12b*: brain, parenchymal cells	*slc12b*: male gonads
*slc13*	brain, parenchymal cells	*slc13b*: scattered cells (neurons?)
		*slc13c*: scattered cells (neurons?)
		*slc13d*: scattered cells (neurons?)
*slc26*	no expression revealed	*slc26a*: female gonads
		*slc26b*: gut-wrapping cells

Abbreviations: *amt*, ammonia transporter; *aq*, aquaporin; *ca*, carbonic anhydrase; *cd2ap*, CD2-associated protein; *hcn*, K^+^[NH4^+^] channel; *pod*, podocin; *slc*, solute carrier transporter; *stom*, stomatin; *v-ATPase*, vacuolar H^+^-ATPase proton pump; *zo1*, zonula occludens 1

### The expression of a number of ammonia excretion–related genes and aquaporins suggests digestive-associated domains as putative excretion sites in *I*. *pulchra* and *M*. *stichopi*

**Fig 2:**
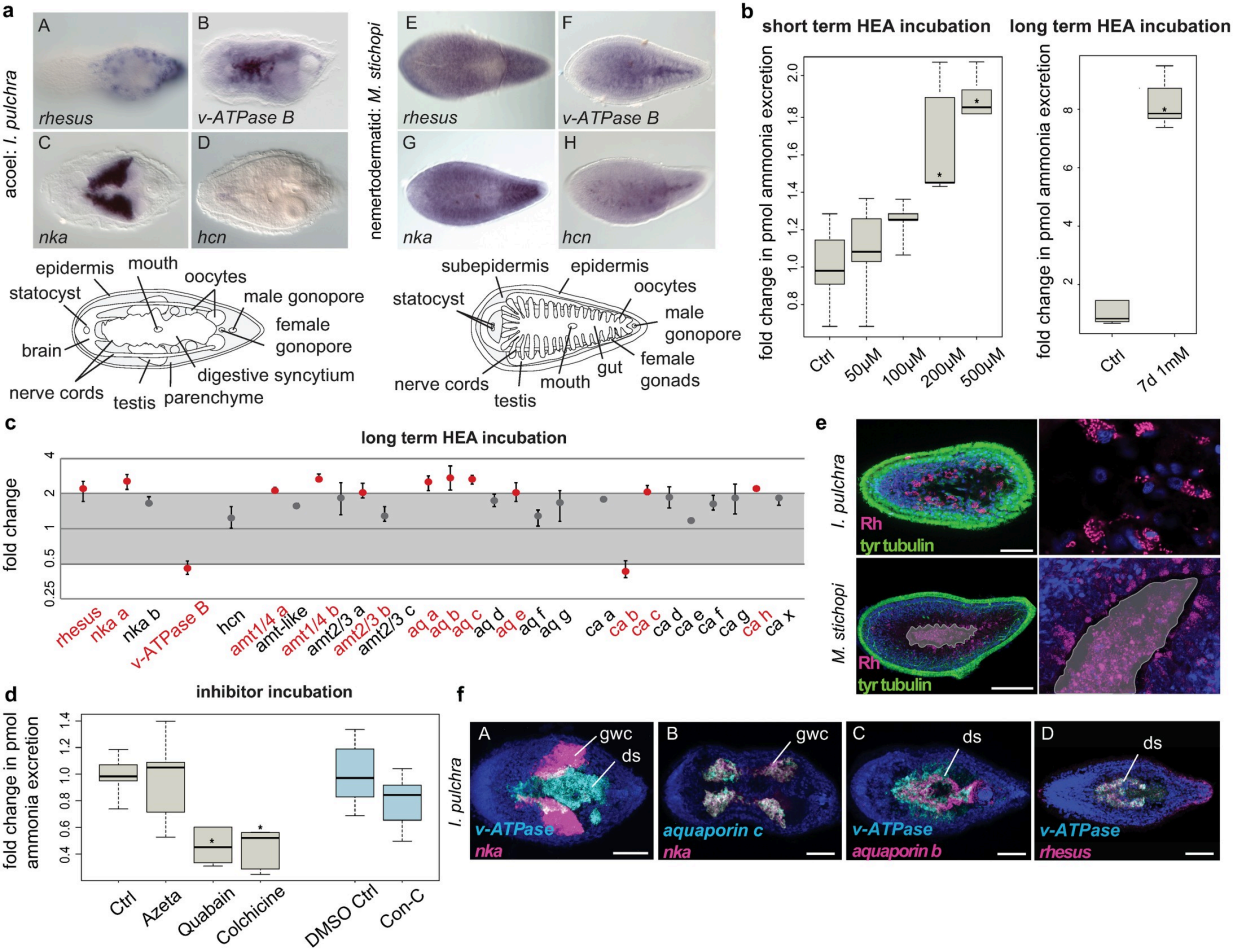
Excretion in acoelomorphs. (a) WMISH of *rhesus*, *v-ATPase*, *nka*, and *hcn* in *I*. *pulchra* and *M*. *stichopi*. (b) Ammonia excretion rates of *I*. *pulchra* before (Ctrl) and after exposure for 2 hours to 50, 100, 200, and 500 μM and after exposure for 7 days in 1 mM NH_4_Cl (boxplot). Excretion was measured over 2 hours following the HEA treatments in at least three independent biological replicates, each divided into two separate samples (six measurements in total). Bold horizontal bars in boxes indicate the median; lower and upper box borders indicate lower and upper quartile; and whiskers indicate minimum and maximum. Asterisks label significant changes (*p* < 0.02 in an unpaired, 2-tailed *t* test with unequal variance). (c) Quantitative relative expression of *rhesus*, *nka*, *v-ATPase B*, *amts*, *aq*, and *ca* after 7 days of exposure in HEA (1 mM NH_4_Cl). Each circle indicates the average of three independent biological replicates, each with four technical replicates. Error bars indicate minimum and maximum of the biological replicates (averaged technical replicates). A 1-fold change represents no change; ≥2 indicates significantly increased expression level; ≤0.5 indicates significantly decreased expression level (red labels). (d) Effects of different inhibitors on ammonia excretion rates in *I*. *pulchra* (boxplot, with illustration and replicates similar to Fig 2b). The concentrations used were 5 μM Con-C as a v-ATPase A/B inhibitor, 1 mM azetazolamide as an inhibitor of the CA, 1 mM quabain as an NKA inhibitor, and 2 mM colchicine for inhibiting the microtubule network. Con-C was diluted in 0.5% DMSO for which we used an appropriate Ctrl with 0.5% DMSO. (e) Protein localization of Rhesus in *I*. *pulchra* and *M*. *stichopi*. Syncytium and gut are indicated in gray, and the magenta staining of the lumen in *M*. *stichopi* is false-positive staining of the gut content. Fluorescent pictures are projections of merged confocal stacks. The nervous system is stained green with tyr tubulin. (f) Double fluorescent WMISH of *v-ATPase* and *nka*, *aq c* and *nka*, *v-ATPase* and *aq b*, and *v-ATPase* and *rhesus* in *I*. *pulchra*. White areas in the first panel are the result of merged stacks and not of overlapping expression. Nuclei are stained blue with DAPI. Anterior is to the left. Scale bars are 50 μm for *I*. *pulchra* and 100 μm for *M*. *stichopi*. Values underlying panels b and d are provided in S6 Table, and values underlying panel c are provided in S4 Table. amt, ammonia transporter; aq, aquaporin; CA, carbonic anhydrase; Con-C, concanamycin C; Ctrl, control; DAPI, 4',6-diamidino-2-phenylindole; ds, digestive syncytium; gwc, gut-wrapping cell; HCN, K^+^[NH4^+^] channel; HEA, high environmental ammonia; NKA, Na^+^/K^+^[NH4^+^] ATPase; Rh, Rhesus glycoprotein; slc, solute carrier transporter; tyr, tyrosinated; v-ATPase, vacuolar H^+^-ATPase proton pump; WMISH, whole-mount in situ hybridization.

Genes involved in ammonia excretion (*rhesus*/*amts*, *nka*, *v-ATPase B*, *ca*, *hcn*) and *aquaporins* were mainly demarcating neural, digestive-associated, and other parenchymal/subepidermal cells, as well as epidermal cells (Fig 2a, S4 and S5 Figs). A summary of these expression patterns is summarized in Table 1. The expression of ammonia excretion–related genes and *aquaporins* shows that these genes do not label demarcated excretory domains. However, because transcripts of the ammonium transporters *rhesus*, *nka*, and *hcn* (only in *M*. *stichopi*), the proton exchanger *v-ATPase*, as well as a number of *cas* and *aquaporins*, were found in association with the digestion system, the possibility that digestive-associated tissues could act as excretory sites was raised.

### High environmental ammonia exposure indicates a diffusion mechanism in *I*. *pulchra*

To reveal the excretory mechanism in xenacoelomorphs, we conducted high environmental ammonia (HEA) incubation experiments, as previously performed in a large array of animals (summarized in [5,50]), using *I*. *pulchra* because of its availability in sufficient numbers. We first measured the pH of incubation mediums with different HEA concentrations (up to 1 mM) and found no difference in pH, which could otherwise have influenced any excretion rates. We then exposed animals to different HEA concentrations for a short period (2 hours) and measured the ammonia excretion during the following 2 hours, after bringing them back into normal conditions, to test excretion via diffusion. The ammonia excretion rates of exposed animals remained unchanged after exposure to 50 and 100 μM NH_4_Cl, compared with the control conditions, but increased gradually after exposure to NH_4_Cl concentrations of 200 and 500 μM NH_4_Cl (Fig 2b). The increase in ammonia excretion rate could be explained by a concentration-dependent ammonia uptake during the HEA exposure and a subsequent release in normal conditions. These results suggest that ammonia excretion is concentration-dependent, which is indicative of a diffusion mechanism.

### HEA exposure influences the expression of some excretion-related genes in *I*. *pulchra*

To test for a possible involvement of the excretion-related genes in the excretory mechanism of xenacoelomorphs, we tested for alteration of mRNA expression levels in chronically HEA-exposed animals by quantitative relative expression experiments (quantitative PCR [qPCR]) in *I*. *pulchra*. We first exposed animals to 1 mM HEA for 7 days, similar to conditions used in previous studies (summarized in [5,50]), and measured the ammonia excretion over 2 hours after bringing the animals into normal conditions. As expected, the ammonia excretion rates were strongly increased, in line with the short-term HEA-exposure experiments (Fig 2b). When we tested the expression level of excretion-related genes, we found that the expression of the passive ammonia transporters *rhesus* and three *amts*, as well as the active ammonia transporter *nka*, altered significantly (Fig 2c). Other differentially expressed genes were four *aquaporins*, the *v-ATPase*, and three *cas* (Fig 2c). These results indicate a putative role of these genes in ammonia excretion and suggest that acoels might not only excrete by diffusion and via passive transporters (*rhesus*, *amts*) but also by an alternative active transport mechanism (*nka*).

### Inhibitor experiments support an active excretion mechanism via NKA transporter, as well as a passive vesicular transport mode, possibly mediated by Rhesus transporter

We further tested the involvement of NKA, V-ATPase A/B, and CA proteins in excretion, as well as a possible involvement of a vesicular transport mechanism, by conducting pharmacological inhibitor assays in *I*. *pulchra*, as previously demonstrated in other animals (summarized in [5,50]). Inhibition of the CA by azetazolamide did not show any significant change in ammonia excretion. Inhibition of the V-ATPase by concanamycin C seemed to lead to a decrease in ammonia excretion, although a 2-tailed *t* test did not support a significant change. In contrast, when perturbing the function of NKA with quabain, the ammonia excretion dropped significantly (Fig 2d), which further supports an active excretion mechanism via NKA, similar to what is described for many nephrozoans [10–12,14,17,19,20,25–27,31,51–53]. Interference with the vesicular transport using colchicine also led to a significant decrease in ammonia excretion, indicating a possible vesicular ammonia-trapping excretion mode (Fig 1c), as demonstrated in the midgut epithelium of the tobacco hornworm [26], the gills of the shore crab [27], and the integument of the nematode [14]. To test whether vesicular transport might occur through Rhesus transporter as shown in other studies (summarized in [5,50]), we revealed Rhesus protein localization by immunohistochemistry (Fig 2e). The protein localization mimicked the gene expression and revealed, apart from cells at the anterior tip and cells of the posterior epidermis, individual parenchymal cells affiliated with the digestive syncytium that extend ventrally. Higher magnification showed that the transporter was present in cytoplasmic vesicles and not on the cellular membrane (Fig 2e). This further indicated the presence of a vesicular transport mechanism, in which cellular ammonia moves via Rhesus transporters into vesicles and gets transferred to the membrane through the microtubule network [54] (Fig 1c). The antibody specificity was confirmed by an alignment of the epitope and the endogenous protein, as a well as a western blot analysis (S6 Fig). Similar vesicular protein localization was also observed in *M*. *stichopi*, suggesting a similar cytoplasmic–vesicular role of Rhesus transporter in gut-affiliated cells, also in nemertodermatids (Fig 2e). These data further supported the involvement of NKA and Rhesus transporters in ammonia excretion and also indicated the presence of a putative vesicular transport mode in *I*. *pulchra*.

### Double fluorescent whole-mount in situ hybridization of differentially expressed genes shows similar spatial arrangement in gut-associated domains in *I*. *pulchra* and *M*. *stichopi*

To obtain a better resolution and understanding of the relative topology of the differentially expressed genes, double fluorescent whole-mount in situ hybridization (WMISH) was conducted for *v-ATPase*, *nka*, *aquaporins b* and *c*, and *rhesus* (Fig 2f, S4 Fig). *Nka* and *v-ATPase* were expressed in a mutually exclusive manner, with *v-ATPase* to be restricted in the ventral region digestive syncytium and *nka* in an adjacent parenchymal, distal sac-shaped gut-wrapping domain (Fig 2fA, S4C Fig). The expression of *nka* was not extending to the male gonads (testes) (S4A Fig). *Aquaporin c* was coexpressed with *nka* (Fig 2fB), and *aquaporin b* was partially overlapping with *v-ATPase*, with *aquaporin b* expression extending into the posterior region of the digestive syncytium (Fig 2fC). Finally, *rhesus* was partly coexpressed with *v-ATPase* in the ventral region of the digestive syncytium (Fig 2fD). Similar coexpression analysis of the orthologous genes was also conducted for *M*. *stichopi* and revealed striking similarities in their spatial arrangement to *I*. *pulchra* (S4 and S7 Figs). *v-ATPase* expression was not overlapping with *nka*, as *v-ATPase* was restricted to the gut epithelium and in two proximal lateral rows of subepidermal cells, whereas *nka* was limited to cells lining the distal part of the epithelial branches of the gut extending toward the subepidermis (S4E and S7 Figs). *v-ATPase* was partly coexpressed with *rhesus* in ventral gut-affiliated cells (S7 Fig). Overall, these data revealed a similar spatial arrangement in gut-associated domains in both animals, which seems to be unrelated to the presence of an epithelial gut or a syncytium. However, given the fact that *I*. *pulchra* has a lumenless digestive tissue, ammonia is probably accumulated intracellularly in the syncytium before it gets expelled via the mouth, whereas in the case of *M*. *stichopi*, ammonia gets released in the gut lumen.

Taken together, our findings suggest that *I*. *pulchra* uses different mechanisms for ammonia excretion that are also known from nephrozoans; an active ammonia excretion mechanism via NKA through the digestive system, as suggested by in situ hybridization, and a passive vesicular transport mechanism likely mediated by Rhesus, through digestive and likely also epidermal tissues. Given the commonalities in the expression of the involved genes in both animals, these excretory mechanisms could be plesiomorphic for acoelomorphs.

### HEA experiments suggest a diffusion mechanism also in the cnidarian *N*. *vectensis*

Because our results showed the involvement of active and passive transport mechanisms across digestive tissues outside Nephrozoa, we also investigated a non-bilaterian species, the cnidarian *N*. *vectensis* (S1b Fig), to test whether this excretion mode might also be present outside Bilateria. The only available excretion studies in cnidarians are few morphological studies, which suggested that the septa filaments of the anthozoan mesenteries and the radial canals of medusozoans could serve as putative excretory sites [55], as well as some isotopic exchange experiments in *Hydra oligactis* that have shown that the gastrodermis seems to be involved in osmoregulation [56]. Moreover, there is evidence that Rh and AMT transporters are generally involved in ammonium excretion in corals [57], but localization studies that would suggest excretion sites are missing.

**Fig 3:**
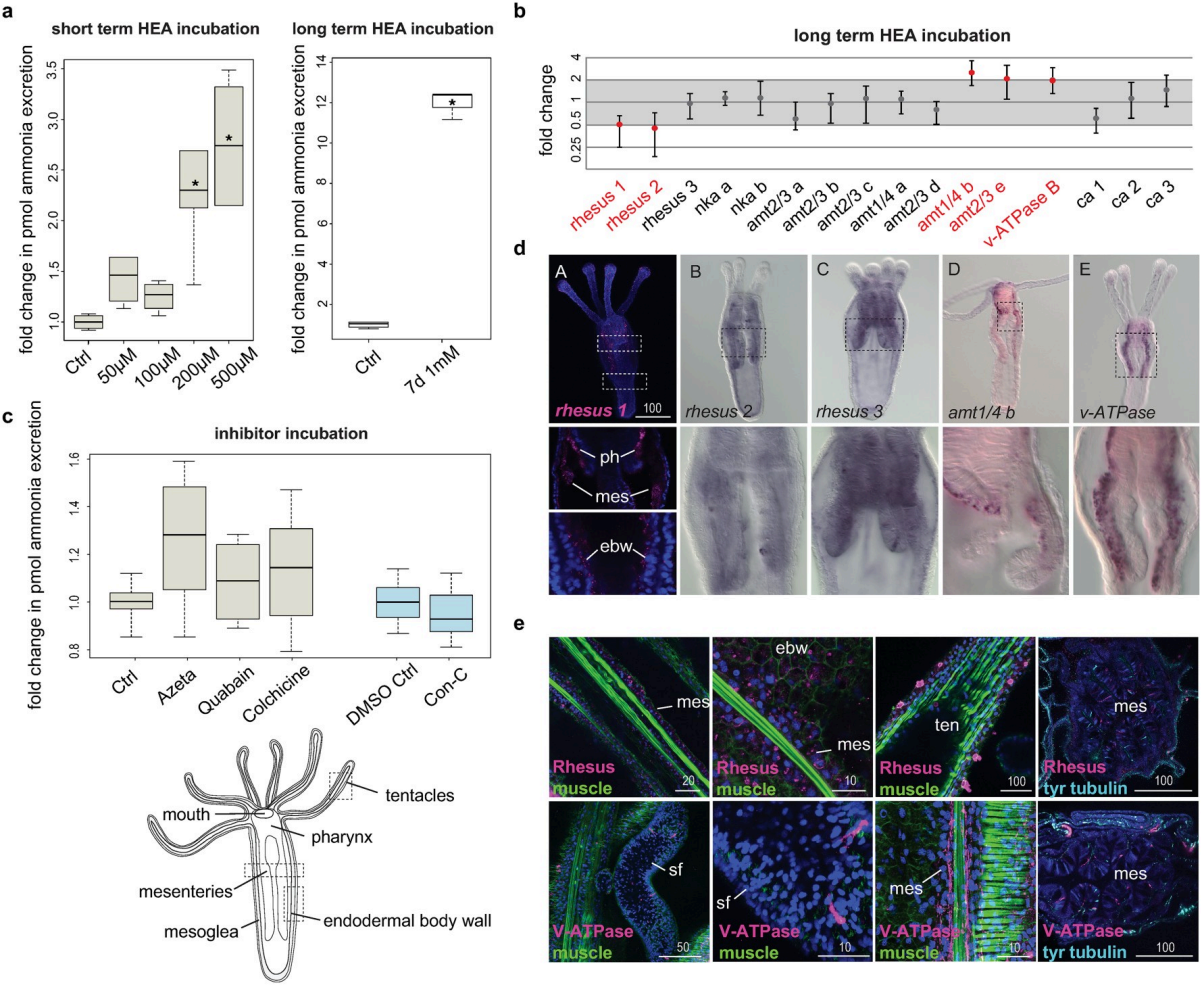
Excretion in *N*. *vectensis*. (a) Ammonia excretion rates of *N*. *vectensis* before (Ctrl) and after exposure for 2 hours to 50, 100, 200, and 500 μM and after exposure for 7 days in 1mM NH_4_Cl (boxplot). Excretion was measured over 2 hours following the HEA treatments in at least three independent biological replicates, each divided into two separate samples (six measurements in total). Bold horizontal bars in boxes indicate the median; lower and upper box borders indicate lower and upper quartile; and whiskers indicate minimum and maximum. Asterisks label significant changes. Significance, *p* < 0.02 (unpaired *t* test with unequal variance). (b) Quantitative relative expression of *rhesus*, *nka*, *v-ATPase B*, *amts*, and *ca* after exposure for 7 days in HEA (1 mM NH_4_Cl). Each circle represents the average of five independent biological replicates, each with three technical replicates. A 1-fold change represents no change; ≥2 indicates increased expression level significantly; ≤0.5 indicates decreased expression level significantly (red labels). (c) Effects of different inhibitors on ammonia excretion rates in *N*. *vectensis* (boxplot, with illustration and replicates similar to Fig 2d). The concentrations used were 5–15 μM Con-C as a V-ATPase A/B inhibitor, 1–3 mM azetazolamide as an inhibitor of the CA, 1–5 mM quabain as an NKA inhibitor, and 2–10 mM colchicine for inhibiting the microtubule network. Quabain was diluted in 0.5% DMSO, for which we used an appropriate Ctrl with 0.5% DMSO. *N* = 3 for all treatments. (d) Whole-mount in situ hybridization of *rh 1*, *rh 2*, *rh 3*, *v-ATPase*, and *amt1/4b* in feeding primary polyps. Anterior is to the top. (e) Protein localization of Rh and v-ATPase in *N*. *vectensis* early-juvenile polyps. The muscle filaments are labeled green with phalloidin, and the nervous system is stained cyan with tyr tubulin. Every picture is a full projection of merged confocal stacks. Nuclei are stained blue with DAPI. The regions shown are indicated with dashed boxes in the illustrated animal. Values underlying panels a and c are provided in S6 Table, and values underlying panel b are provided in S4 Table. amt, ammonia transporter; CA, carbonic anhydrase; Con-C, concanamycin C; Ctrl, control; DAPI, 4',6-diamidino-2-phenylindole; ebw, endodermal body wall; HEA, high environmental ammonia; mes, mesenteries; nka, Na^+^/K^+^[NH4^+^] ATPase; ph, pharynx; rh, Rhesus glycoprotein; sf, septal filament; ten, tentacles; tyr, tyrosinated; v-ATPase, vacuolar H^+^-ATPase proton pump.

We first tested whether *N*. *vectensis* excretes via diffusion by exposing early-juvenile animals to HEA for 2 hours and measuring their ammonia excretion rates afterward, similar to the experiments performed with *I*. *pulchra*. We found that, also in *N*. *vectensis*, ammonia excretion increased significantly after HEA exposure starting at 200 and 500 μM NH_4_Cl (Fig 3a). Measurements of the pH of each incubation medium showed that the pH dropped by 0.2 when the medium contained 500 μM NH_4_Cl. However, when we measured the excretion of animals over 2 hours in a medium with an accordingly lowered pH, we found that a difference of 0.2 did not change the excretion rates (S2 Table). Therefore, the increase in ammonia excretion rates at 200 and 500 μM NH_4_Cl indicates that ammonia excretion is concentration-dependent, supporting a diffusion mechanism also in *N*. *vectensis*.

### Quantitative gene expression and inhibitor experiments indicates an involvement of passive but not active transport mechanisms in *N*. *vectensis*

We then exposed animals to 1 mM HEA for 7 days and tested the expression of the orthologous genes altered in *I*. *pulchra* treatments (*rh/amt*, *nka*, *v-ATPase B*, and *ca*) by qPCR. As expected from the short-term HEA-exposure experiment, specimens exposed for 7 days in the HEA condition showed increased ammonia excretion rates (Fig 3a). In contrast to *I*. *pulchra*, none of the two *nka* transporters showed a significant change in gene expression in animals exposed to HEA for 7 days (Fig 3b). However, the expression of the passive transporters *rhesus1*, *rhesus2*, *amt1/4b*, and *amt2/3e*, as well as *v-ATPase*, altered significantly (Fig 3b), indicating the putative involvement of these transporters in excretion of *N*. *vectensis*. To test whether Rhesus acts via a vesicular transport mechanism, we conducted the same pharmacological experiment as in *I*. *pulchra*. Contrary to the results from acoels, we found that inhibition of vesicular transport did not alter the ammonia excretion (Fig 3c). We also inhibited the excretory function of V-ATPase and CA proteins and found that none of them showed any significant change in ammonia excretion rates (Fig 3c). Finally, when we perturbed the function of NKA, the ammonia excretion rates did not alter (Fig 3c), confirming the qPCR results (Fig 3b) and further supporting the non-involvement of the NKA transporter in excretion. These results suggest that the ammonia excretion of *N*. *vectensis* is likely mediated by the passive Rhesus and AMT transporters, but neither relies on active transport mechanism mediated by NKA or on vesicular ammonia-trapping excretion mode.

### Gene expression of excretion-related genes reveals the gastrodermis as excretory site in *N*. *vectensis*

To understand whether these genes were expressed in gastrodermal or epidermal cells, we revealed the spatial expression of *rhesus*, *amts*, *nka*, and *v-ATPase B* by WMISH in feeding primary polyps. All genes were mainly demarcating gastrodermal domains, such as the endodermal body wall, the directive mesenteries, septal filaments, and the pharynx (Fig 3d, S8 Fig). *Rhesus 1* was additionally expressed in the tentacular ectoderm (Fig 3dA). Protein localization of Rh, NKA, and V-ATPase B reflected the transcript expression patterns (Fig 3e, S9 Fig). High magnification of Rhesus antibody staining further revealed that the transporter was not expressed in cytoplasmic vesicles, supporting a non-vesicular transport mechanism, in agreement with the inhibitor experiments. Also, it showed that Rhesus was localized in individual cells of the tentacular ectoderm with clumped structures at the tentacle surface, which resembled gland cells [58] (Fig 3e). The NKA antibody was localized in endodermal neurons and individual cells of the mesenteries, likely neural precursors (S9 Fig), thus suggesting a non-excretory function of this transporter, as indicated already from the qPCR and inhibitor experiments. These data imply that gastrodermis-affiliated domains likely serve as excretory sites in *N*. *vectensis*.

## Discussion

Overall, our findings show that acoelomorphs use, in addition to diffusion, active transport mechanisms, in contrast to what has been previously assumed for non-nephrozoans [1,39,40]. Our results also suggest that excretion takes place across digestive tissues and likely also across the epidermis, as indicated from the *rhesus* (both animals) and *amt* (only in *M*. *stichopi*) expression. *N*. *vectensis* also seem to use gastrodermal tissues as excretory sites, but we were not able to detect any active transport mechanism. However, we do not know whether the absence of active transport we find in *N*. *vectensis* is true for all cnidarians. It has been shown that differences in morphology and ecology (e.g., size and activity) are related with interspecific differences in excretion rates [59,60]. Therefore, bigger and more active cnidarian species might require more efficient modes of excretion, such as an active transport, in order to fulfill their metabolic requirements. Only more studies in other cnidarian species can elucidate this issue.

**Fig 4:**
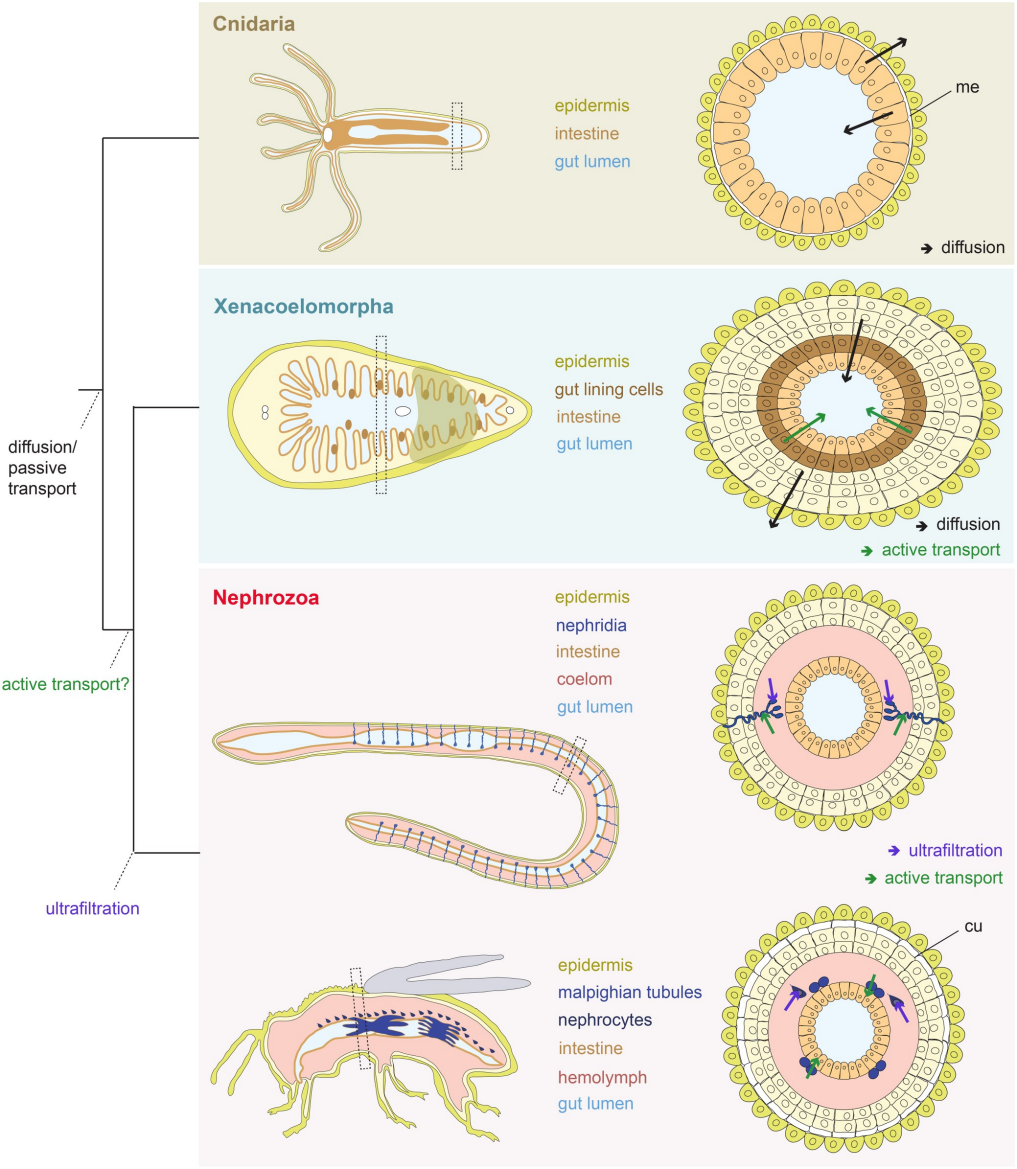
Evolution of excretory mechanisms. Illustration of the proposed direction of fluxes in Cnidaria and Xenacoelomorpha and evolution of active ammonia transport and ultrafiltration mechanisms. Cnidaria (e.g., *N*. *vectensis*) excrete across their intestinal epithelium (and probably across the epidermis too) via diffusion, whereas in xenacoelomorphs, excretion occurs both via diffusion across the epidermis and gut-associated tissues and via active transport across gut-associated tissues. Ultrafiltration mechanism originated within Nephrozoa. cu, cuticle; me, mesoglea.

Digestive tissues with additional or assigned excretory roles have also been reported in several nephrozoans (e.g., vertebrates, annelids, insects, nematodes, tunicates, chaetognaths) [16,26,31,61–67]. In the light of our results, this excretion mechanism likely reflects an ancient mechanism, before the evolution of specialized organs, such as nephridia (S10 Fig). The molecular spatial arrangement of the excretion sites in non-nephrozoans, however, is not sharing topological arrangements with common nephridial domains of nephrozoans (Fig 1b), suggesting that they are evolutionarily unrelated to nephridia. It still remains unclear whether these domains are multifunctional or consist of specialized excretory subdomains; however, a degree of cell subfunctionalization seems to be present, as indicated by the localized gene expression in different groups of gut-wrapping and gut epithelial cells. We can, however, exclude the presence of ultrafiltration sites, in agreement with previous ultrastructure studies in acoelomorphs [68], because the homologous essential molecular components of the ultrafiltration sites of nephridia and nephrocytes are mostly expressed in neural domains in acoelomorphs and are absent in non-bilaterians (nephrins), suggesting their later recruitment in the nephrozoan filtration apparatus (Fig 4).

Recently, a new study was published suggesting the non-monophyly of Deuterostomia and a placement of Ambulacraria (Echinodermata + Hemichordata) as the sister group of Nemertodermatida + (Acoela + *Xenoturbella*) [69]. If true, this novel topology has vast consequences for our understanding of the evolution of all major bilaterian organ systems including the excretory organs [70]. Based on this phylogeny, either excretory organs have been independently evolved in Ambulacraria and (Chordata + Protostomia) or they have been already present in the last common ancestor of Bilateria and got lost secondarily in nemertodermatids, acoels, and *Xenoturbella*. Ambulacraria possesses a metanephridial type of excretory organ, which, according to some authors, is independently evolved [43,71]; therefore, excretory organs of Ambulacraria, Chordata, and Protostomia might not be homologous. In the scenario of the presence of an excretory organ in the last common ancestor of Bilateria, one would have to assume a complete reduction of excretory organs in the lineage to Nemertodermatida + (Acoela + *Xenoturbella*) without morphological or molecular traces. However, the support values for the main branches of Bilateria in the [69] study are low, and the study also does not recover the Xenacoelomorpha as clade. In-depth analyses are necessary to test whether the new topology is not an artifact that is based on the new approach the study uses for the phylogenomic analyses.

To conclude, our study shows that active transport mechanism and excretion through digestive tissues predates the evolution of specialized excretory systems. Whether this is based on a convergent recruitment or reflects an ancestral state for Bilateria remains unclear. However, if the latter is true, it correlates with the emergence of multilayered body plans and solid internal parenchymes that separate the body wall from their digestive tract, as seen in xenacoelomorphs and nephrozoans. We thus propose that diffusion mechanisms were the major excretory modes present in animals with single-layered epithelial organization (Fig 4). The emergence of more complex, multilayered body plan necessitated an active transport of excretes, which was later recruited in specific compartments of the complex excretory organs in the lineage of Nephrozoa.

## Methods

No statistical methods were used to predetermine sample size. The experiments were not randomized. The investigators were not blinded to allocation during experiments and outcome assessment.

### Gene cloning and orthology assignment

Putative orthologous sequences of genes of interest were identified by tBLASTx search against the transcriptome (SRR2681926) of *I*. *pulchra*, the transcriptome (SRR2681155) and draft genome of *M*. *stichopi*, and the genome of *N*. *vectensis* (http://genome.jgi.doe.gov). Additional transcriptomes of Xenacoelomorpha species investigated were as follows: *Childia submaculatum* (Acoela) (SRX1534054), *Convolutriloba macropyga* (Acoela) (SRX1343815), *Diopisthoporus gymnopharyngeus* (Acoela) (SRX1534055), *Diopisthoporus longitubus* (Acoela) (SRX1534056), *Eumecynostomum macrobursalium* (Acoela) (SRX1534057), *Hofstenia miamia* (Acoela) (PRJNA241459), *Ascoparia* sp. (Nemertodermatida) (SRX1343822), *Nemertoderma westbladi* (Nemertodermatida) (SRX1343819), *Sterreria* sp. (Nemertodermatida) (SRX1343821), *Xenoturbella bocki* (*Xenoturbella*) (SRX1343818), and *Xenoturbella profunda* (Xenoturbella) (SRP064117). Data were deposited in the Dryad repository: https://doi.org/10.5061/dryad.bq068jr [72].

Sequences for the placozoan *Trichoplax adhaerens*, the sponge *Amphimedon queenslandica*, the ctenophore *Mnemiopsis leidy*, the protist *Capsaspora owczarzaki*, the amoeba *Dictyostelium discoideum*, and the nephrozoans *Homo sapiens*. *Saccoglossus kowalevskii*, *Strongylocentrotus purpuratus*, *Xenopus laevis*, *Branchiostoma lanceolatum*, *Capitella teleta*, *Crassostrea gigas*, *Lottia gigantea*, *Schmidtea mediterranea*, *Tribolium castaneum*, *Caenorhabditis elegans*, and *Drosophila melanogaster* were obtained from Uniprot and NCBI public databases. Gene orthology of genes of interest identified by tBLASTx was tested by reciprocal BLAST against NCBI Genbank and followed by phylogenetic analyses. Amino acid alignments were made with MUSCLE [73]. RAxML (version 8.2.9) [74] was used to conduct a maximum-likelihood phylogenetic analysis. Fragments of the genes of interest were amplified from cDNA of *I*. *pulchra*, *M*. *stichopi*, and *N*. *vectensis* by PCR using gene-specific primers. PCR products were purified and cloned into a pGEM-T Easy vector (Promega, Madison, WI, USA) according to the manufacturer’s instructions, and the identity of inserts was confirmed by sequencing. Gene accession numbers of the gene sequences are listed in the S3 Table.

### Animal systems

Adult specimens of *I*. *pulchra* (Smith & Bush, 1991), *M*. *stichopi* Westblad, 1949, and *N*. *vectensis* Stephenson, 1935 were kept and handled as previously described [75–78].

### WMISH

Animals were manually collected, fixed, and processed for in situ hybridization as described [79,80]. Labeled antisense RNA probes were transcribed from linearized DNA using digoxigenin-11-UTP (Roche, Basel, Switzerland) or labeled with DNP (Mirus Bio, Madison, WI, USA) according to the manufacturer’s instructions. For *I*. *pulchra* and *M*. *stichopi*, colorimetric WMISH was performed according to the protocol outlined in [79]. For *N*. *vectensis*, we followed the protocol described by [81]. Double fluorescent in situ hybridization (FISH) was performed as the colorimetric WMISH with the following modifications: after the posthybridization steps, animals were incubated overnight with peroxidase-conjugated antibodies at 4 °C (anti-DIG-POD [Roche, Basel, Switzerland], 1:500 dilution, and anti-DNP [Perkin Elmer, Waltham, MA, USA], 1:200 dilution) followed by the amplification of the signal with fluorophore-conjugated tyramides (1:100 TSA reagent diluents [Perkin Elmer, Waltham, MA, USA] TSA Plus Cy3 or Cy5 Kit). Residual enzyme activity was inhibited via 45-minute incubation in 0.1% hydrogen peroxide in PTW followed by four PTW washes prior to addition and development of the second peroxidase-conjugated antibody [82].

### Whole-mount immunohistochemistry

Animals were collected manually, fixed in 4% paraformaldehyde in SW for 50 minutes, washed 3 times in PBT, and incubated in 4% sheep serum in PBT for 30 minutes. The animals were then incubated with commercially available primary antibodies (anti-RhAG [ab55911] rabbit polyclonal antibody, dilution 1:50 [Abcam, Cambridge, UK], anti-Na^+^/K^+^ ATPase a1 subunit rat monoclonal antibody, dilution 1:100 [Sigma-Aldrich, St. Louis, MO, USA], and anti-V-ATPase B1/2 [L-20] goat polyclonal antibody, dilution 1:50 [Santa Cruz Biotechnology, Dallas, TX, USA]) overnight at 4 °C, washed 5 times in PBT, and followed by incubation in 4% sheep serum in PBT for 30 minutes. Specimens were then incubated with a secondary antibody (anti-rabbit-AlexaFluor 555 [Invitrogen, Carlsbad, CA, USA] or anti-rat-AlexaFluor 555 and anti-goat-AlexaFluor 555) diluted 1:1,000 overnight at 4 °C followed by 10 washes in PBT. Nuclei were stained by incubation of animals in DAPI 1:1,000, and f-actin was stained by incubation in BODIPY-labeled phallacidin (5 U/ml) overnight.

### Inhibitor and HEA experiments

For excretion experiments, approximately 300 *I*. *pulchra* (the number varied slightly between the biological replica but was similar in the corresponding controls and treatments) and 10 *N*. *vectensis* were placed into glass vials with 2 ml UV-sterilized natural seawater (1:4 diluted with distilled water for *N*. *vectensis*) containing the appropriate inhibitor or ammonia concentration. Animals were given 10 minutes to adjust to the medium before the solution was exchanged with 2 ml of fresh medium with the same appropriate condition. For the inhibitor experiment, the medium was removed after 2 hours and stored at −80 °C for later measurements. Animals from the short-term HEA experiments were incubated for 2 hours, rinsed five times over 20–30 minutes, and incubated for another 2 hours in fresh medium without additional ammonia, after which the medium was removed and frozen at −80 °C. We tested different inhibitor concentrations that were used in previous studies in other invertebrates [10,12,14,19,27]. The concentrations of 5–15 μM concanamycin C for inhibiting V-ATPase A/B, 1–3 mM azetazolamide as an inhibitor of the CA, 1–5 mM quabain to inhibit the NKA, and 2–10 mM colchicine for inhibiting the microtubule network were selected, as no other effects like shrinking or obvious changes in morphology or behavior were observed. After the inhibitor incubations, the animals were washed several times and monitored in normal conditions for several days to ensure that the inhibitors did not cause any unspecific permanent effects. Concanamycin C was diluted in DMSO with a final concentration of 0.5% DMSO per sample, for which we used an appropriate control with 0.5% DMSO. For the HEA experiments, we enriched seawater with NH_4_Cl to the final ammonia concentrations of 50 μM, 100 μM, 200 μM, 500 μM, and 1 mM. We also measured the pH of both incubation mediums (HEA and control), and we found no difference. All experiments were independently repeated at least three times at different time points, and each repeat was divided into two samples. The values are provided in S6 Table.

### Determination of ammonia excretion

Ammonia concentrations were measured with an ammonia-sensitive electrode (Orion, Thermo Scientific, Waltham, MA, USA) according to [52]. Samples were diluted 1:4 with distilled water to prevent salt precipitation (900 μl sample + 2.7 ml water), and total ammonia was transformed into gaseous NH_3_ by adding 54 μl ionic strength adjusting solution (1.36 ml/l trisodiumcitrate dihydrate, 1 M NaOH). Because of the small ammonia concentrations, the electrode-filling solution was diluted to 10% with distilled water, as suggested in the electrode manual. In control conditions, we determined an average excretion of 44 pmol per adult animal per hour, although the excretion varied between different biological replicates from different generations (minimum 32 pmol/animal/hour, maximum 52 pmol/animal/hour), possibly because of slightly fluctuating conditions during long-term animal rearing. Solutions with defined concentrations of NH_4_Cl for the standard curves were made together with the solutions used in the experiments and stored in a similar way at −80 °C. The differences in excretion rates were tested for significance with an unpaired, 2-tailed *t* test with unequal variance, and a *p*-value < 0.02 was seen as significant. Boxplots were created with “R.”

### Quantitative gene expression

In total, 100 treated *I*. *pulchra* and 5 *N*. *vectensis* were collected after 7 days of incubation in HEA conditions (1 mM NH_4_Cl) and tested for quantitative gene expression using the BIORAD CFX96 (Bio-Rad, Hercules, CA, USA) Real-time PCR detection system. ddCt values were calculated between treated and control animals and converted to fold differences. All experiments were repeated three to five times with different specimens (three biological replicates for *I*. *pulchra* and five biological replicates for *N*. *vectensis*), and two to four technical replicates were tested for each biological replicate (four biological replicates for *I*. *pulchra* and three biological replicates for *N*. *vectensis*). Fold changes were calculated using polyubiquitin, actin, and 18S as references for *I*. *pulchra* and *ATPsynthase* and *EF1b* as references for *N*. *vectensis* [83], and a threshold of 2-fold difference was chosen as a significant change. The Ct values are provided in S4 Table, and the primer sequences used are provided in S5 Table.

### Western blot

Whole-animal extracts (50 *I*. *pulchra* adults and 5 *N*. *vectensis* juveniles) were fractionated by SDS-PAGE, loaded on Mini-PROTEAN TGX Stain-Free Precast Gels (Bio-Rad, Hercules, CA, USA), and transferred to a nitrocellulose membrane using a transfer apparatus according to the manufacturer’s protocols (Bio-Rad, Hercules, CA, USA). After incubation with 5% nonfat milk in TBST (10 mM Tris, [pH 8.0], 150 mM NaCl, 0.5% Tween 20) for 60 minutes, the membrane was washed once with TBST and incubated with antibodies against Rhesus (1:1,000) and NKA (1:500) at 4 °C for 12 hours. Membranes were washed three times for 10 minutes and incubated with a 1:5,000 dilution of horseradish peroxidase–conjugated anti-mouse or anti-rabbit antibodies for 2 hours. Blots were washed with TBST three times and developed with the ECL system (Amersham Biosciences, Little Chalfont, UK) according to the manufacturer’s protocols.

### Documentation

Colorimetric WMISH specimens were imaged with a Zeiss AxioCam HRc mounted on a Zeiss Axioscope A1 equipped with Nomarski optics and processed through Photoshop CS6 (Adobe, San Jose, CA, USA). Fluorescent-labeled specimens were analyzed with a Leica SP5 confocal laser microscope (Leica Microsystems, Wetzlar, Germany) and processed by the Fiji software version 2.0.0-rc-42/1.50d [84]. Figure plates were arranged with Illustrator CS6 (Adobe, San Jose, CA, USA). Data were deposited in the Dryad repository: https://doi.org/10.5061/dryad.bq068jr [72].

## Supporting information

S1 Fig Excretion-related gene complement in different animal lineages and outgroups and animals in this study. (a) Transcriptome and genome mining of excretion-related gene repertoire in *I*. *pulchra*, *H*. *miamia*, *C*. *macropyga*, *D*. *longitubus*, *D*. *gymnopharyngeus*, *E*. *macrobursalium*, and *C*. *submaculatum* as representatives of Acoela; *Sterreria* sp., *Ascoparia* sp., *M*. *stichopi*, and *N*. *westbladi* as representatives of Nemertodermatida; *X*. *bocki* and *X*. *profunda* as representatives of *Xenoturbella*; *N*. *vectensis* as a representative of cnidarians; *T*. *adhaerens* as a representative of placozoans; *A*. *queenslandica* as a representative of sponges; and the deuterostomes *H*. *sapiens*, *S*. *kowalevskii*, *S*. *purpuratus*, *X*. *laevis*, and *B*. *lanceolatum* and protostomes *C*. *teleta*, *C*. *gigas*, *L*. *gigantea*, *S*. *mediterranea*, *T*. *castaneum*, *C*. *elegans*, and *D*. *melanogaster* as representatives of Nephrozoa. Data are based on this study unless stated otherwise. (b) Pictures of the acoelomorph representatives *I*. *pulchra* (scale bar = 50 μm) and *M*. *stichopi* (scale bar = 100 μm) and the cnidarian representative *N*. *vectensis* (scale bar = 2 mm). Animal illustrations are taken from phylopic.org.
(TIF)

S2 Fig Orthology analysis. Putative orthologous sequences of genes of interest were identified by tBLASTx search against the transcriptome (SRR2681926) of *I*. *pulchra*, the transcriptome (SRR2681155) and draft genome of *M*. *stichopi*, and the genome of *N*. *vectensis* (http://genome.jgi.doe.gov). Additional transcriptomes of Xenacoelomorpha species investigated were as follows: *C*. *submaculatum* (Acoela) (SRX1534054), *C*. *macropyga* (Acoela) (SRX1343815), *D*. *gymnopharyngeus* (Acoela) (SRX1534055), *D*. *longitubus* (Acoela) (SRX1534056), *E*. *macrobursalium* (Acoela) (SRX1534057), *H*. *miamia* (Acoela) (PRJNA241459), *Ascoparia* sp. (Nemertodermatida) (SRX1343822), *N*. *westbladi* (Nemertodermatida) (SRX1343819), *Sterreria* sp. (Nemertodermatida) (SRX1343821), *X*. *bocki* (Xenoturbella) (SRX1343818), and *X*. *profunda* (Xenoturbella) (SRP064117). Bayesian phylogenetic analysis is supporting orthology for genes investigated in this study. Red color refers to Xenacoelomorpha taxa, and blue color refers to *N*. *vectensis*. Bootstrap values are shown when equal or above 20%. Branches crossed by a double slash were shortened to make figures’ plates more compact. Names of genes or proteins, if available, follow the name of organism(s); otherwise, the accession number is written. Asterisks indicate genes with a spatial expression by WMISH. WMISH, whole-mount in situ hybridization.
(PDF)

S3 Fig WMISH of ultrafiltration and tubule and duct–related genes in *I*. *pulchra* and *M*. *stichopi*. Expression of genes encoding the slit diaphragm components related to ultrafiltration *nephrin*/*kirre*, *cd2ap*, *zo1*, and *stomatin*/*podocin*, and the SLCs related to excrete modification *slc1*, *slc5*, and *slc13* (proximal tubule) and *slc4*, *slc8*, *slc9*, *slc12*, and *slc26* (distal tubule and duct) in *I*. *pulchra* and *M*. *stichopi*. The inset in panel A3 shows a different focal plane of the indicated domain and in panel E3 shows a different focal plane of the animal. The columns next to *M*. *stichopi* panels show higher magnifications of the indicated domains. Anterior is to the left. BG indicates background staining. BG, background; *cd2ap*, CD2-associated protein; mo, mouth; mg, male gonopore; SLC, solute carrier transporter; st, statocyst; WMISH, whole-mount in situ hybridization; *zo1*, zonula occludens 1.
(TIF)

S4 Fig Double fluorescent WMISH of excretion-related components and molecular markers of digestive, nervous, and reproductive systems in *I*. *pulchra* and *M*. *stichopi*. Coexpression analysis of *nka* with the reproductive system markers *piwi* and *vasa* (A), *hcn* with the nervous system marker chaT (B), *v-ATPase* with the digestive marker *plastin* in *I*. *pulchra* (C), *slc4b* with the nervous system marker tyrosinated tubulin (D), and *v-ATPase* with the digestive marker *plastin* in *M*. *stichopi* (E). Every picture is a full projection of merged confocal stacks. Nuclei are stained blue with DAPI. Anterior is to the left. Scale bars are 50 μm for *I*. *pulchra* and 100 μm for *M*. *stichopi*. br, brain; chaT, choline acetyltransferase; DAPI, 4',6-diamidino-2-phenylindole; ds, digestive syncytium; gwc, gut-wrapping cell; *hcn*, K^+^[NH4^+^] channel; nc, nerve cord; *nka*, Na^+^/K^+^[NH4^+^] ATPase; *slc*, solute carrier transporter; te, testis; v-ATPase, vacuolar H^+^-ATPase proton pump; WMISH, whole-mount in situ hybridization.
(TIF)

S5 Fig WMISH of the ammonia excretion–related genes *rh*, *v-ATPase B*, *nka*, *ca*, *hcn*, *amts*, and *aquaporins* in *I*. *pulchra* and *M*. *stichopi*. The insets in panels B6, C1, and C3 show different focal planes of the animals. The columns next to *M*. *stichopi* panels show higher magnifications of the indicated domains, except of G2‘, which shows a different focal plane of the animal. The inset in panel G5‘ shows a side view of the animal. Anterior is to the left. *amt*, ammonia transporter; *ca*, carbonic anhydrase; mo, mouth; *nka*, Na^+^/K^+^[NH4^+^] ATPase; *rh*, Rhesus glycoprotein; *v-ATPase*, vacuolar H^+^-ATPase proton pump; WMISH, whole-mount in situ hybridization.
(TIF)

S6 Fig Western blot of Rhesus in *I*. *pulchra* and Rhesus and NKA in *N*. *vectensis*. Below each blot, the sequence alignment of the endogenous protein and the antigen is provided, highlighted in pink. NKA, Na^+^/K^+^[NH4^+^] ATPase.
(PDF)

S7 Fig Double fluorescent WMISH of *v-ATPase* with *nka* and *v-ATPase* with *rhesus* in *M*. *stichopi*. Every picture is a full projection of merged confocal stacks. Nuclei are stained blue with DAPI. Anterior is to the left. DAPI, 4',6-diamidino-2-phenylindole; dlr, distal lateral row; ds, digestive syncytium; gwc, gut-wrapping cell; *nka*, Na^+^/K^+^[NH4^+^] ATPase; *v-ATPase*, vacuolar H^+^-ATPase proton pump; WMISH, whole-mount in situ hybridization.
(TIF)

S8 Fig WMISH of ammonia transporters in *N*. *vectensis*. Gene expression of *amt2/3a*, *amt2/3b*, *amt2/3c*, *amt2/3d*, and *amt1/4a* in juvenile polyps. Anterior is to the left. *amt*, ammonia transporter; WMISH, whole-mount in situ hybridization.
(TIF)

S9 Fig WMISH and protein localization of NKA in *N*. *vectensis*. Gene expression of *nka a* and *nka b* in juvenile polyps. Anterior is to the left. Protein localization of NKA in *N*. *vectensis* juvenile polyps. The muscle filaments are labeled green with phalloidin, and the nervous system is stained cyan with tyrosinated tubulin. Every picture is a full projection of merged confocal stacks. Nuclei are stained blue with DAPI. The regions shown are indicated with dashed boxes in the illustrated animal. DAPI, 4',6-diamidino-2-phenylindole; ebw, endodermal body wall; mes, mesenteries; n, neuron; NKA, Na^+^/K^+^[NH4^+^] ATPase; sf, septal filament; WMISH, whole-mount in situ hybridization.
(TIF)

S10 Fig Phylogenetic tree showing the relationship of animal groups in which the role of gut in excretion has been demonstrated or proposed. (TIF)

S1 Table Compilation of excretion-related gene expression/role data for metazoan and nonmetazoan taxa for genes investigated in this study, when data are available. Data are based on this study unless stated otherwise. Question marks represent missing data.
(PDF)

S2 Table *N*. *vectensis* excretion in different pH. (PDF)

S3 Table Accession numbers and transcript numbers from Xenacoelomorph transcriptomes used in S1 and S2 Figs. (PDF)

S4 Table QPCR raw data. QPCR, quantitative PCR.
(PDF)

S5 Table QPCR primers used in this study. QPCR, quantitative PCR.
(PDF)

S6 Table Ammonia excretion measurements raw data. (PDF)

## Abbreviations

AMT: ammonia transporter
CA: carbonic anhydrase
cd2ap: CD2-associated protein
cu: cuticle
HCN: K^+^[NH4^+^] channel
HEA: high environmental ammonia
NKA: Na^+^/K^+^[NH4^+^] ATPase
qPCR: quantitative PCR
Rh: Rhesus glycoprotein
SLC: solute carrier transporter
v-ATPase: vacuolar H^+^-ATPase proton pump
WMISH: whole-mount in situ hybridization
zo1: zonula occludens 1
