# Supplementary figures and images for: Active mode of excretion across digestive tissues predates the origin of excretory organs
Source: PLoS Biol. 2019 Jul 29;17(7):e3000408. doi: 10.1371/journal.pbio.3000408 (PMC6687202; doi:10.1371/journal.pbio.3000408)

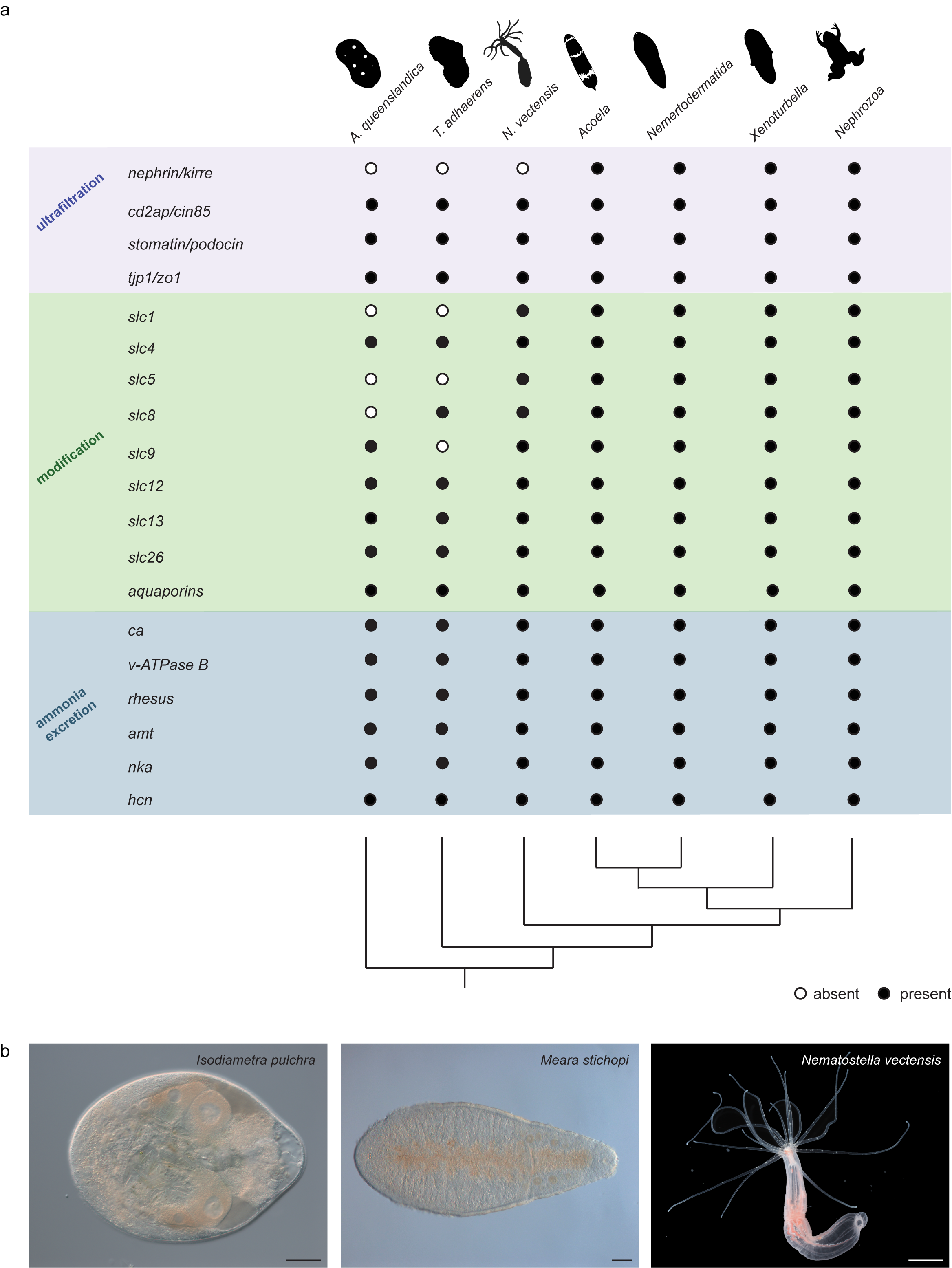

Supplement: S1 Fig — (a) Transcriptome and genome mining of excretion-related gene repertoire in I. pulchra, H. miamia, C. macropyga, D. longitubus, D. gymnopharyngeus, E. macrobursalium, and C. submaculatum as representatives of Acoela; Sterreria sp., Ascoparia sp., M. stichopi, and N. westbladi as representatives of Nemertodermatida; X. bocki and X. profunda as representatives of Xenoturbella; N. vectensis as a representative of cnidarians; T. adhaerens as a representative of placozoans; A. queenslandica as a representative of sponges; and the deuterostomes H. sapiens, S. kowalevskii, S. purpuratus, X. laevis, and B. lanceolatum and protostomes C. teleta, C. gigas, L. gigantea, S. mediterranea, T. castaneum, C. elegans, and D. melanogaster as representatives of Nephrozoa. Data are based on this study unless stated otherwise. (b) Pictures of the acoelomorph representatives I. pulchra (scale bar = 50 μm) and M. stichopi (scale bar = 100 μm) and the cnidarian representative N. vectensis (scale bar = 2 mm). Animal illustrations are taken from phylopic.org. (TIF) [file pbio.3000408.s001.tif]

**B**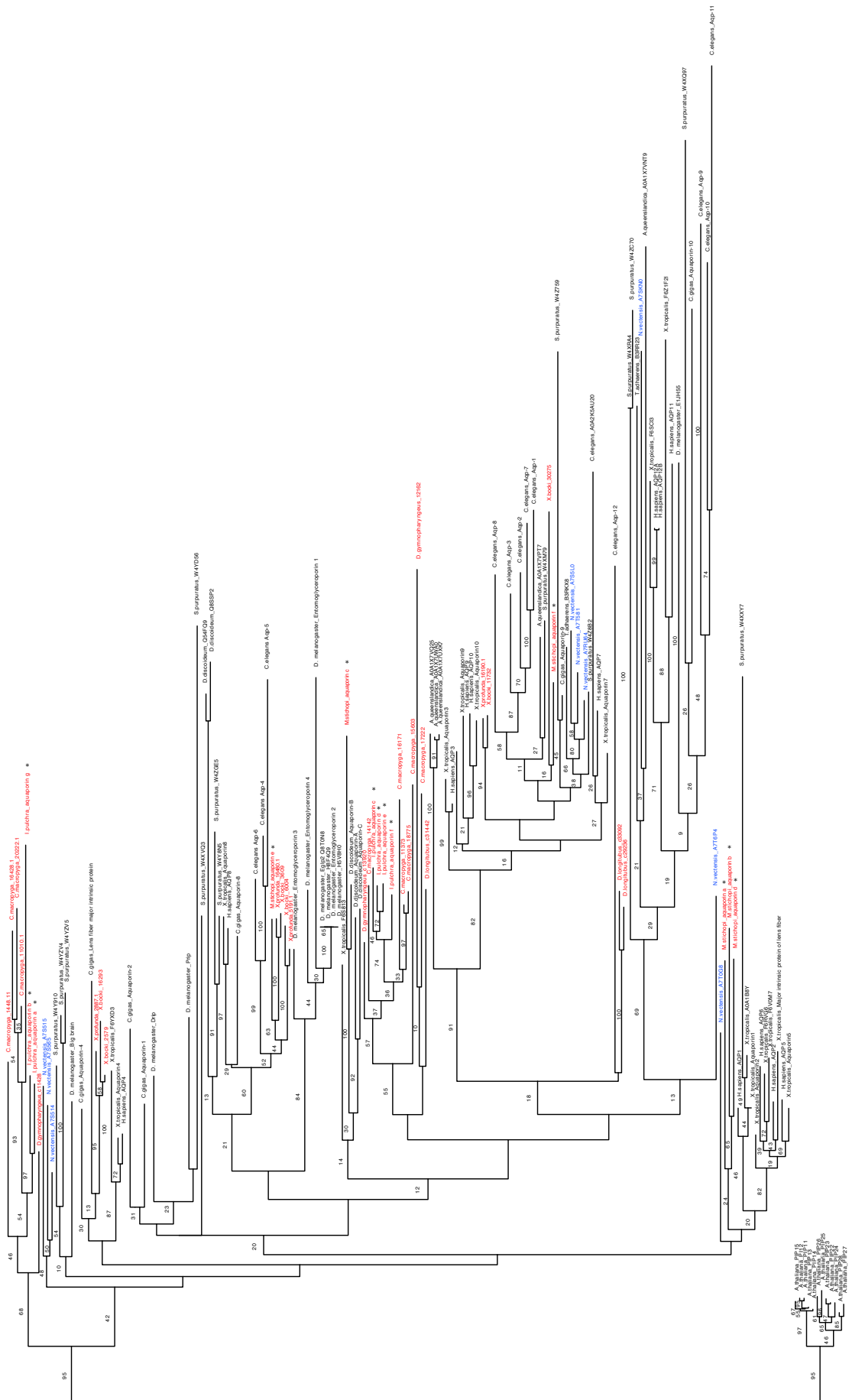

C

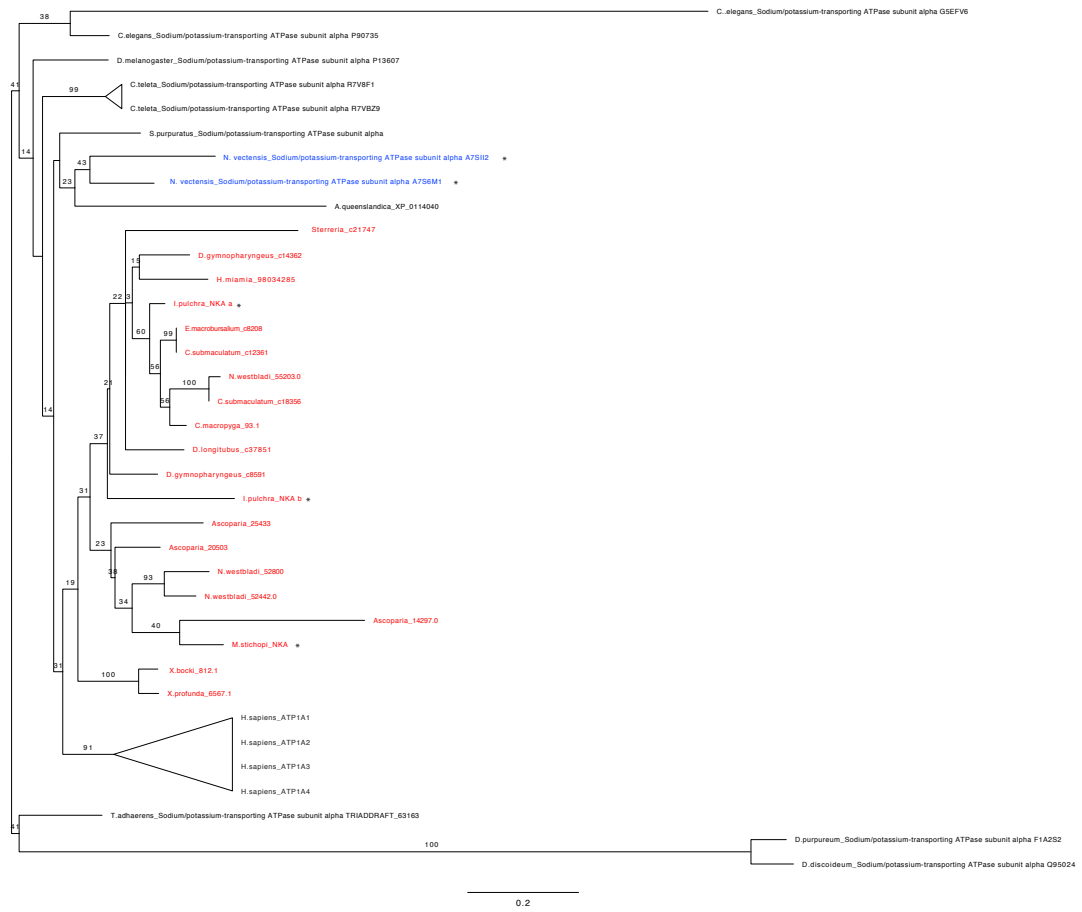

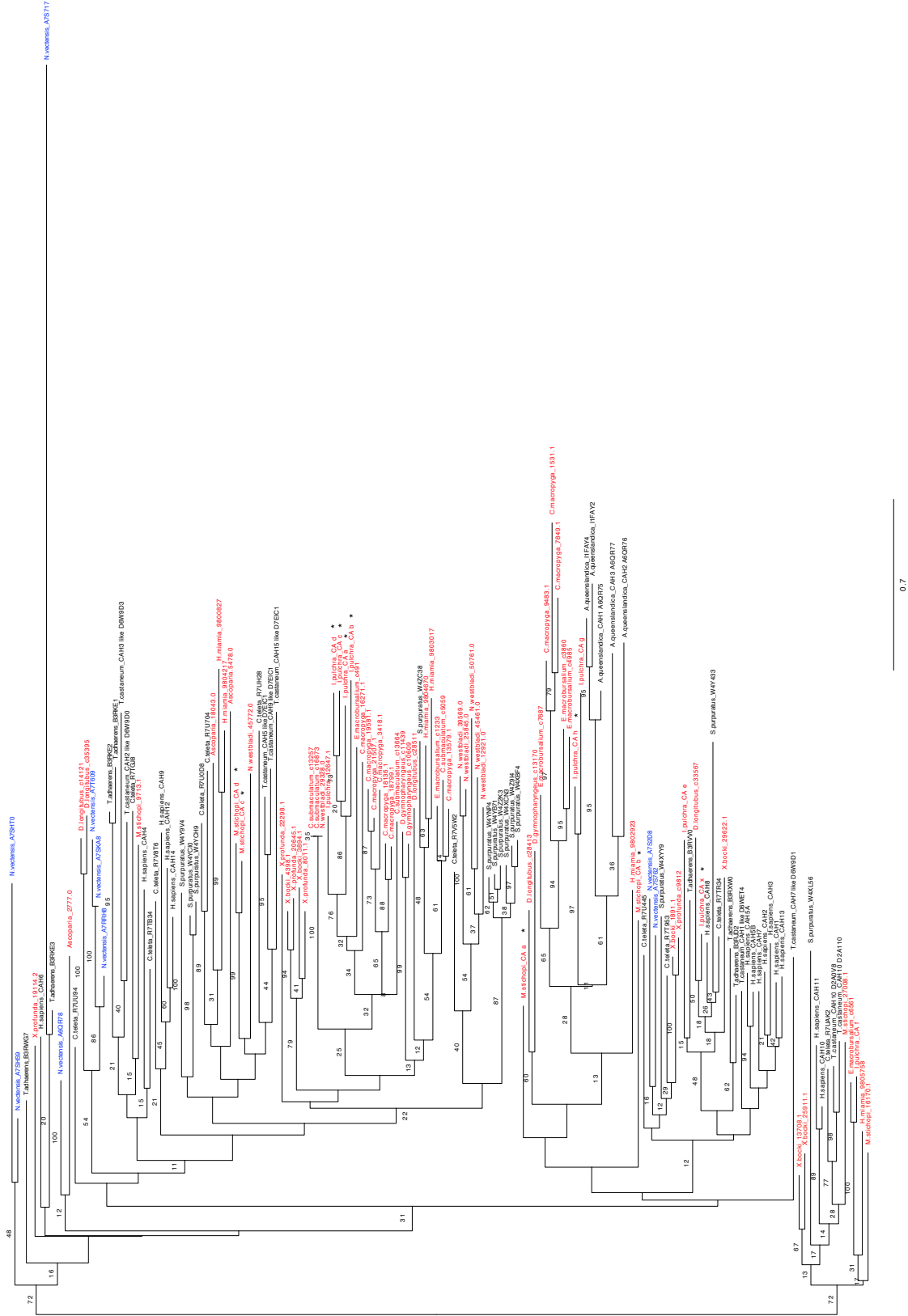

E

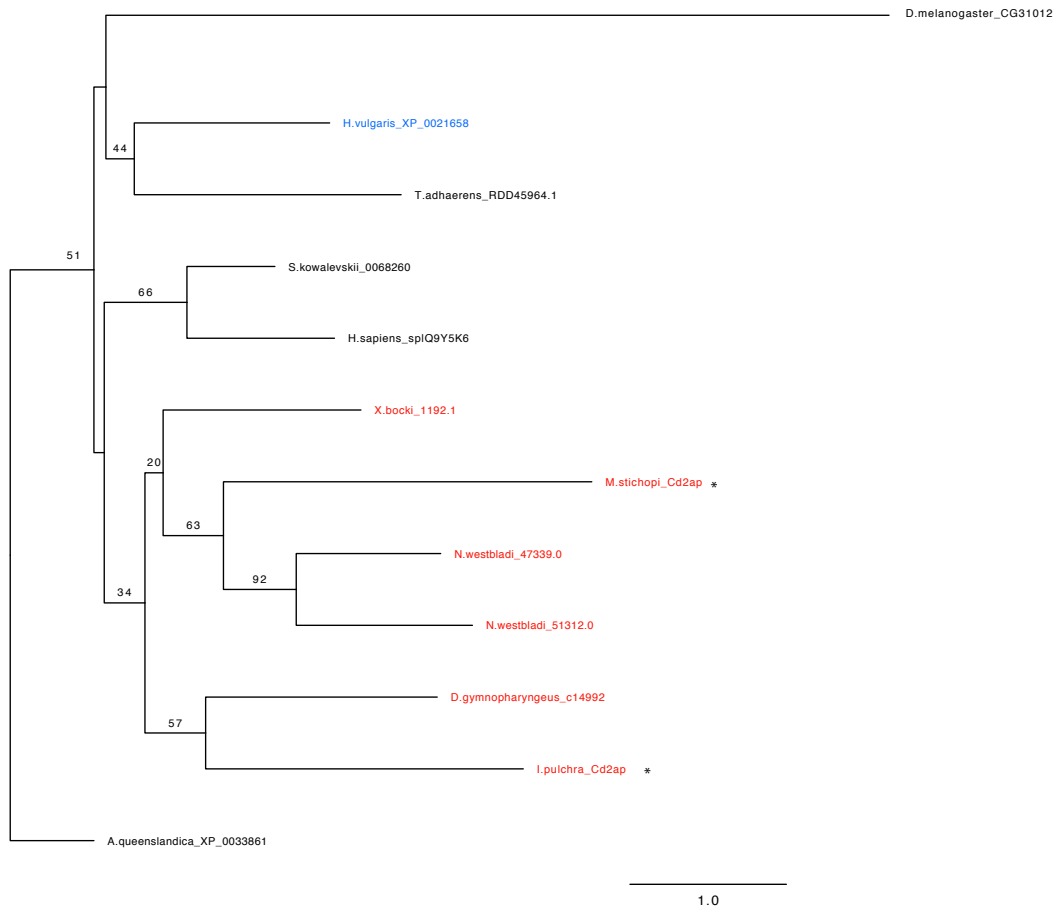

F

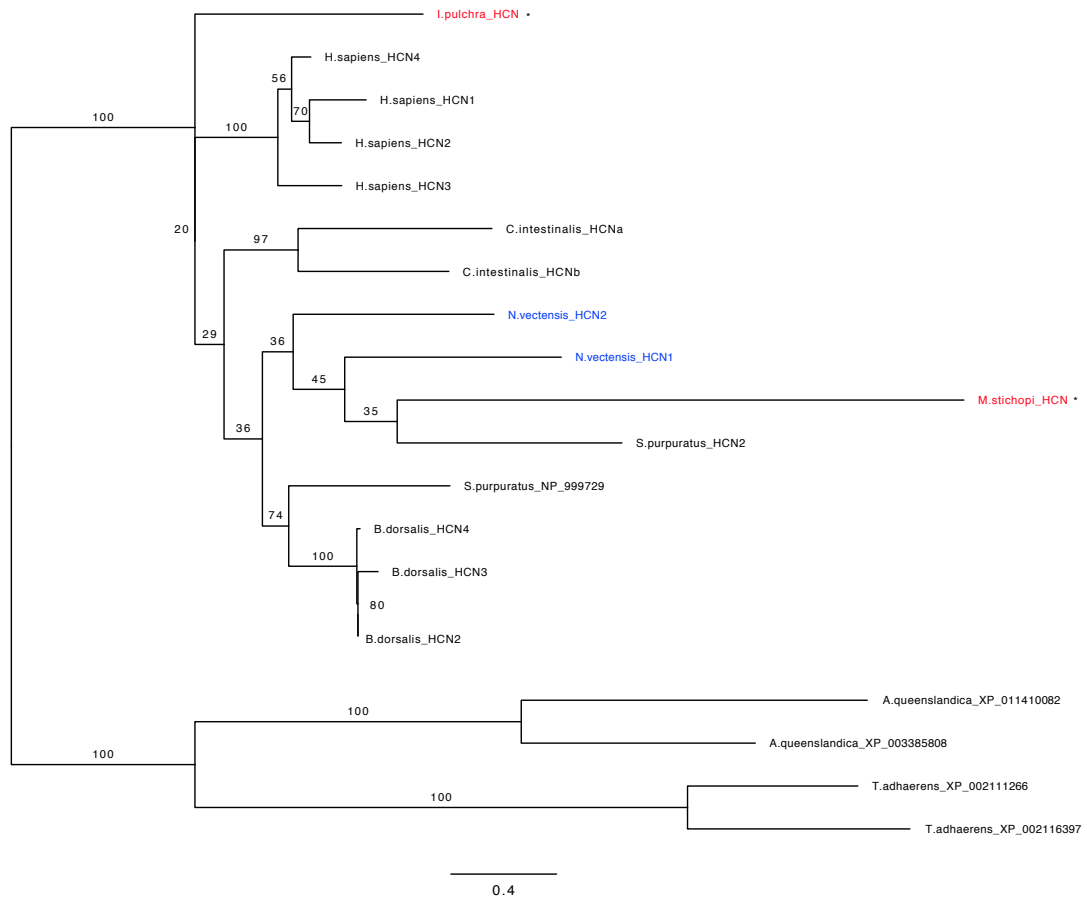

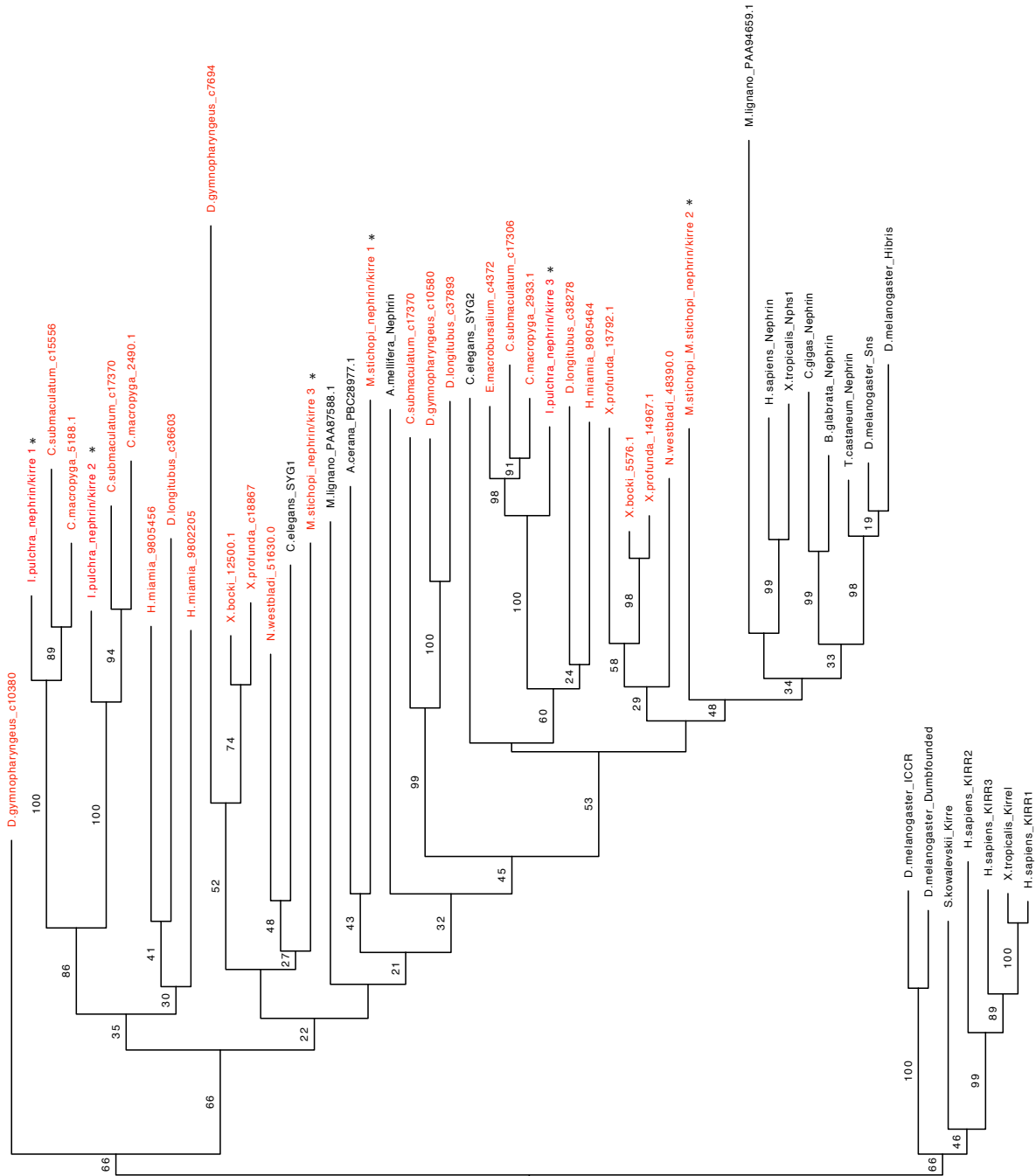

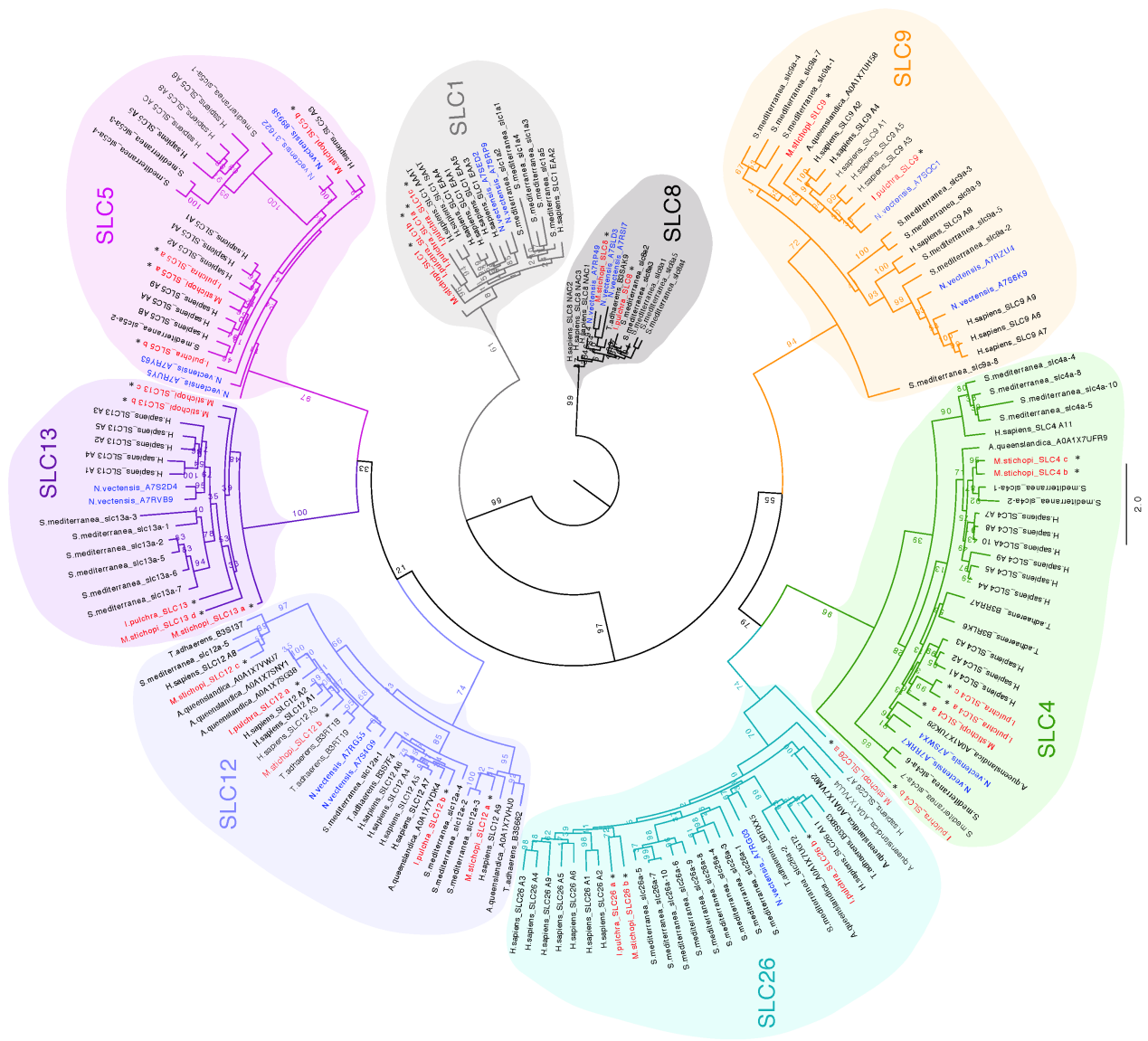

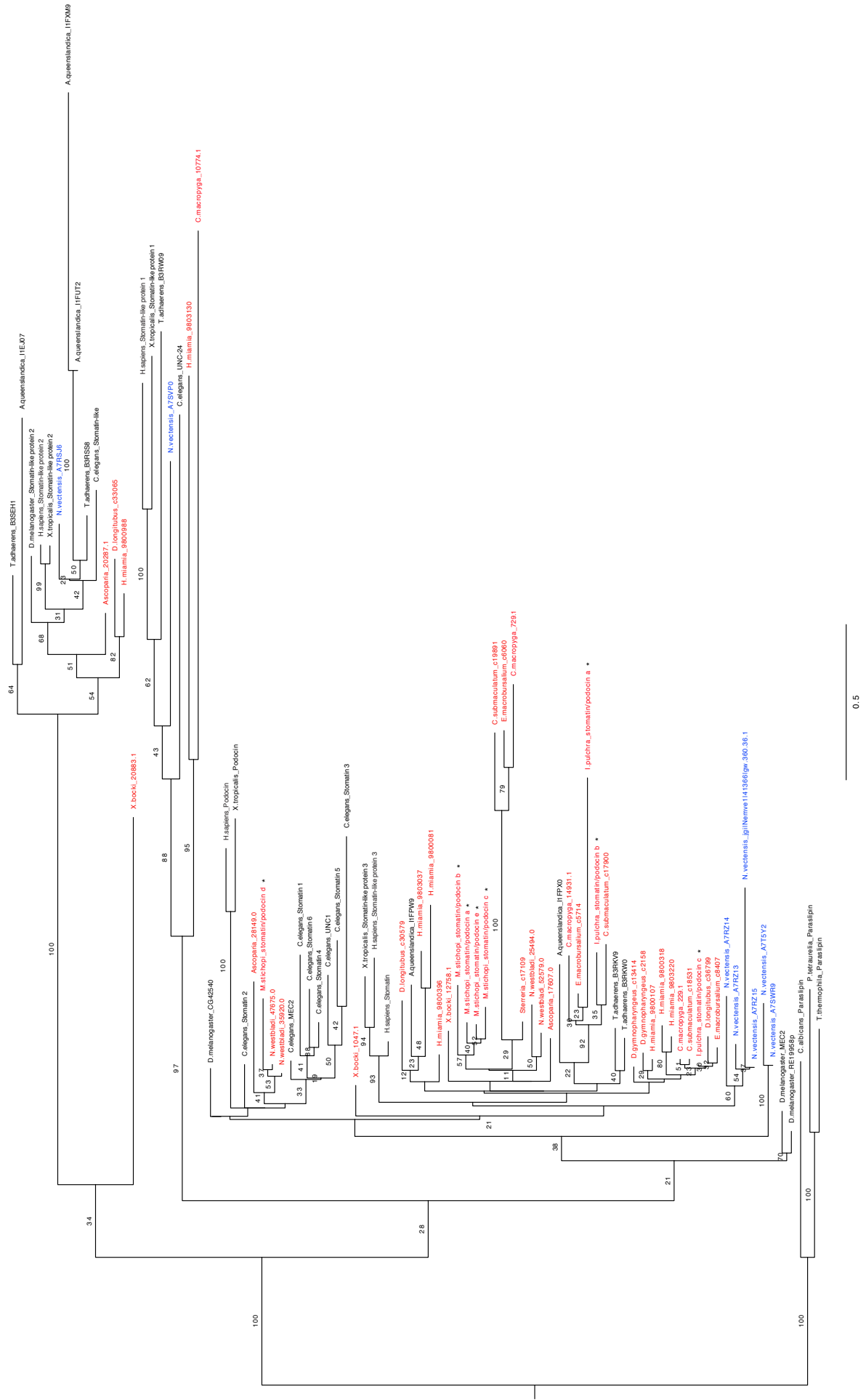

J

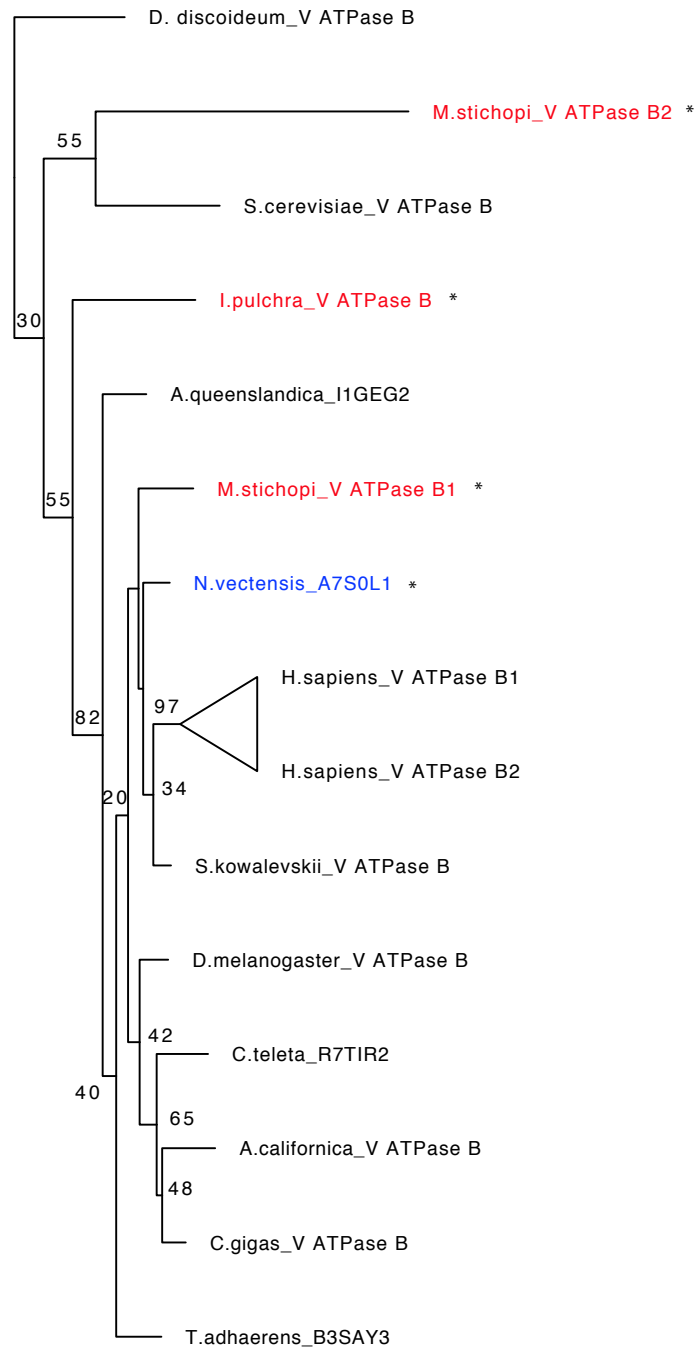

0.4

K

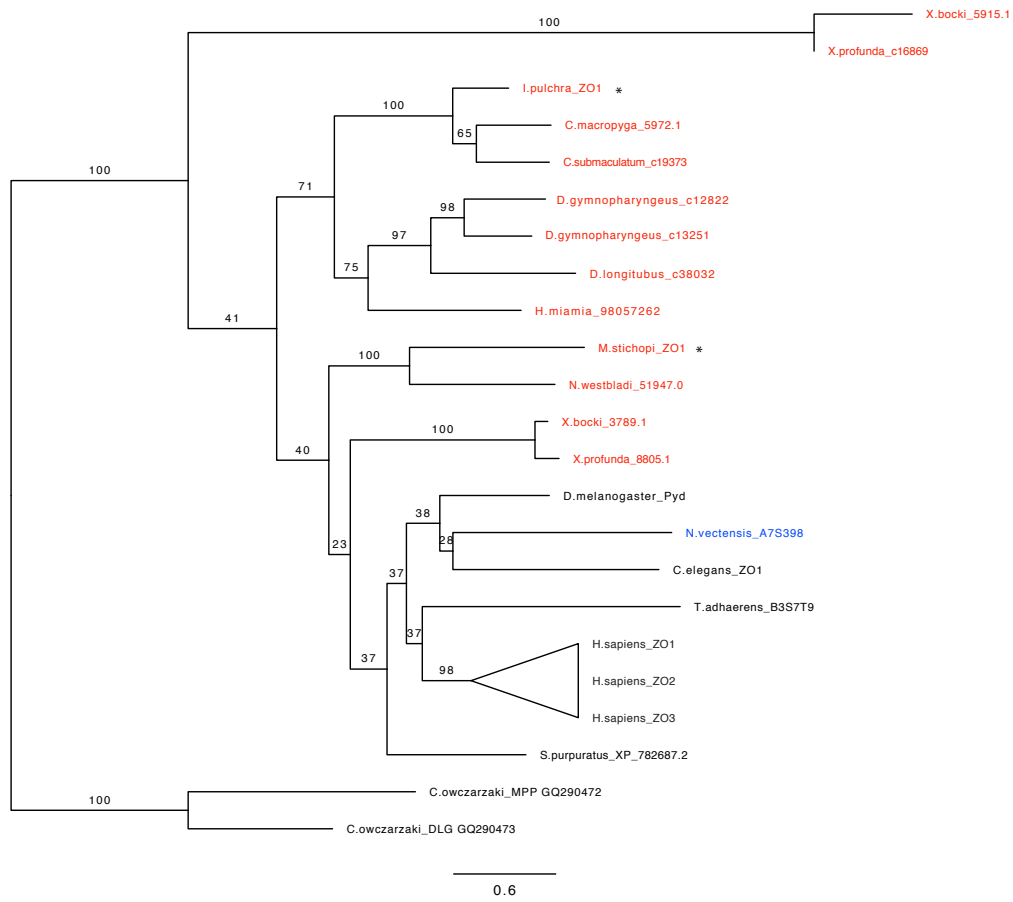

Supplement: S2 Fig — Putative orthologous sequences of genes of interest were identified by tBLASTx search against the transcriptome (SRR2681926) of I. pulchra, the transcriptome (SRR2681155) and draft genome of M. stichopi, and the genome of N. vectensis (http://genome.jgi.doe.gov). Additional transcriptomes of Xenacoelomorpha species investigated were as follows: C. submaculatum (Acoela) (SRX1534054), C. macropyga (Acoela) (SRX1343815), D. gymnopharyngeus (Acoela) (SRX1534055), D. longitubus (Acoela) (SRX1534056), E. macrobursalium (Acoela) (SRX1534057), H. miamia (Acoela) (PRJNA241459), Ascoparia sp. (Nemertodermatida) (SRX1343822), N. westbladi (Nemertodermatida) (SRX1343819), Sterreria sp. (Nemertodermatida) (SRX1343821), X. bocki (Xenoturbella) (SRX1343818), and X. profunda (Xenoturbella) (SRP064117). Bayesian phylogenetic analysis is supporting orthology for genes investigated in this study. Red color refers to Xenacoelomorpha taxa, and blue color refers to N. vectensis. Bootstrap values are shown when equal or above 20%. Branches crossed by a double slash were shortened to make figures’ plates more compact. Names of genes or proteins, if available, follow the name of organism(s); otherwise, the accession number is written. Asterisks indicate genes with a spatial expression by WMISH. WMISH, whole-mount in situ hybridization. (PDF) [file pbio.3000408.s002.pdf]

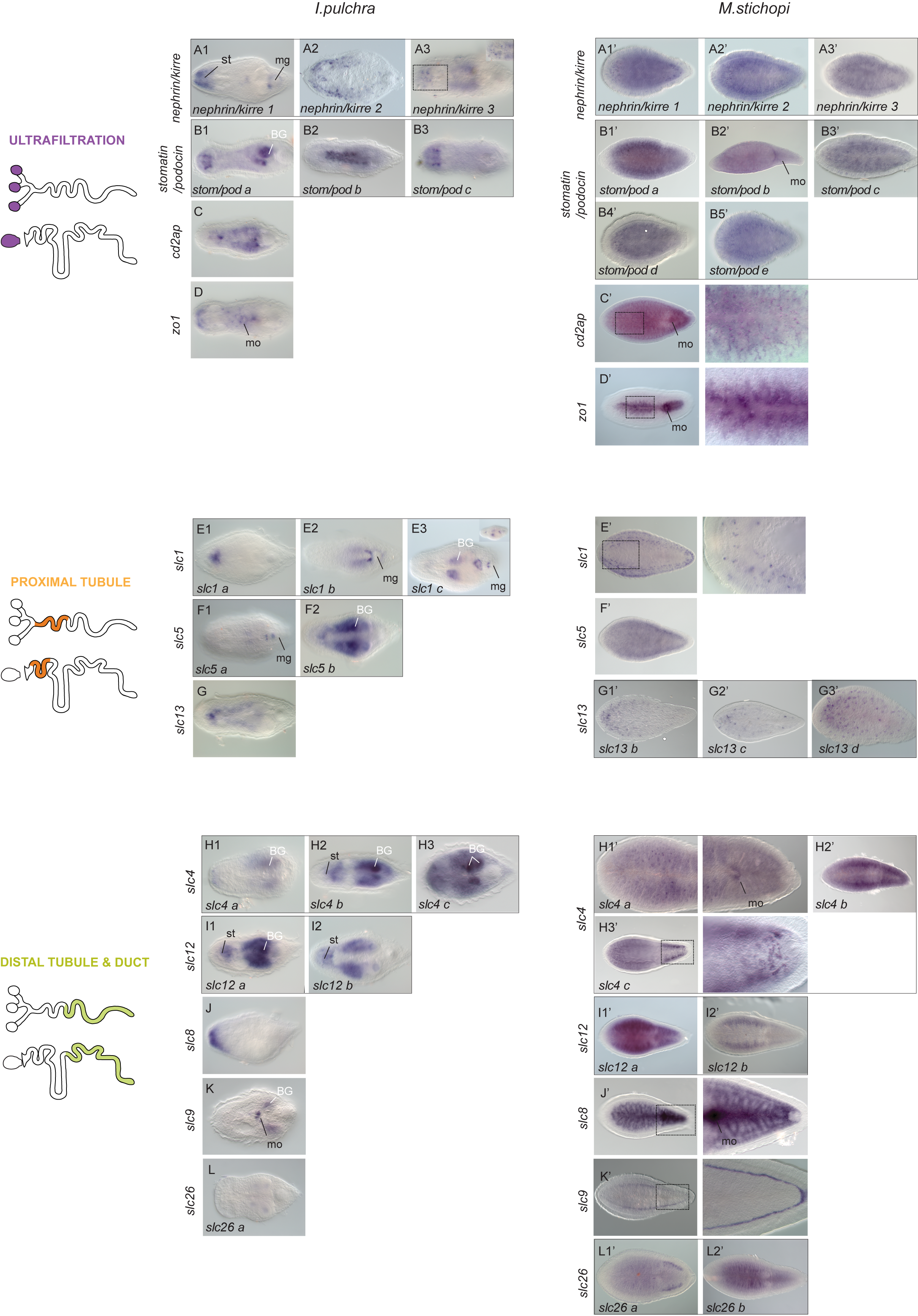

Supplement: S3 Fig — Expression of genes encoding the slit diaphragm components related to ultrafiltration nephrin/kirre, cd2ap, zo1, and stomatin/podocin, and the SLCs related to excrete modification slc1, slc5, and slc13 (proximal tubule) and slc4, slc8, slc9, slc12, and slc26 (distal tubule and duct) in I. pulchra and M. stichopi. The inset in panel A3 shows a different focal plane of the indicated domain and in panel E3 shows a different focal plane of the animal. The columns next to M. stichopi panels show higher magnifications of the indicated domains. Anterior is to the left. BG indicates background staining. BG, background; cd2ap, CD2-associated protein; mo, mouth; mg, male gonopore; SLC, solute carrier transporter; st, statocyst; WMISH, whole-mount in situ hybridization; zo1, zonula occludens 1. (TIF) [file pbio.3000408.s003.tif]

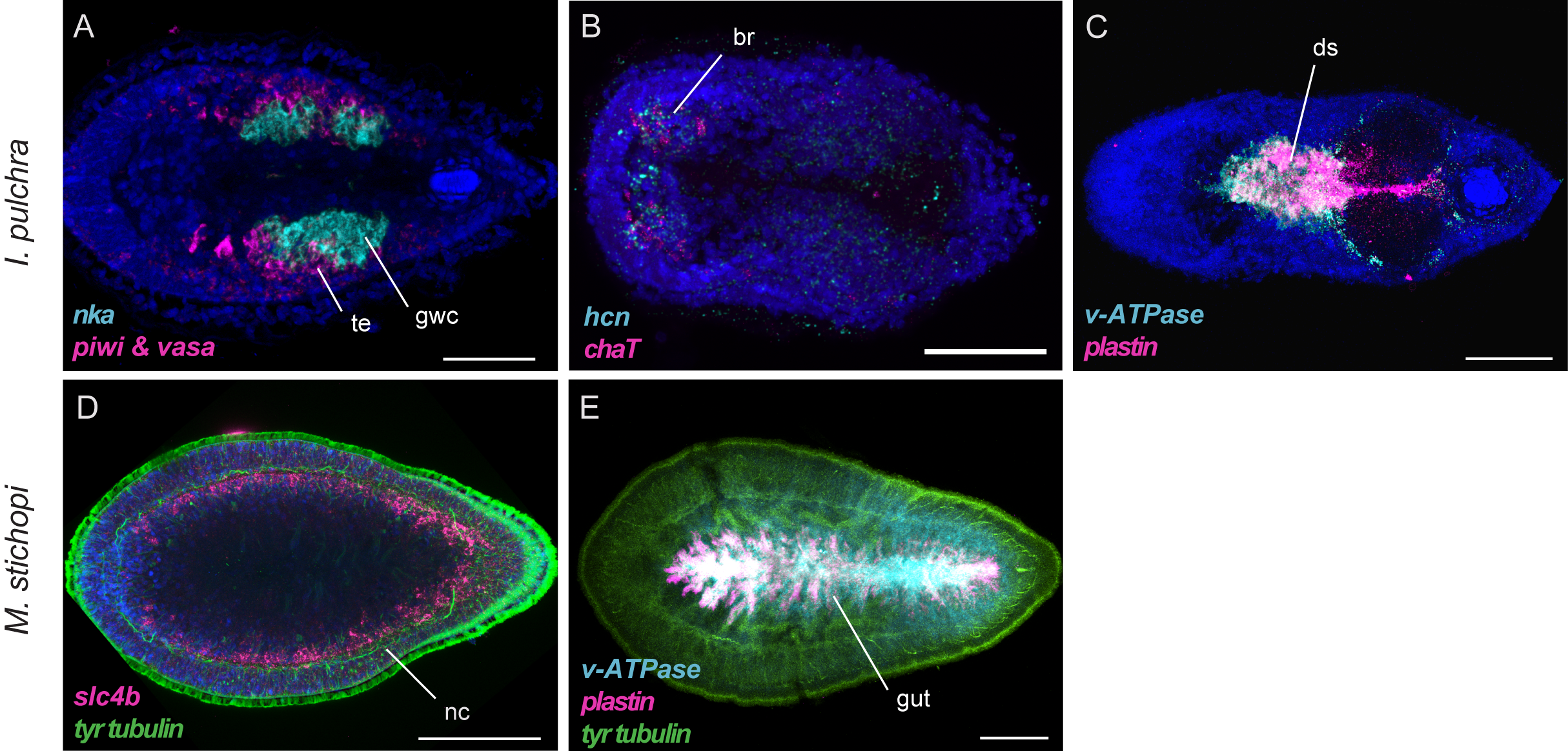

Supplement: S4 Fig — Coexpression analysis of nka with the reproductive system markers piwi and vasa (A), hcn with the nervous system marker chaT (B), v-ATPase with the digestive marker plastin in I. pulchra (C), slc4b with the nervous system marker tyrosinated tubulin (D), and v-ATPase with the digestive marker plastin in M. stichopi (E). Every picture is a full projection of merged confocal stacks. Nuclei are stained blue with DAPI. Anterior is to the left. Scale bars are 50 μm for I. pulchra and 100 μm for M. stichopi. br, brain; chaT, choline acetyltransferase; DAPI, 4',6-diamidino-2-phenylindole; ds, digestive syncytium; gwc, gut-wrapping cell; hcn, K+[NH4+] channel; nc, nerve cord; nka, Na+/K+[NH4+] ATPase; slc, solute carrier transporter; te, testis; v-ATPase, vacuolar H+-ATPase proton pump; WMISH, whole-mount in situ hybridization. (TIF) [file pbio.3000408.s004.tif]

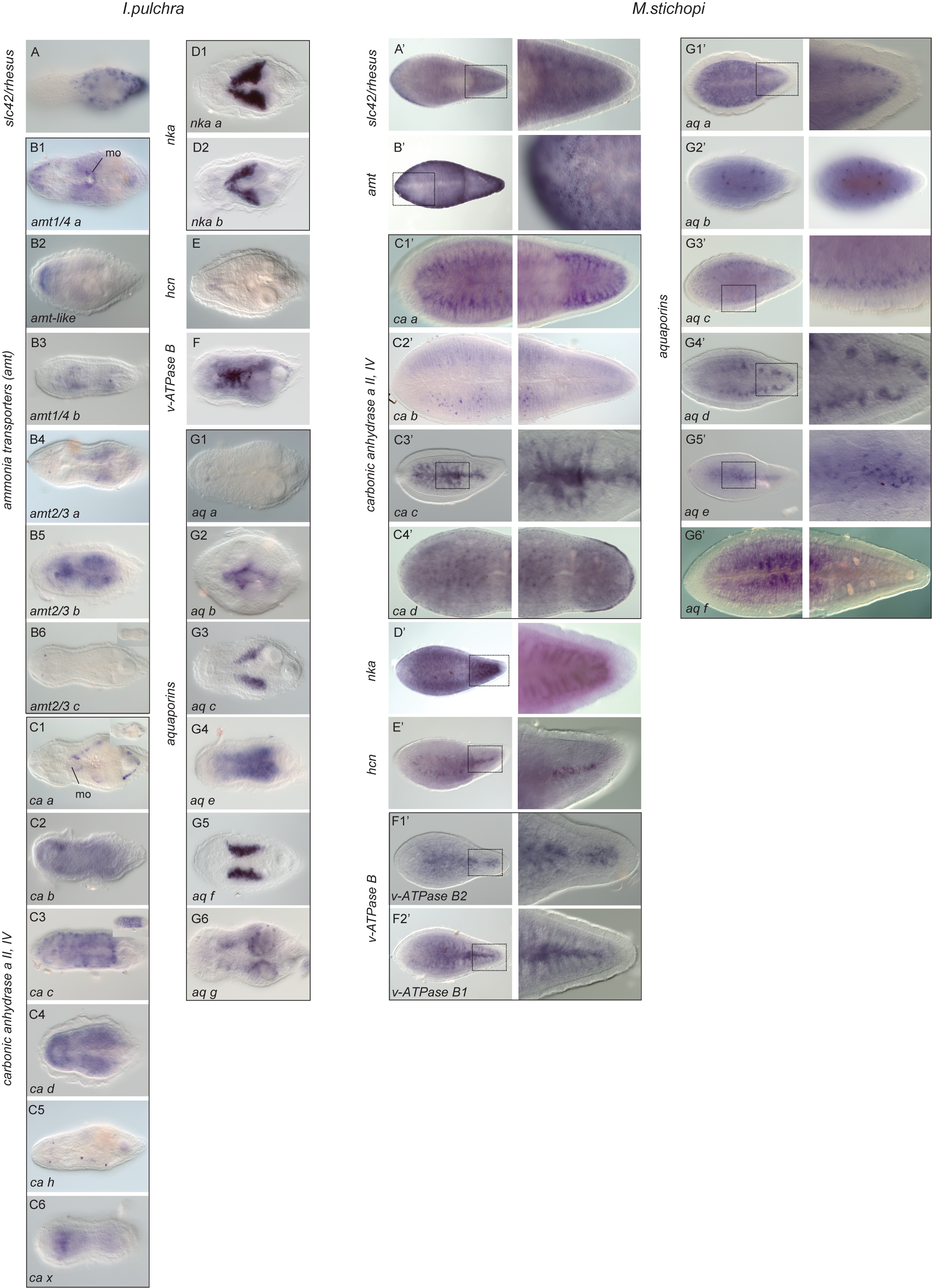

Supplement: S5 Fig — The insets in panels B6, C1, and C3 show different focal planes of the animals. The columns next to M. stichopi panels show higher magnifications of the indicated domains, except of G2‘, which shows a different focal plane of the animal. The inset in panel G5‘ shows a side view of the animal. Anterior is to the left. amt, ammonia transporter; ca, carbonic anhydrase; mo, mouth; nka, Na+/K+[NH4+] ATPase; rh, Rhesus glycoprotein; v-ATPase, vacuolar H+-ATPase proton pump; WMISH, whole-mount in situ hybridization. (TIF) [file pbio.3000408.s005.tif]

## Rhesus

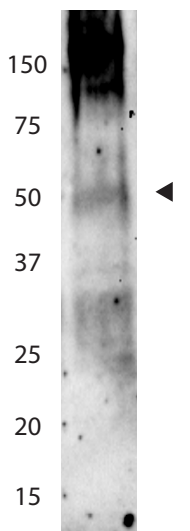[illegible][illegible][illegible][illegible]

## Rhesus

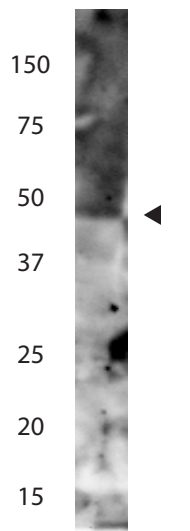

## NKA

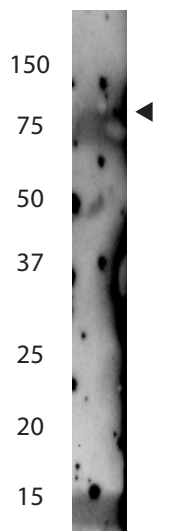[illegible]

Supplement: S6 Fig — Below each blot, the sequence alignment of the endogenous protein and the antigen is provided, highlighted in pink. NKA, Na+/K+[NH4+] ATPase. (PDF) [file pbio.3000408.s006.pdf]

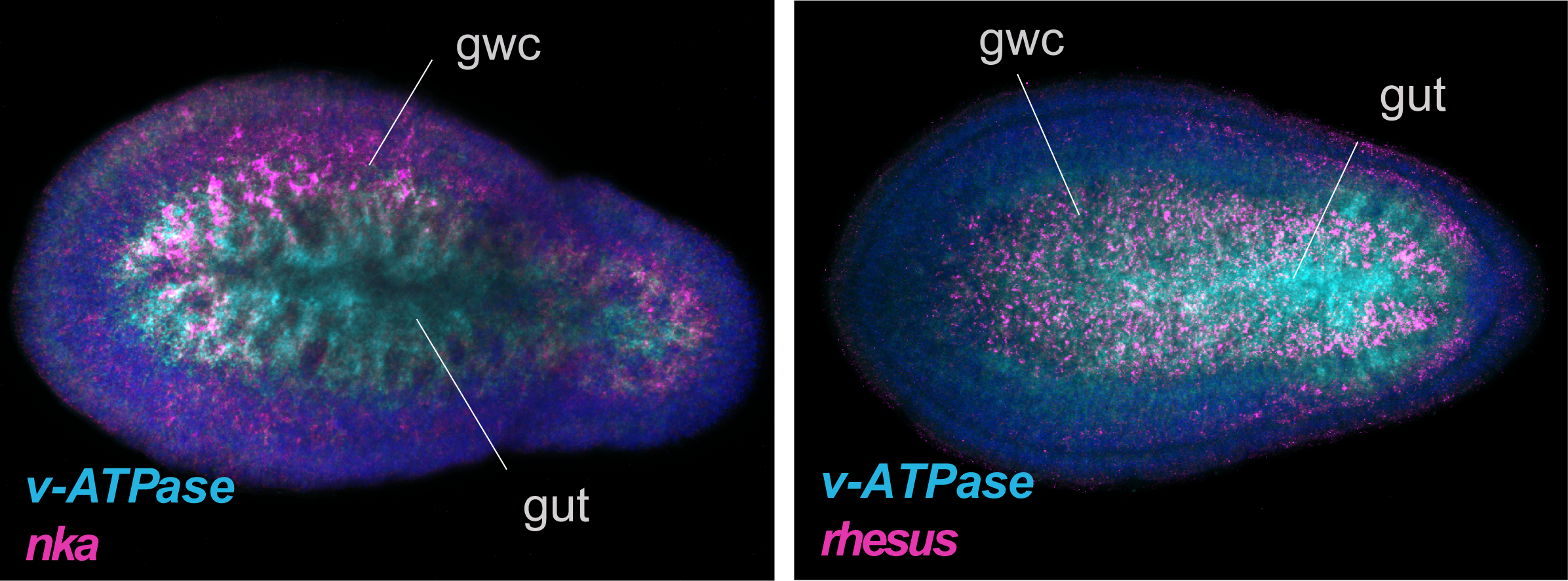

Supplement: S7 Fig — Every picture is a full projection of merged confocal stacks. Nuclei are stained blue with DAPI. Anterior is to the left. DAPI, 4',6-diamidino-2-phenylindole; dlr, distal lateral row; ds, digestive syncytium; gwc, gut-wrapping cell; nka, Na+/K+[NH4+] ATPase; v-ATPase, vacuolar H+-ATPase proton pump; WMISH, whole-mount in situ hybridization. (TIF) [file pbio.3000408.s007.tif]

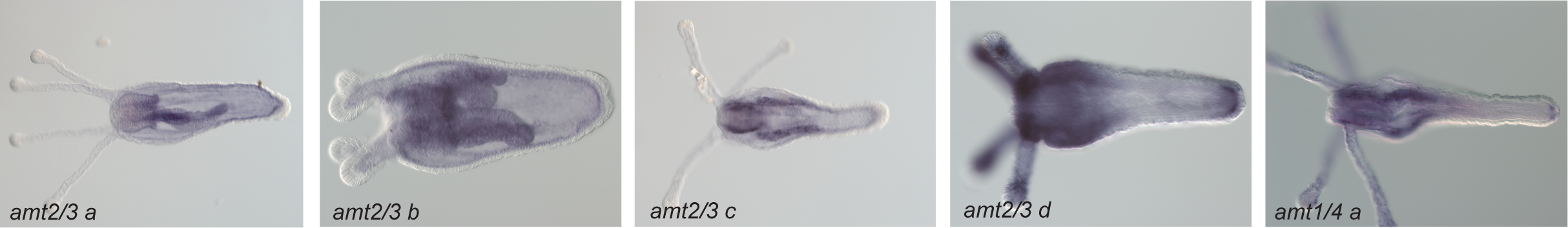

Supplement: S8 Fig — Gene expression of amt2/3a, amt2/3b, amt2/3c, amt2/3d, and amt1/4a in juvenile polyps. Anterior is to the left. amt, ammonia transporter; WMISH, whole-mount in situ hybridization. (TIF) [file pbio.3000408.s008.tif]

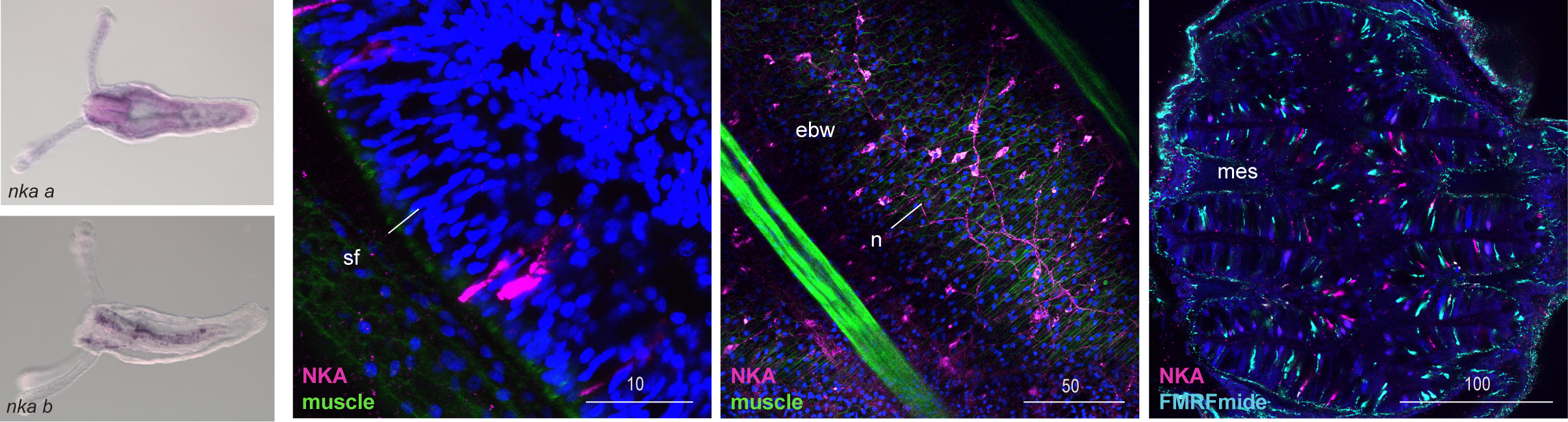

Supplement: S9 Fig — Gene expression of nka a and nka b in juvenile polyps. Anterior is to the left. Protein localization of NKA in N. vectensis juvenile polyps. The muscle filaments are labeled green with phalloidin, and the nervous system is stained cyan with tyrosinated tubulin. Every picture is a full projection of merged confocal stacks. Nuclei are stained blue with DAPI. The regions shown are indicated with dashed boxes in the illustrated animal. DAPI, 4',6-diamidino-2-phenylindole; ebw, endodermal body wall; mes, mesenteries; n, neuron; NKA, Na+/K+[NH4+] ATPase; sf, septal filament; WMISH, whole-mount in situ hybridization. (TIF) [file pbio.3000408.s009.tif]

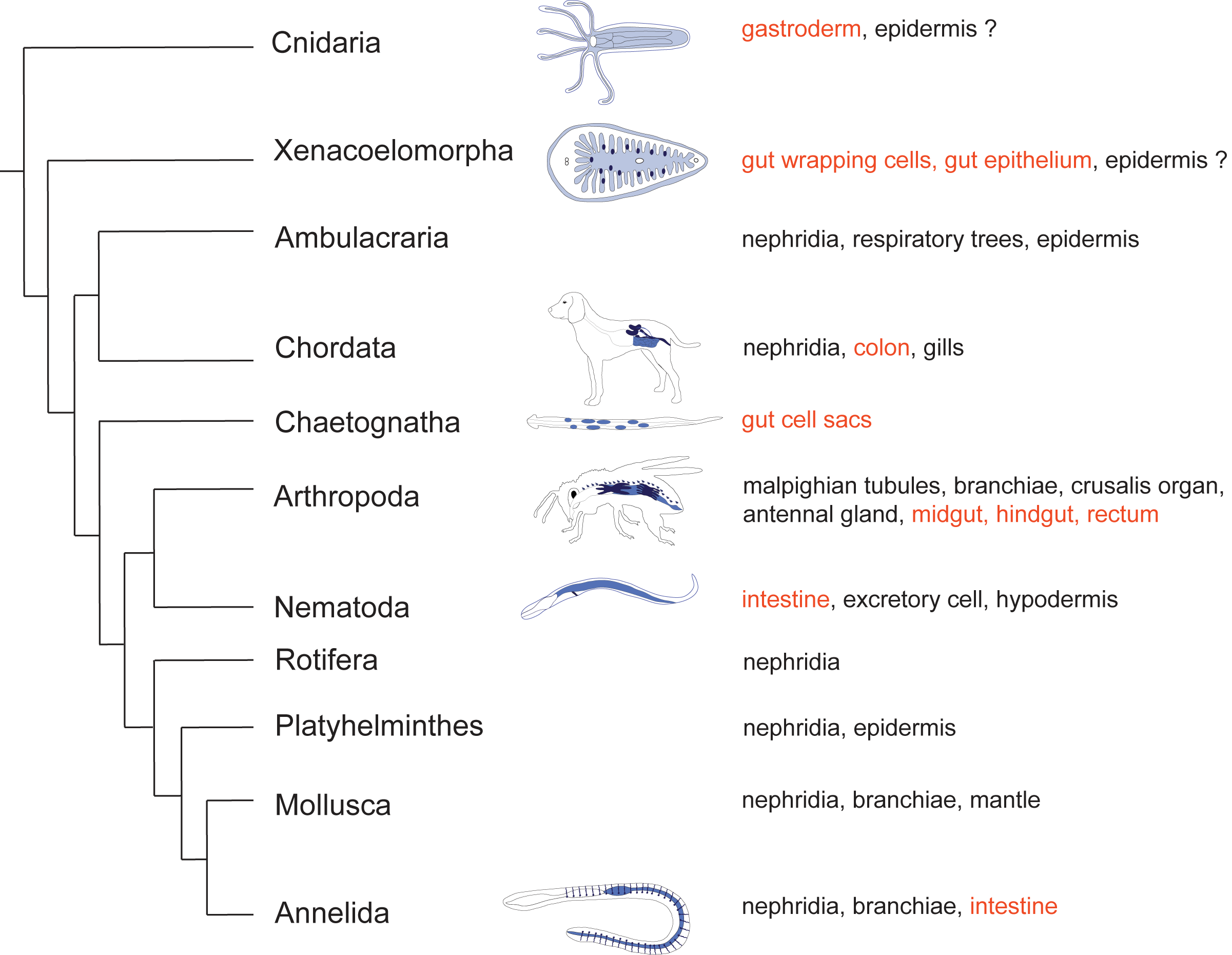

Supplement: S10 Fig — (TIF) [file pbio.3000408.s010.tif]
