# Supplementary material for: Active mode of excretion across digestive tissues predates the origin of excretory organs
Source: PLoS Biol. 2019 Jul 29;17(7):e3000408. doi: 10.1371/journal.pbio.3000408 (PMC6687202; doi:10.1371/journal.pbio.3000408)
Supplement: S1 Table — Data are based on this study unless stated otherwise. Question marks represent missing data. (PDF) [file pbio.3000408.s011.pdf]

	Nephrin/Kirre	***Cd2ap/Cin8***	***Zo1***	***Slipins***	***Aquaporins***
**Arthropoda**	nephrocytes [1], eye [2], muscle [5], CNS [8]	nephrocytes [1], eye [3], germline [6], epithelial sheet [9]	nephrocytes [1], eye [4], germline, adult wing [10], epithelia [11]	nephrocytes [1]	malpighian tubules, fat body, pharynx, epidermis, ovaries, gut (sum. in [7])
**Platyhelminthes**	terminal cells [12], stem cells [13]	?	?	?	ubiquitous, brain [14]
**Mollusca**	rhogocytes [15]	?	?	?	?
**Annelida**	?	?	?	?	?
**Nematoda**	neural axons, vulval muscle [16]	?	?	melanosensory neurons [17]	excretory cell, neurons, intestine, pharynx, muscle, hypodermis [18]
**Acoela**	neurons, brain, gonadal domain	neurons	brain, neurons, mouth	digestive syncytium, brain	brain, digestive syncytium, gut wrapping cells, parenchyme
**Nemertodermatida**	neurons	subepidermis, posterior lateral rows of cells anterior domain, mouth	gut epithelium, mouth	neurons, mouth, subepidermis	subepidermal cells, epidermis gut wrapping cells, brain
**Cnidaria**	absent	?	ectoderm [19]	?	?
**Vertebrata**	podocytes, muscles, CNS, testis, pancreatic cells, cardiovascular development, spleen, lymph node (sum. in [24])	podocytes [20], brain, heart, pancreas, salivary gland [23]	podocytes [21], eye [22], epithelial sheet, notochord, vascular development, neural tube [27]	podocytes, erythrocyte membranes, dorsal root ganglia, sensory neurons, skin (sum. in [25])	blood, kidney, brain, testis, oocyte oocyte eyes, pancreas, muscle, lung, digestive tract (sum. in [26]),
**Eubacteria/Archaea**	absent	absent	absent [28]	? [29]	osmoregulation [30]
**Filasterea/ Choanoflagellata**	absent	?	? [28]	? ([29], this study)	? [30]
**Holomycota**	absent	absent	absent [28]	? [29]	spores/ endoplasmic reticulum plasma membrane (sum. in [31])

	Rh/AMTs	*Na^+/K^+ ATPase*	*CA a (cytosol & membrane)*	*V-ATPase A/B*
**Arthropoda**	branchiae [32],<br>brain, salivary glands[34],<br>head, thorax [38],<br>fat body, head, midgut, hindgut, eye<br>malpighian tubules, ganglia, trachea,<br>[44, 45]	malpighian tubules, rectum,<br>midgut, hindgut, anal papillae<br>[35], Johnston's<br>organ of hearing [39],<br>Crusalis organs [41]<br>testis, eye, brain [43],<br>[34], branchiae [45, 48]	midgut and hindgut<br>(sum. in [33]),<br>malpighian tubules, branchiae<br>(sum. in [40]),<br>epidermis [42]	malpighian tubules, fat body, midgut,<br>hindgut, ganglia, trachea, antennal gland<br>[36, 37],<br>anal papilla [35], branchiae<br>(sum. in [40]),<br>Crusalis organs [41], neurons, [46, 47]<br>brain, testis, ovarioles, duct [49]
**Platyzoa**	epidermis [50],<br>duct of protonephridia [12]	epidermis [50],<br>protonephridia [51]	epidermis [50],<br>protonephridia [51]	epidermis [50]
**Mollusca**	epidermis [52],<br>branchiae [55]	foot muscle, hepatopancreas<br>[56], branchiae,<br>pancreatic appendages, nerves,<br>nephridia [58, 59]<br>mantle [57]	mantle [53],<br>branchiae [54]	branchiae [54],<br>mantle [57]
**Annelida**	epidermis [60],<br>branchiae [61]	branchiae [61],<br>epidermis (sum. in [62])	epidermal glands [53],<br>branchiae [61],<br>epidermis [60]	branchiae [61],<br>epidermis [60]
**Nematoda**	hypodermis	hypodermis [64]	intestine [64]	hypodermis, intestine, neurons [63]
**Acoela**	gut wrapping cells, neurons, brain,<br>parenchyme, mouth, epidermis	gut wrapping cells	mouth, parenchyme, neurons<br>brain, posterior cells, gut wrapping cells	digestive syncytium
**Nemertodermatida**	gut wrapping cells, brain,<br>epidermis	gut epithelium	gut wrapping cells, gut epithelium,<br>epidermis, neurons	gut epithelium, subepidermis, nerve cords
**Cnidaria**	tentacular ectodermal cells,<br>gastroderm, mesenteries, pharynx	pharyngeal endoderm, mesenteries,<br>neurons, septal filament, pharynx	tentacular ectoderm, mesenteries,<br>gastroderm (this study), oral and<br>aboral endoderm, calicoblastic cells,<br>ectoderm (sum. in [65])	gastroderm, mesenteries, pharynx,<br>septal filaments
**Vertebrata**	blood, esophagael epithelia, brain,<br>kidney duct, liver, gastrointestinal tract<br>testis (sum. in [70]),<br>gills [74, 75],<br>muscle [77],<br>skin [71]	kidney tubule and duct<br>[67, 68],<br>skin [71], intestine,<br>gut, eye, heart, testis, liver, brain<br>[78, 79],<br>gills [83, 84]	brain, bones, muscle, intestine,<br>kidney intercalated cells, tubule,<br>duct, eye, erythrocytes, liver,<br>urinary bladder, teeth, inner ear<br>(sum. in [80]), gills<br>[44, 74]	pancreas [66],<br>kidney tubule and duct [69],<br>eye [72, 73],<br>osteoclast [76], skin<br>[81], testis [82],<br>brain [85], gills<br>[44, 74]
**Eubacteria/Archaea**	NH_3 transport [86]	osmoregulation<br>[88, 89]	photosynthesis, CO_2 fixation<br>[90]	proton pump [87]
**Filasterea/<br>Choanoflagellata**	NH_3 transporter [91]	osmoregulation<br>[88, 89]	?	?
**Holomycota**	NH_3 transport<br>[93, 94]	osmoregulation [92]	CO_2 sensing system [95]	vacuole, Golgi/endosome [96]

	SLC1	***SLC4***	***SLC5***	***SLC8***
**Arthropoda**	eye, central nervous system [102], [98, 99] glia cells [105]	alimentary canal [97], malpighian tubules [103], heart [106], neurons [108]	malpighian tubules [100], glia cells [109] intestine [104], hepatopancreas [107], glia cells [109]	retina, brain [101]
**Platyzoa**	protonephridia [12]	protonephridia [12]	protonephridia [12]	protonephridia [12]
**Mollusca**	nervous system [111]	branchiae ([54], heart, neurons, optic lobe, testis [113]	hepatopancreas [110], gills, mantle edge [114]	muscle, neurons [112] [115]
**Annelida**	central nervous system, glia cells [116]	mesoderm, epidermis muscle, mesenchyme [117], neurons [118]	bacteriocytes [117]	?
**Nematoda**	muscle, pharynx, head neurons, excretory canal [120]	neurons, hypodermis, muscle [121]	neurons [119]	neurons, muscle, intestine [122]
**Acoela**	brain, gonadal domain	parenchyme, brain anterior tip	parenchyme, brain gonadal domain	anterior tip
**Nemertodermatida**	neurons	nerve cords, mouth, female gonads, subepidermis, gut wrapping cells	posterior lateral rows of cells, subepidermis	gut epithelium, mouth, posterior lateral rows of cells
**Cnidaria**	?	oral/aboral endoderm and ectoderm, calicoblastic ectoderm [123]	?	?
**Vertebrata**	kidney, intestine, brain (sum. in [124])	brain, kidney duct and tubule, ovary, testis, salivary gland, erythrocytes, heart, intestine, heart, pancreas, liver, smooth muscle, lung, retina, spleen (sum. in [126]), gill [128]	kidney, intestine, salivary glands, brain, retina, muscle, heart, uterus, testis, lung, placenta, liver (sum. in [127])	brain, heart, kidney, pancreas, muscle, liver (sum. in [125]
**Eubacteria/Archaea**	glytamate transport [130]	NO_2/ NO_3 exchange, Cl^- influx [131]	absent [129]	Na^+/ Ca^{2+} exchange [132]
**Filasterea/ Choanoflagellata**	absent ([129], this study)	?	absent ([129], this study)	([133], this study)
**Holomycota**	absent [129]	borate transport (Jennings et al 2007) [131]	absent [129]	absent [134] [135]

	SLC9	*SLC12*	*SLC13*	*SLC26*
**Arthropoda**	midgut [37], gill [136], malpighian tubules [141]	salivary gland, ventral nerve cord, gut, anal pad [137], malpighian tubules [142], glia cells [143]	fat body, oenocytes, midgut [138]	gut epithelial cells, malpighian tubules ([139]), auditory organs [140]
**Platyzoa**	protonephridia [12]	protonephridia [12]	protonephridia [12]	protonephridia [12]
**Mollusca**	mantle [57]	?	?	mantle [57]
**Annelida**	?	?	root epidermis [117]	?
**Nematoda**	hypodermis, intestine, excretory cell, neurons [146]	muscle, neurons, intestine, excretory cell [144] [147]	intestinal tract [145]	muscle, neurons, midgut, intestine, body wall, pharynx, excretory cell [121]
**Acoela**	mouth	parenchyme, brain	brain, parenchyme	no expression revealed
**Nemertodermatida**	nerve cords	female gonads, testis	neurons	female gonads, gut-wrapping cells
**Cnidaria**	?	?	?	oral tissues [123]
**Vertebrata**	gill [148], intestine stomach, endothelial cells, distal tubule of kidney, cardiac myocytes, gall bladder, epididymis, ovary, thymus, brain, pancreas, salivary gland, skin, sperm, osteoclasts, skeletal muscle (sum. in [152])	gill [148], intestine [84], ubiquitous, tubules of kidney, neurons, brain, bone (sum. in [151])	kidney, small intestine, liver, placenta, brain (sum. in [149])	hepatocytes, proximal tubule, intestine, testis, cardiac myocytes, brain, endothelial cells, hair cells, cochlear hair cells, pancreatic duct, thyrocytes (sum. in [150], gill [148], auditory organs [140]
**Eubacteria/Archaea**	ph regulation, metabolism, salt tolerance [153]	? [129] [154]	carbon dicarboxylate substrate transport [155]	HCO_3^-, C^4-dicarboxylic acid metabolism [156]
**Filasterea/ Choanoflagellata**	? ([129], this study))	? [129] [154]	absent ([129], this study)	? ([129], this study)
**Holomycota**	vacuole trafficking, regulation of cytosolic ph [153]	? [129]	? [157] [129]	? [131]

HCN

**Arthropoda**	gills [158], olfactory neurons [159], chemo-sensitivity [160]
**Platyzoa**	?
**Mollusca**	?
**Annelida**	?
**Nematoda**	hypodermis
**Acoela**	brain
**Nemertodermatida**	gut epithelium
**Cnidaria**	?
**Vertebrata**	kidney [161, 162], liver [163], ovary [164], pancreatic cells [165], heart [166], brain [167]
**Eubacteria/Archaea**	intracellular cAMP, pH sensor, osmoregulation [168, 169]
**Filasterea/ Choanoflagellata**	?
**Holomycota**	?

Supplementary references

1. Weavers H, Prieto-Sanchez S, Grawe F, Garcia-Lopez A, Artero R, Wilsch-Brauninger M, et al. The insect nephrocyte is a podocyte-like cell with a filtration slit diaphragm. Nature. 2009;457(7227):322-6.
2. Ramos RG, Igloi GL, Lichte B, Baumann U, Maier D, Schneider T, et al. The irregular chiasm C-roughest locus of *Drosophila*, which affects axonal projections and programmed cell death, encodes a novel immunoglobulin-like protein. Genes Dev. 1993;7(12B):2533-47.
3. Johnson RI, Seppa MJ, Cagan RL. The *Drosophila* CD2AP/CIN85 orthologue Cindr regulates junctions and cytoskeleton dynamics during tissue patterning. J Cell Biol. 2008;180(6):1191-204.
4. Seppa MJ, Johnson RI, Bao S, Cagan RL. Polychaetoid controls patterning by modulating adhesion in the *Drosophila* pupal retina. Dev Biol. 2008;318(1):1-16.
5. Strunkelnberg M, Bonengel B, Moda LM, Hertenstein A, de Couet HG, Ramos RG, et al. rst and its paralogue kirre act redundantly during embryonic muscle development in *Drosophila*. Development. 2001;128(21):4229-39.
6. Eikenes AH, Brech A, Stenmark H, Haglund K. Spatiotemporal control of Cindr at ring canals during incomplete cytokinesis in the *Drosophila* male germline. Dev Biol. 2013;377(1):9-20.
7. Campbell EM, Ball A, Hoppler S, Bowman AS. Invertebrate aquaporins: a review. Journal of comparative physiology B, Biochemical, systemic, and environmental physiology. 2008;178(8):935-55.
8. Dworak HA, Charles MA, Pellerano LB, Sink H. Characterization of *Drosophila hibris*, a gene related to human nephrin. Development. 2001;128(21):4265-76.
9. Yasin HW, van Rensburg SH, Feiler CE, Johnson RI. The adaptor protein Cindr regulates JNK activity to maintain epithelial sheet integrity. Dev Biol. 2016;410(2):135-49.
10. Djiane A, Shimizu H, Wilkin M, Mazleyrat S, Jennings MD, Avis J, et al. Su(dx) E3 ubiquitin ligase-dependent and -independent functions of polychaetoid, the *Drosophila* ZO-1 homologue. J Cell Biol. 2011;192(1):189-200.
11. Wei X, Ellis HM. Localization of the *Drosophila* MAGUK protein Polychaetoid is controlled by alternative splicing. Mech Dev. 2001;100(2):217-31.
12. Thi-Kim Vu H, Rink JC, McKinney SA, McClain M, Lakshmanaperumal N, Alexander R, et al. Stem cells and fluid flow drive cyst formation in an invertebrate excretory organ. eLife. 2015;4:e07405.
13. Nakamura T, Takagi S, Matsumoto M, Tashiro F, Sakai T, Ichimura K. Expression of Nephrin Homologue in the Freshwater Planarian, *Dugesia japonica*. Acta Histochem Cytochem. 2014;47(6):303-10.
14. Hwang B, An Y, Agata K, Umesono Y. Two distinct roles of the yorkie/yap gene during homeostasis in the planarian *Dugesia japonica*. Dev Growth Differ. 2015;57(3):209-17.
15. Kokkinopoulou M, Guler MA, Lieb B, Barbeck M, Ghanaati S, Markl J. 3D-ultrastructure, functions and stress responses of gastropod (*Biomphalaria glabrata*) rhogocytes. PloS one. 2014;9(6):e101078.
16. Wanner N, Noutsou F, Baumeister R, Walz G, Huber TB, Neumann-Haefelin E. Functional and spatial analysis of *C. elegans* SYG-1 and SYG-2, orthologs of the Neph/nephrin cell adhesion module directing selective synaptogenesis. PloS one. 2011;6(8):e23598.
17. Zhang S, Arnadottir J, Keller C, Caldwell GA, Yao CA, Chalfie M. MEC-2 is recruited to the putative mechanosensory complex in *C. elegans* touch receptor neurons through its stomatin-like domain. Curr Biol. 2004;14(21):1888-96.
18. Huang CG, Lamitina T, Agre P, Strange K. Functional analysis of the aquaporin gene family in *Caenorhabditis elegans*. Am J Physiol Cell Physiol. 2007;292(5):C1867-73.
19. Fei K, Yan L, Zhang J, Sarras MP, Jr. Molecular and biological characterization of a zonula occludens-1 homologue in *Hydra vulgaris*, named HZO-1. Dev Genes Evol. 2000;210(12):611-6.
20. Shih NY, Li J, Karpitskii V, Nguyen A, Dustin ML, Kanagawa O, et al. Congenital nephrotic syndrome in mice lacking CD2-associated protein. Science. 1999;286(5438):312-5.
21. Itoh M, Nakadate K, Horibata Y, Matsusaka T, Xu J, Hunziker W, et al. The structural and functional organization of the podocyte filtration slits is regulated by Tjp1/ZO-1. PloS one. 2014;9(9):e106621.
22. Sugrue SP, Zieske JD. ZO1 in corneal epithelium: association to the zonula occludens and adherens junctions. Exp Eye Res. 1997;64(1):11-20.
23. Li C, Ruotsalainen V, Tryggvason K, Shaw AS, Miner JH. CD2AP is expressed with nephrin in developing podocytes and is found widely in mature kidney and elsewhere. Am J Physiol Renal Physiol. 2000;279(4):F785-92.
24. Li X, He JC. An update: the role of Nephrin inside and outside the kidney. Sci China Life Sci. 2015;58(7):649-57.
25. Lapatsina L, Brand J, Poole K, Daumke O, Lewin GR. Stomatin-domain proteins. Euro J Cell Biol. 2012;91(4):240-5.

26. Ishibashi K, Kondo S, Hara S, Morishita Y. The evolutionary aspects of aquaporin family. Am J Physiol Regul Integr Comp Physiol. 2011;300(3):R566-76.
27. Bauer H, Zweimueller-Mayer J, Steinbacher P, Lametschwandtner A, Bauer HC. The dual role of zonula occludens (ZO) proteins. J Biomed Biotech. 2010;2010:402593.
28. de Mendoza A, Suga H, Ruiz-Trillo I. Evolution of the MAGUK protein gene family in premetazoan lineages. BMC Evol Biol. 2010;10:93.
29. Green JB, Young JP. Slipins: ancient origin, duplication and diversification of the stomatin protein family. BMC Evol Biol. 2008;8:44.
30. Abascal F, Irisarri I, Zardoya R. Diversity and evolution of membrane intrinsic proteins. Biochim Biophys Acta. 2014;1840(5):1468-81.
31. Gomes D, Agasse A, Thiébaud P, Delrot S, Geros H, Chaumont F. Aquaporins are multifunctional water and solute transporters highly divergent in living organisms. Biochim Biophys Acta. 2009;1788(6):1213-28.
32. Martin M, Fehsenfeld S, Sourial MM, Weihrauch D. Effects of high environmental ammonia on branchial ammonia excretion rates and tissue Rh-protein mRNA expression levels in seawater acclimated Dungeness crab *Metacarcinus magister*. Comp Biochem Physiol A. 2011;160(2):267-77.
33. Linser PJ, Smith KE, Seron TJ, Neira Oviedo M. Carbonic anhydrases and anion transport in mosquito midgut pH regulation. J Exp Biol. 2009;212(Pt 11):1662-71.
34. Chintapalli VR, Wang J, Dow JA. Using FlyAtlas to identify better *Drosophila melanogaster* models of human disease. Nat Genet. 2007;39(6):715-20.
35. Patrick ML, Aimanova K, Sanders HR, Gill SS. P-type Na+/K+-ATPase and V-type H+-ATPase expression patterns in the osmoregulatory organs of larval and adult mosquito *Aedes aegypti*. J Exp Biol. 2006;209(Pt 23):4638-51.
36. Tsai JR, Lin HC. Functional anatomy and ion regulatory mechanisms of the antennal gland in a semi-terrestrial crab, *Ocypode stimpsoni*. Biol Open. 2014;3(6):409-17.
37. Blaesse AK, Broehan G, Meyer H, Merzendorfer H, Weihrauch D. Ammonia uptake in *Manduca sexta* midgut is mediated by an amiloride sensitive cation/proton exchanger: Transport studies and mRNA expression analysis of NHE7, 9, NHE8, and V-ATPase (subunit D). Comp Biochem Phys A. 2010;157(4):364-76.
38. Wu Y, Zheng X, Zhang M, He A, Li Z, Zhan X. Cloning and functional expression of Rh50-like glycoprotein, a putative ammonia channel, in *Aedes albopictus* mosquitoes. J Insect Physiol. 2010;56(11):1599-610.
39. Roy M, Sivan-Loukianova E, Eberl DF. Cell-type-specific roles of Na+/K+ ATPase subunits in *Drosophila* auditory mechanosensation. Proc Natl Acad Sci U S A. 2013;110(1):181-6.
40. Weihrauch D, O'Donnell MJ. Links between Osmoregulation and Nitrogen-Excretion in Insects and Crustaceans. Integ Comp Biol. 2015;55(5):816-29.
41. Gerber L, Lee CE, Grousset E, Blondeau-Bidet E, Boucheker NB, Lorin-Nebel C, et al. The Legs Have It: In Situ Expression of Ion Transporters V-Type H(+)-ATPase and Na(+)/K(+)-ATPase in the Osmoregulatory Leg Organs of the Invading Copepod *Eurytemora affinis*. Physiol Biochem Zool. 2016;89(3):233-50.
42. Calhoun S, Zou E. Epidermal carbonic anhydrase activity and exoskeletal metal content during the molting cycle of the blue crab, *Callinectes sapidus*. J Exp Zool A Ecol Genet Physiol. 2016;325(3):200-8.
43. Yasuhara JC, Baumann O, Takeyasu K. Localization of Na/K-ATPase in developing and adult *Drosophila melanogaster* photoreceptors. Cell Tissue Res. 2000;300(2):239-49.
44. Weihrauch D, Wilkie MP, Walsh PJ. Ammonia and urea transporters in gills of fish and aquatic crustaceans. J Exp Biol. 2009;212(Pt 11):1716-30.
45. Weihrauch D. Active ammonia absorption in the midgut of the tobacco hornworm *Manduca sexta* L.: Transport studies and mRNA expression analysis of a Rhesus-like ammonia transporter. Insect Biochem Mol Biol. 2006;36(10):808-21.
46. Williamson WR, Yang T, Terman JR, Hiesinger PR. Guidance receptor degradation is required for neuronal connectivity in the Drosophila nervous system. PLoS Biol. 2010;8(12):e1000553.
47. Williamson WR, Wang D, Haberman AS, Hiesinger PR. A dual function of V0-ATPase a1 provides an endolysosomal degradation mechanism in *Drosophila melanogaster* photoreceptors. J Cell Biol. 2010;189(5):885-99.
48. Weihrauch D, Becker W, Postel U, Luck-Kopp S, Siebers D. Potential of active excretion of ammonia in three different haline species of crabs. J Comp Physiol B. 1999;169(1):25-37.
49. Allan AK, Du J, Davies SA, Dow JA. Genome-wide survey of V-ATPase genes in *Drosophila* reveals a conserved renal phenotype for lethal alleles. Physiol Genomics. 2005;22(2):128-38.

50. Weihrauch D, Chan AC, Meyer H, Doring C, Sourial M, O'Donnell MJ. Ammonia excretion in the freshwater planarian *Schmidtea mediterranea*. J Exp Biol. 2012;215(Pt 18):3242-53.
51. Scimone ML, Srivastava M, Bell GW, Reddien PW. A regulatory program for excretory system regeneration in planarians. Development. 2011;138(20):4387-98.
52. Hu MY, Lee JR, Lin LY, Shih TH, Stumpp M, Lee MF, et al. Development in a naturally acidified environment: Na+/H+-exchanger 3-based proton secretion leads to CO2 tolerance in cephalopod embryos. Front Zool. 2013;10(1):51.
53. Le Roy N, Jackson DJ, Marie B, Ramos-Silva P, Marin F. The evolution of metazoan alpha-carbonic anhydrases and their roles in calcium carbonate biomineralization. Front Zool. 2014;11.
54. Hu MY, Tseng YC, Stumpp M, Gutowska MA, Kiko R, Lucassen M, et al. Elevated seawater PCO(2) differentially affects branchial acid-base transporters over the course of development in the cephalopod *Sepia officinalis*. Am J Physiol Regul Integr Comp Physiol. 2011;300(5):R1100-14.
55. Hu MY, Casties I, Stumpp M, Ortega-Martinez O, Dupont S. Energy metabolism and regeneration are impaired by seawater acidification in the infaunal brittlestar *Amphiura filiformis*. J Exp Biol. 2014;217(Pt 13):2411-21.
56. Ramnanan CJ, Storey KB. Suppression of Na+/K+-ATPase activity during estivation in the land snail *Otala lactea*. J Exp Biol. 2006;209(4):677-88.
57. Li S, Liu C, Huang J, Liu Y, Zhang S, Zheng G, et al. Transcriptome and biomineralization responses of the pearl oyster *Pinctada fucata* to elevated CO2 and temperature. Sci Rep. 2016;6:18943.
58. Hu MY, Sung PH, Guh YJ, Lee JR, Hwang PP, Weihrauch D, et al. Perfused Gills Reveal Fundamental Principles of pH Regulation and Ammonia Homeostasis in the Cephalopod *Octopus vulgaris*. Front Physiol. 2017;8:162.
59. Hu MY, Sucre E, Charmantier-Daures M, Charmantier G, Lucassen M, Himmerkus N, et al. Localization of ion-regulatory epithelia in embryos and hatchlings of two cephalopods. Cell Tissue Res. 2010;339(3):571-83.
60. Quijada-Rodriguez AR, Treberg JR, Weihrauch D. Mechanism of ammonia excretion in the freshwater leech *Nephelopsis obscura*: characterization of a primitive Rh protein and effects of high environmental ammonia. Am J Physiol Regul Integr Comp Physiol. 2015;309(6):R692-705.
61. Thiel D, Hugenschutt M, Meyer H, Paululat A, Quijada-Rodriguez AR, Purschke G, et al. Ammonia excretion in the marine polychaete *Eurythoe complanata* (Annelida). J Exp Biol. 2016;220(Pt 3):425-36.
62. Schnizler M, Krumm S, Clauss W. Annelid epithelia as models for electrogenic Na+ transport. Biochim Biophys Acta. 2002;1566(1-2):84-91.
63. Oka T, Toyomura T, Honjo K, Wada Y, Futai M. Four subunit a isoforms of *Caenorhabditis elegans* vacuolar H+-ATPase. Cell-specific expression during development. J Biol Chem. 2001;276(35):33079-85.
64. Adlimoghaddam A, Boeckstaens M, Marini AM, Treberg JR, Brassinga AKC, Weihrauch D. Ammonia excretion in *Caenorhabditis elegans*: mechanism and evidence of ammonia transport of the Rhesus protein CeRhr-1. J Exp Biol. 2015;218(5):675-83.
65. Bertucci A, Moya A, Tambutte S, Allemand D, Supuran CT, Zoccola D. Carbonic anhydrases in anthozoan corals-A review. Bioorg Medic Chem. 2013;21(6):1437-50.
66. Sun-Wada GH, Toyomura T, Murata Y, Yamamoto A, Futai M, Wada Y. The a3 isoform of V-ATPase regulates insulin secretion from pancreatic beta-cells. J Cell Sci. 2006;119(Pt 21):4531-40.
67. Wall SM, Koger LM. NH+4 transport mediated by Na(+)-K(+)-ATPase in rat inner medullary collecting duct. Am J Physiol. 1994;267(4 Pt 2):F660-70.
68. Garvin JL, Burg MB, Knepper MA. Ammonium replaces potassium in supporting sodium transport by the Na-K-ATPase of renal proximal straight tubules. Am J Physiol. 1985;249(5 Pt 2):F785-8.
69. Wagner CA, Finberg KE, Breton S, Marshansky V, Brown D, Geibel JP. Renal vacuolar H+-ATPase. Physiol Rev. 2004;84(4):1263-314.
70. Huang CH, Ye M. The Rh protein family: gene evolution, membrane biology, and disease association. Cell Mol Life Sci. 2010;67(8):1203-18.
71. Cruz MJ, Sourial MM, Treberg JR, Fehsenfeld S, Adlimoghaddam A, Weihrauch D. Cutaneous nitrogen excretion in the African clawed frog *Xenopus laevis*: effects of high environmental ammonia (HEA). Aquatic toxicology. 2013;136-137:1-12.
72. Kawamura N, Tabata H, Sun-Wada GH, Wada Y. Optic nerve compression and retinal degeneration in Tcirg1 mutant mice lacking the vacuolar-type H-ATPase a3 subunit. PloS one. 2010;5(8):e12086.
73. Jouhou H, Yamamoto K, Homma A, Hara M, Kaneko A, Yamada M. Depolarization of isolated horizontal cells of fish acidifies their immediate surrounding by activating V-ATPase. J Physiol. 2007;585(Pt 2):401-12.

74. Nawata CM, Hung CC, Tsui TK, Wilson JM, Wright PA, Wood CM. Ammonia excretion in rainbow trout (*Oncorhynchus mykiss*): evidence for Rh glycoprotein and H+-ATPase involvement. Physiol Genomics. 2007;31(3):463-74.
75. Nakada T, Westhoff CM, Kato A, Hirose S. Ammonia secretion from fish gill depends on a set of Rh glycoproteins. FASEB J. 2007;21(4):1067-74.
76. Toyomura T, Murata Y, Yamamoto A, Oka T, Sun-Wada GH, Wada Y, et al. From lysosomes to the plasma membrane: localization of vacuolar-type H+ -ATPase with the a3 isoform during osteoclast differentiation. J Biol Chem. 2003;278(24):22023-30.
77. Takeda K, Takemasa T. Expression of ammonia transporters Rhbg and Rhcg in mouse skeletal muscle and the effect of 6-week training on these proteins. Physiol Rep. 2015;3(10).
78. Worrell RT, Merk L, Matthews JB. Ammonium transport in the colonic crypt cell line, T84: role for Rhesus glycoproteins and NKCC1. Am J Physiol Gastrointest Liver Physiol. 2008;294(2):G429-40.
79. Rahman MM, Tae HJ, Cho HS, Shin GW, Park BY. Developmental expression analysis of Na, K-ATPase alpha subunits in *Xenopus*. Dev Genes Evol. 2015;225(2):105-11.
80. McKenna R, Frost SC. Overview of the carbonic anhydrase family. Sub-cellular biochemistry. 2014;75:3-5.
81. Shih TH, Horng JL, Hwang PP, Lin LY. Ammonia excretion by the skin of zebrafish (*Danio rerio*) larvae. Am J Physiol Cell Physiol. 2008;295(6):C1625-32.
82. Pietrement C, Sun-Wada GH, Silva ND, McKee M, Marshansky V, Brown D, et al. Distinct expression patterns of different subunit isoforms of the V-ATPase in the rat epididymis. Biol Reprod. 2006;74(1):185-94.
83. Mallery CH. A carrier enzyme basis for ammonium excretion in teleost gill. NH+4-stimulated Na-dependent ATPase activity in *Opsanus beta*. Comp Biochem Physiol A Comp Physiol. 1983;74(4):889-97.
84. Li Z, Lui EY, Wilson JM, Ip YK, Lin Q, Lam TJ, et al. Expression of key ion transporters in the gill and esophageal-gastrointestinal tract of euryhaline Mozambique tilapia *Oreochromis mossambicus* acclimated to fresh water, seawater and hypersaline water. PloS one. 2014;9(1):e87591.
85. Moriyama Y, Futai M. H(+)-ATPase, a primary pump for accumulation of neurotransmitters, is a major constituent of brain synaptic vesicles. Biochem Biophys Res Commun. 1990;173(1):443-8.
86. Huang CH, Peng J. Evolutionary conservation and diversification of Rh family genes and proteins. Proc Natl Acad Sci U S A. 2005;102(43):15512-7.
87. Gogarten JP, Starke T, Kibak H, Fishman J, Taiz L. Evolution and isoforms of V-ATPase subunits. J Exp Biol. 1992;172:137-47.
88. Saez AG, Lozano E, Zaldivar-Riveron A. Evolutionary history of Na,K-ATPases and their osmoregulatory role. Genetica. 2009;136(3):479-90.
89. Chan H, Babayan V, Blyumin E, Gandhi C, Hak K, Harake D, et al. The p-type ATPase superfamily. J Mol Microbiol Biotech. 2010;19(1-2):5-104.
90. Smith KS, Ferry JG. Prokaryotic carbonic anhydrases. FEMS Microbiol Rev. 2000;24(4):335-66.
91. King N, Westbrook MJ, Young SL, Kuo A, Abedin M, Chapman J, et al. The genome of the choanoflagellate *Monosiga brevicollis* and the origin of metazoans. Nature. 2008;451(7180):783-8.
92. Benito B, Garciadeblas B, Rodriguez-Navarro A. Potassium- or sodium-efflux ATPase, a key enzyme in the evolution of fungi. Microbiology. 2002;148(Pt 4):933-41.
93. Marini AM, Soussi-Boudekou S, Vissers S, Andre B. A family of ammonium transporters in *Saccharomyces cerevisiae*. Mol Cell Biol. 1997;17(8):4282-93.
94. Marini AM, Matassi G, Raynal V, Andre B, Cartron JP, Cherif-Zahar B. The human Rhesus-associated RhAG protein and a kidney homologue promote ammonium transport in yeast. Nat, Genetics. 2000;26(3):341-4.
95. Elleuche S, Poggeler S. Carbonic anhydrases in fungi. Microbiology. 2010;156(Pt 1):23-9.
96. Kawasaki-Nishi S, Nishi T, Forgac M. Yeast V-ATPase complexes containing different isoforms of the 100-kDa a-subunit differ in coupling efficiency and in vivo dissociation. J Biol Chem. 2001;276(21):17941-8.
97. Linser PJ, Neira Oviedo M, Hirata T, Seron TJ, Smith KE, Piermarini PM, et al. Slc4-like anion transporters of the larval mosquito alimentary canal. J Insect Physiol. 2012;58(4):551-62.
98. Umesh A, Cohen BN, Ross LS, Gill SS. Functional characterization of a glutamate/aspartate transporter from the mosquito *Aedes aegypti*. J Exp Biol. 2003;206(Pt 13):2241-55.
99. Besson MT, Soustelle L, Birman S. Identification and structural characterization of two genes encoding glutamate transporter homologues differently expressed in the nervous system of *Drosophila melanogaster*. FEBS Lett. 1999;443(2):97-104.
100. Stergiopoulos K, Cabrero P, Davies SA, Dow JA. Salty dog, an SLC5 symporter, modulates *Drosophila* response to salt stress. Physiol Genomics. 2009;37(1):1-11.

101. Schwarz EM, Benzer S. Calx, a Na-Ca exchanger gene of *Drosophila melanogaster*. P Natl Acad Sci USA. 1997;94(19):10249-54.
102. Kucharski R, Ball EE, Hayward DC, Maleszka R. Molecular cloning and expression analysis of a cDNA encoding a glutamate transporter in the honeybee brain. Gene. 2000;242(1-2):399-405.
103. Yamahiro A, Piermarini PM, Beyenbach KW. Identification of Na-driven anion exchanger (NDAE) splice variants from Malpighian (renal) tubules of the adult yellow-fever mosquito. FASEB Journal. 2008;22.
104. Obi IE, Sterling KM, Ahearn GA. Transepithelial D-glucose and D-fructose transport across the American lobster, *Homarus americanus*, intestine. J Exp Biol. 2011;214(Pt 14):2337-44.
105. Soustelle L, Besson MT, Rival T, Birman S. Terminal glial differentiation involves regulated expression of the excitatory amino acid transporters in the *Drosophila* embryonic CNS. Dev Biol. 2002;248(2):294-306.
106. Perrin L, Monier B, Ponzielli R, Astier M, Semeriva M. *Drosophila* cardiac tube organogenesis requires multiple phases of Hox activity. Dev Biol. 2004;272(2):419-31.
107. Ahearn GA, Grover ML, Dunn RE. Glucose transport by lobster hepatopancreatic brush-border membrane vesicles. Am J Physiol. 1985;248(2 Pt 2):R133-41.
108. Romero MF, Henry D, Nelson S, Harte PJ, Dillon AK, Sciortino CM. Cloning and characterization of a Na+-driven anion exchanger (NDAE1). A new bicarbonate transporter. J Biol Chem. 2000;275(32):24552-9.
109. Freeman MR, Delrow J, Kim J, Johnson E, Doe CQ. Unwrapping glial biology: Gcm target genes regulating glial development, diversification, and function. Neuron. 2003;38(4):567-80.
110. Blaya JA, Muriana FJ, Ruiz-Gutierrez V, Vazquez CM, Bolufer J. Folate transport by prawn hepatopancreas brush-border membrane vesicles. Biosci Rep. 1998;18(1):9-17.
111. Hatakeyama D, Mita K, Kobayashi S, Sadamoto H, Fujito Y, Hiripi L, et al. Glutamate Transporters in the Central Nervous System of a Pond Snail. J Neurosci Res. 2010;88(6):1374-86.
112. Rasgado-Flores H, Espinosa-Tanguma R, Tie J, DeSantiago J. Voltage dependence of Na-Ca exchange in barnacle muscle cells. I. Na-Na exchange activated by alpha-chymotrypsin. Ann N Y Acad Sci. 1996;779:236-48.
113. Piermarini PM, Choi I, Boron WF. Cloning and characterization of an electrogenic Na/HCO3-cotransporter from the squid giant fiber lobe. Am J Physiol Cell Physiol. 2007;292(6):C2032-45.
114. Hanquet AC, Jouaux A, Heude C, Mathieu M, Kellner K. A sodium glucose co-transporter (SGLT) for glucose transport into *Crassostrea gigas* vesicular cells: Impact of alimentation on its expression. Aquaculture. 2011;313(1-4):123-8.
115. Rosenthal JJ, Gilly WF. Amino acid sequence of a putative sodium channel expressed in the giant axon of the squid Loligo opalescens. Proc Natl Acad Sci U S A. 1993;90(21):10026-30.
116. Hirth IC, Deitmer JW. 5-Hydroxytryptamine-mediated increase in glutamate uptake by the leech giant glial cell. Glia. 2006;54(8):786-94.
117. Miyamoto N, Yoshida MA, Koga H, Fujiwara Y. Genetic mechanisms of bone digestion and nutrient absorption in the bone-eating worm *Osedax japonicus* inferred from transcriptome and gene expression analyses. BMC Evol Biol. 2017;17(1):17.
118. Munsch T, Deitmer JW. Sodium-bicarbonate cotransport current in identified leech glial cells. J Physiol. 1994;474(1):43-53.
119. Okuda T, Haga T, Kanai Y, Endou H, Ishihara T, Katsura I. Identification and characterization of the high-affinity choline transporter. Nat Neurosci. 2000;3(2):120-5.
120. Mano I, Straud S, Driscoll M. *Caenorhabditis elegans* glutamate transporters influence synaptic function and behavior at sites distant from the synapse. J Biol Chem. 2007;282(47):34412-9.
121. Sherman T, Chernova MN, Clark JS, Jiang L, Alper SL, Nehrke K. The abts and sulp families of anion transporters from *Caenorhabditis elegans*. Am J Physiol Cell Physiol. 2005;289(2):C341-51.
122. Sharma V, He C, Sacca-Schaeffer J, Brzozowski E, Martin-Herranz DE, Mendelowitz Z, et al. Insight into the Family of Na+/Ca2+ Exchangers of *Caenorhabditis elegans*. Genetics. 2013;195(2):611-+.
123. Zoccola D, Ganot P, Bertucci A, Caminiti-Segonds N, Techer N, Voolstra CR, et al. Bicarbonate transporters in corals point towards a key step in the evolution of cnidarian calcification. Sci Rep. 2015;5:9983.
124. Grewer C, Gameiro A, Rauen T. SLC1 glutamate transporters. Pflügers Arch. 2014;466(1):3-24.
125. Khananshvili D. The SLC8 gene family of sodium-calcium exchangers (NCX) - structure, function, and regulation in health and disease. Mol Aspects Med. 2013;34(2-3):220-35.
126. Romero MF, Chen AP, Parker MD, Boron WF. The SLC4 family of bicarbonate (HCO(3)(-)) transporters. Mol Aspects Med. 2013;34(2-3):159-82.
127. Wright EM. Glucose transport families SLC5 and SLC50. Mol Aspects Med. 2013;34(2-3):183-96.

128. Lee YC, Yan JJ, Cruz SA, Horng JL, Hwang PP. Anion exchanger 1b, but not sodium-bicarbonate cotransporter 1b, plays a role in transport functions of zebrafish H+-ATPase-rich cells. Am J Physiol Cell Physiol. 2011;300(2):C295-307.
129. Höglund PJ, Nordstrom KJ, Schioth HB, Fredriksson R. The solute carrier families have a remarkably long evolutionary history with the majority of the human families present before divergence of bilaterian species. Mol Biol Evol. 2011;28(4):1531-41.
130. Yernool D, Boudker O, Jin Y, Gouaux E. Structure of a glutamate transporter homologue from *Pyrococcus horikoshii*. Nature. 2004;431(7010):811-8.
131. Parker MD, Boron WF. The divergence, actions, roles, and relatives of sodium-coupled bicarbonate transporters. Physiol Rev. 2013;93(2):803-959.
132. Liao J, Li H, Zeng W, Sauer DB, Belmares R, Jiang Y. Structural insight into the ion-exchange mechanism of the sodium/calcium exchanger. Science. 2012;335(6069):686-90.
133. Cai X, Clapham DE. Ancestral Ca2+ signaling machinery in early animal and fungal evolution. Mol Biol Evol. 2012;29(1):91-100.
134. Cai X, Lytton J. The cation/Ca(2+) exchanger superfamily: phylogenetic analysis and structural implications. Mol Biol Evol. 2004;21(9):1692-703.
135. Pittman JK, Hirschi KD. Phylogenetic analysis and protein structure modelling identifies distinct Ca(2+)/Cation antiporters and conservation of gene family structure within *Arabidopsis* and rice species. Rice. 2016;9(1):3.
136. Weihrauch D, Becker W, Postel U, Riestenpatt S, Siebers D. Active excretion of ammonia across the gills of the shore crab *Carcinus maenas* and its relation to osmoregulatory ion uptake. Journal of comparative physiology B, Biochemical, systemic, and environmental physiology. 1998;168(5):364-76.
137. Sun Q, Tian E, Turner RJ, Ten Hagen KG. Developmental and functional studies of the SLC12 gene family members from *Drosophila melanogaster*. Am J Physiol Cell Physiol. 2010;298(1):C26-37.
138. Inoue K, Fei YJ, Huang W, Zhuang L, Chen Z, Ganapathy V. Functional identity of *Drosophila melanogaster* Indy as a cation-independent, electroneutral transporter for tricarboxylic acid-cycle intermediates. Biochem J. 2002;367(Pt 2):313-9.
139. Hirata T, Czapar A, Brin L, Haritonova A, Bondeson DP, Linser P, et al. Ion and solute transport by Prestin in Drosophila and Anopheles. J Insect Physiol. 2012;58(4):563-9.
140. Weber T, Gopfert MC, Winter H, Zimmermann U, Kohler H, Meier A, et al. Expression of prestin-homologous solute carrier (SLC26) in auditory organs of nonmammalian vertebrates and insects. Proc Natl Acad Sci U S A. 2003;100(13):7690-5.
141. Kang'ethe W, Aimanova KG, Pullikuth AK, Gill SS. NHE8 mediates amiloride-sensitive Na(+)/H(+) exchange across mosquito Malpighian tubules and catalyzes Na(+) and K(+) transport in reconstituted proteoliposomes. Am J Physiol-Renal. 2007;292(5):F1501-F12.
142. Piermarini PM, Hine RM, Schepel M, Miyauchi J, Beyenbach KW. Role of an apical K,Cl cotransporter in urine formation by renal tubules of the yellow fever mosquito (*Aedes aegypti*). Am J Physiol Regul Integr Comp Physiol. 2011;301(5):R1318-37.
143. Leiserson WM, Forbush B, Keshishian H. *Drosophila* glia use a conserved cotransporter mechanism to regulate extracellular volume. Glia. 2011;59(2):320-32.
144. Tanis JE, Bellemer A, Moresco JJ, Forbush B, Koelle MR. The Potassium Chloride Cotransporter KCC-2 Coordinates Development of Inhibitory Neurotransmission and Synapse Structure in *Caenorhabditis elegans*. J Neurosci. 2009;29(32):9943-54.
145. Fei YJ, Inoue K, Ganapathy V. Structural and functional characteristics of two sodium-coupled dicarboxylate transporters (ceNaDC1 and ceNaDC2) from *Caenorhabditis elegans* and their relevance to life span. J Biol Chem. 2003;278(8):6136-44.
146. Nehrke K, Melvin JE. The NHX family of Na+-H+ exchangers in *Caenorhabditis elegans*. J Biol Chem. 2002;277(32):29036-44.
147. Spencer WC, Zeller G, Watson JD, Henz SR, Watkins KL, McWhirter RD, et al. A spatial and temporal map of *C. elegans* gene expression. Genome Res. 2011;21(2):325-41.
148. Hwang PP. Ion uptake and acid secretion in zebrafish (*Danio rerio*). J Exp Biol. 2009;212(Pt 11):1745-52.
149. Bergeron MJ, Clemencon B, Hediger MA, Markovich D. SLC13 family of Na(+)-coupled di- and tri-carboxylate/sulfate transporters. Mol Aspects Med. 2013;34(2-3):299-312.
150. Alper SL, Sharma AK. The SLC26 gene family of anion transporters and channels. Mol Aspects Med. 2013;34(2-3):494-515.
151. Arroyo JP, Kahle KT, Gamba G. The SLC12 family of electroneutral cation-coupled chloride cotransporters. Mol Aspects Med. 2013;34(2-3):288-98.

152. Donowitz M, Ming Tse C, Fuster D. SLC9/NHE gene family, a plasma membrane and organellar family of Na(+)/H(+) exchangers. Mol Aspects Med. 2013;34(2-3):236-51.
153. Brett CL, Donowitz M, Rao R. Evolutionary origins of eukaryotic sodium/proton exchangers. Am J Physiol Cell Physiol. 2005;288(2):C223-39.
154. Gagnon KB, Delpire E. Physiology of SLC12 transporters: lessons from inherited human genetic mutations and genetically engineered mouse knockouts. Am J Physiol-Cell Ph. 2013;304(8):C693-C714.
155. Hall JA, Pajor AM. Functional characterization of a Na(+)-coupled dicarboxylate carrier protein from *Staphylococcus aureus*. J Bacteriol. 2005;187(15):5189-94.
156. Compton EL, Page K, Findlay HE, Haertlein M, Moulin M, Zachariae U, et al. Conserved structure and domain organization among bacterial Slc26 transporters. Biochem J. 2014;463(2):297-307.
157. Saier MH, Jr., Eng BH, Fard S, Garg J, Haggerty DA, Hutchinson WJ, et al. Phylogenetic characterization of novel transport protein families revealed by genome analyses. Biochim Biophys Acta. 1999;1422(1):1-56.
158. Fehsenfeld S, Weihrauch D. The role of an ancestral hyperpolarization-activated cyclic nucleotide-gated K+ channel in branchial acid-base regulation in the green crab, *Carcinus maenas*. J Exp Biol. 2016;219(Pt 6):887-96.
159. Gisselmann G, Marx T, Bobkov Y, Wetzel CH, Neuhaus EM, Ache BW, et al. Molecular and functional characterization of an I(h)-channel from lobster olfactory receptor neurons. Eur J Neurosci. 2005;21(6):1635-47.
160. Chen Z, Wang Z. Functional study of hyperpolarization activated channel (Ih) in *Drosophila* behavior. Sci China Life Sci. 2012;55(1):2-7.
161. Carrisoza-Gaytan R, Rangel C, Salvador C, Saldana-Meyer R, Escalona C, Satlin LM, et al. The hyperpolarization-activated cyclic nucleotide-gated HCN2 channel transports ammonium in the distal nephron. Kidney Int. 2011;80(8):832-40.
162. Bolivar JJ, Tapia D, Arenas G, Castanon-Arreola M, Torres H, Galarraga E. A hyperpolarization-activated, cyclic nucleotide-gated, (Ih-like) cationic current and HCN gene expression in renal inner medullary collecting duct cells. Am J Physiol Cell Physiol. 2008;294(4):C893-906.
163. Santoro B, Tibbs GR. The HCN gene family: molecular basis of the hyperpolarization-activated pacemaker channels. Ann N Y Acad Sci. 1999;868:741-64.
164. Yeh J, Kim BS, Gaines L, Peresie J, Page C, Arroyo A. The expression of hyperpolarization activated cyclic nucleotide gated (HCN) channels in the rat ovary are dependent on the type of cell and the reproductive age of the animal: a laboratory investigation. Reprod Biol Endocrinol. 2008;6:35.
165. El-Kholy W, MacDonald PE, Fox JM, Bhattacharjee A, Xue T, Gao X, et al. Hyperpolarization-activated cyclic nucleotide-gated channels in pancreatic beta-cells. Mol Endocrinol. 2007;21(3):753-64.
166. DiFrancesco D. Pacemaker mechanisms in cardiac tissue. Annu Rev Physiol. 1993;55:455-72.
167. Luthi A, McCormick DA. H-current: properties of a neuronal and network pacemaker. Neuron. 1998;21(1):9-12.
168. Nimigean CM, Shane T, Miller C. A cyclic nucleotide modulated prokaryotic K+ channel. J Gen Physiol. 2004;124(3):203-10.
169. Kuo MM, Saimi Y, Kung C, Choe S. Patch clamp and phenotypic analyses of a prokaryotic cyclic nucleotide-gated K+ channel using *Escherichia coli* as a host. J Biol Chem. 2007;282(33):24294-301.
