# Supplementary material for: Active mode of excretion across digestive tissues predates the origin of excretory organs
Source: PLoS Biol. 2019 Jul 29;17(7):e3000408. doi: 10.1371/journal.pbio.3000408 (PMC6687202; doi:10.1371/journal.pbio.3000408)
Supplement: S2 Table — (PDF) [file pbio.3000408.s012.pdf]

pH meassurements

concentration [µmol/l]	Ipul	Nvec
0	8.14	8.53
50	8.14	8.53
100	8.14	8.52
200	8.14	8.50
500	8.12	8.46
1000	8.10	8.36

Nvec Excretion meassurements

sample	conc1 [µmol/l]	conc2 [µmol/l]
**pH = 8.53**	41,39786873	41,39786873
**pH = 8.36**	41,39786873	39,30019181
**pH = 8.53**	43,60751072	43,60751072
**pH = 8.36**	39,30019181	39,30019181
**pH = 8.53**	30,30247522	28,76701446
**pH = 8.36**	30,30247522	28,76701446

meassured: 2 hour incubation of 7 adult Nematostella in 8 ml medium

concentration: µmol NH4 / 2 ml*2h
