# Supplementary material for: Active mode of excretion across digestive tissues predates the origin of excretory organs
Source: PLoS Biol. 2019 Jul 29;17(7):e3000408. doi: 10.1371/journal.pbio.3000408 (PMC6687202; doi:10.1371/journal.pbio.3000408)
Supplement: S3 Table — (PDF) [file pbio.3000408.s013.pdf]

Accession numbers of reference sequences

AMT/RH
(B.floridae C3Z1J5) (B.floridae C3Y022) (B.floridae C3YB73) (B.floridae C3Z1G1) (B.floridae D7UQE4) (B.floridae D7UQE3) (B.floridae D7UQE2) (B.floridae D7UQE0) (B.floridae D7UQE1) (B.floridae D7UQD9) (B.floridae C3YB74) (B.floridae C3YB79) (C.gigas K1R302) (C.gigas K1QL97) (C.gigas K1QFY1) (C.gigas K1PJE2) (C.gigas K1RSL2) (C.intestinalis Q5VHU3) (C.intestinalis Q6XZ10) (C.intestinalis Q6XZ09) (C.intestinalis Q6XZ08) (C.intestinalis Q5VHU5) (C.intestinalis Q5VHU4) (T. adhaerens B3SC87) (T. adhaerens B3SC46) (T. adhaerens 196016156) (T. adhaerens 196016158) (T. adhaerens B3RRT6) (C.elegans spP54145) (C.elegans Q17663) (C.elegans Q9N2M5) (C.elegans Q20605) (C.elegans Q9N2M4) (C.elegans Q21565) (S.purpuratus W4Y0G6) (S.purpuratus W4YF70) (S.purpuratus W4Y3R7) (L.gigantea V4CRP5) (L.gigantea V3ZVE4) (L.gigantea V4AP67) (L.gigantea V3ZBD8) (L.gigantea 676492623) (L.gigantea 676434770) (C.teleta R7TS58) (C.teleta R7V3I9) (C.teleta R7U542) (C.teleta 443734385) (C.teleta 443691773) (C.teleta R7UG21) (H.sapiens Q02094) (H.sapiens Q9UBD6) (H.sapiens Q9H310) (M.leidyi ML10396a) (M.leidyi ML00673a) (M.leidyi ML018017a) (A.mellifera Q1L729) (A.mellifera Q38SD4) (D.sechellia B4HDZ9) (D.yakuba A0A0R1E419) (D.persimilis B4GLA6) (D.busckii A0A0M3QYI4) (D.melanogaster M9PEN8) (D.melanogaster Q9VFA9) (D.busckii A0A0M4E893) (D.pseudoobscura pseudoobscura Q4VUH9) (A.gambiae 118793733) (A.gambiae Q7Z1M1) (A. sinensis A0A084W5B9) (A.darling W5J3B1) (A.darling 568253901) (G.cydonium c.2546950) (A.queenslandica 761911958) (A.queenslandica 340386356) (G.pyriformis U3LYH9) (G.pyriformis U3LY22) (G.pyriformis U3LZ95) (H.cylindrosporum Q96UX9) (H.cylindrosporum Q96UY0) (H.cylindrosporum Q8NKD5) (S.pombe Q9C0V1) (S.pombe Q9US00) (S.cerevisiae P41948) (S.cerevisiae P40260) (S.cerevisiae P53390) (O.sativa Q8S230) (O.sativa Q8S233) (O.sativa Q84KJ7) (A.thaliana Q9M6N7) (O.sativa Q84KJ6) (O.sativa Q69T29) (O.sativa Q851M9) (O.sativa Q7XQ12) (O.sativa A0A0P0WCF8) (O.sativa Q6K9G1) (O.sativa Q6K9G3) (A.thaliana Q9ZPJ8) (A.thaliana Q9SQH9) (A.thaliana Q9LK16) (A.thaliana P54144) (A.thaliana Q9SVT8) (Archaea Q8TIE5 [Methanosarcina) (Archaea Q8TZ86 [Methanopyrus) (Archaea A0A075IBT0 [marine thaumarchaeote) (Archaea A0A075HAR1 [marine thaumarchaeote) (Archaea A0A075I7G1 [marine thaumarchaeote) (Archaea G4RK28 [Thermoproteus) (Archaea A0A075GR62 [marine thaumarchaeote) (Archaea A0A075GM15 [marine thaumarchaeote) (Archaea A0A075FUF3 [marine thaumarchaeote) (Archaea A0A075H4I2 [marine thaumarchaeote) (Archaea A0A075HUT8 [marine thaumarchaeote) (Archaea Q8TJ70 [Methanosarcina) (Archaea A0A075ICL8 [marine euryarchaeote) (Archaea A0A089ZF16 [Methanobacterium) (Bacteria 769129665 [Lachnospiraceae) (Bacteria 736086132 [Lachnospiraceae) (Bacteria E0RUI [Butyrivibrio) (Bacteria 769141800 [Lachnospiraceae) (Bacteria 769173412 [Clostridium) (Bacteria 291528577 [Eubacterium]) (Bacteria 737671611 [Lachnospiraceae) (Bacteria 736431606 [Butyrivibrio) (Bacteria 490181861 [Clostridium) (Bacteria 932916429 [Anaerostipes) (Bacteria 736105025 [Lachnospiraceae) (Bacteria 769254574 [Lachnospiraceae) (Bacteria 551041730 [Lachnospiraceae) (Bacteria Q07429 [Bacillus) (Bacteria O66515 [Aquifex) (Bacteria A0A0D0ITJ4 [Pseudomonas) (Bacteria P69681 [Escherichia) (I. pulchra MN101604 amt2/3b) (I. pulchra MN101601 amt-like) (I. pulchra MN101603 amt2/3a) (I. pulchra MN101605 amt2/3c) (I. pulchra MN101617 Rh) (I. pulchra MN101602 amt1/4b) (I. pulchra MN101600 amt1/4a) (M.stichopi MN101618 Rh) (M.stichopi MN101606 amt) (N.vectensis A7SSQ4 amt1/4a) (N.vectensis A7RH04 amt1/4b) (N.vectensis A7SQ16 amt2/3c) (N.vectensis A7S731 amt2/3a) (N.vectensis A7S3L2 amt2/3e) (N.vectensis A7RNC3 amt2/3b) (N.vectensis A7SGD4 amt2/3d) (N.vectensis MN101614 Rh1) (N.vectensis MN101615 Rh2) (N.vectensis MN101616 Rh3)

SLC
(S.mediterranea.slc26a-2|m.283_sm.slc26a-2| g.283 sm.slc26a-2:109-1878) (S.mediterranea.slc13a-4|m.264_sm.slc13a-4| g.264 sm.slc13a-4:3-1787) (S.mediterranea.slc1a-3|m.5_sm.slc1a-3| g.5 sm.slc1a-3:155-1555) (S.mediterranea.slc1a-1|m.1_sm.slc1a-1| g.1 sm.slc1a-1:238-567) (S.mediterranea.slc4a-1|m.16_Sm.slc4a-1| g.16 Sm.slc4a-1:107-3799) (S.mediterranea.slc4a-2|m.25_Sm.slc4a-2| g.25 Sm.slc4a-2:37-2943) (S.mediterranea.slc1a- 4|m.10_sm.slc1a-4| g.10 sm.slc1a-4:1-1443) (S.mediterranea.slc1a-5|m.12_sm.slc1a-5| g.12 sm.slc1a-5:202-1548) (S.mediterranea.slc1a-2|m.3_sm.slc1a-2| g.3 sm.slc1a-2:103-1890) (S.mediterranea.slc4a-6|m.55_Sm.slc4a-6| g.55 Sm.slc4a-6:17-3538) (S.mediterranea.slc4a-7|m.60_Sm.slc4a-7| g.60 Sm.slc4a-7:69-3083) (S.mediterranea.slc4a- 3|m.34_Sm.slc4a-3| g.34 Sm.slc4a-3:94-5439) (S.mediterranea.slc4a-4|m.46_Sm.slc4a-4| g.46 Sm.slc4a-4:296-2971) (S.mediterranea.slc4a-8|m.67_Sm.slc4a-8| g.67 Sm.slc4a-8:119-2866) (S.mediterranea.slc4a-5|m.52_Sm.slc4a-5| g.52 Sm.slc4a-5:50-2626) (S.mediterranea.slc4a-9|m.75_Sm.slc4a-9| g.75 Sm.slc4a-9:1-1545) (S.mediterranea.slc4a-10|m.78_Sm.slc4a-10| g.78 Sm.slc4a-10:3-509) (S.mediterranea.slc26a-7|m.313_sm.slc26a-7| g.313 sm.slc26a-7:240- 2279) (S.mediterranea.slc26a-10|m.334_sm.slc26a-10| g.334 sm.slc26a-10:284-1114) (S.mediterranea.slc26a- 6|m.308_sm.slc26a-6| g.308 sm.slc26a-6:145-2025) (S.mediterranea.slc26a-9|m.330_sm.slc26a-9| g.330 sm.slc26a- 9:1-1161) (S.mediterranea.slc26a-5|m.303_sm.slc26a-5| g.303 sm.slc26a-5:128-1189) (S.mediterranea.slc26a-4|m.297_sm.slc26a-4| g.297 sm.slc26a-4:279-1808) (S.mediterranea.slc26a-8|m.320_sm.slc26a-8| g.320 sm.slc26a- 8:55-2334) (S.mediterranea.slc26a-3|m.289_sm.slc26a-3| g.289 sm.slc26a-3:757-2277) (S.mediterranea.slc26a-1|m.280_sm.slc26a-1| g.280 sm.slc26a-1:28-2529) (S.mediterranea.slc26a-5|m.304_sm.slc26a-5| g.304 sm.slc26a-5:1237-2172) (S.mediterranea.slc26a-4|m.298_sm.slc26a-4| g.298 sm.slc26a-4:1690-2163) (S.mediterranea.slc26a-3|m.290_sm.slc26a-3| g.290 sm.slc26a-3:1-489) (S.mediterranea.slc13a-6|m.275_sm.slc13a-6| g.275 sm.slc13a-6:54-1754) (S.mediterranea.slc13a-5|m.267_sm.slc13a-5| g.267 sm.slc13a-5:88-1785) (S.mediterranea.slc13a-7|m.278_sm.slc13a-7| g.278 sm.slc13a-

7:41-1714) (S.mediterranea.slc13a-3|m.259_sm.slc13a-3| g.259 sm.slc13a-3:69-1811) (S.mediterranea.slc13a-2|m.255_sm.slc13a-2| g.255 sm.slc13a-2:15-287) (S.mediterranea.slc12a-2|m.218_sm.slc12a-2| g.218 sm.slc12a-2:30-3044) (S.mediterranea.slc12a-3|m.229_sm.slc12a-3| g.229 sm.slc12a-3:759-1625) (S.mediterranea.slc12a-4|m.234_sm.slc12a-4| g.234 sm.slc12a-4:327-2621) (S.mediterranea.slc12a-1|m.210_sm.slc12a-1| g.210 sm.slc12a-1:86-3280) (S.mediterranea.slc12a-5|m.244_sm.slc12a-5| g.244 sm.slc12a-5:187-2193) (S.mediterranea.slc12a-4|m.235_sm.slc12a-4| g.235 sm.slc12a-4:2647-3723) (S.mediterranea.slc9a-1|m.159_sm.slc9a-1| g.159 sm.slc9a-1:132-2207) (S.mediterranea.slc9a-4|m.174_sm.slc9a-4| g.174 sm.slc9a-4:112-2274) (S.mediterranea.slc9a-7|m.191_sm.slc9a-7| g.191 sm.slc9a-7:31-1950) (S.mediterranea.slc9a-8|m.196_sm.slc9a-8| g.196 sm.slc9a-8:3-1991) (S.mediterranea.slc9a-2|m.162_sm.slc9a-2| g.162 sm.slc9a-2:45-2078) (S.mediterranea.slc9a-5|m.180_sm.slc9a-5| g.180 sm.slc9a-5:94-2115) (S.mediterranea.slc9a-3|m.166_sm.slc9a-3|g.166 sm.slc9a-3:34-2712) (S.mediterranea.slc9a-9|m.199_sm.slc9a-9| g.199 sm.slc9a-9:889-2871) (S.mediterranea.slc8a-1|m.113_Sm.slc8a-1| g.113 Sm.slc8a-1:350-3103) (S.mediterranea.slc8a-2|m.124_Sm.slc8a-2|g.124 Sm.slc8a-2:82-2637) (S.mediterranea.slc8a-4|m.139_Sm.slc8a-4| g.139 Sm.slc8a-4:354-2546) (S.mediterranea.slc8a-5|m.149_Sm.slc8a-5| g.149 Sm.slc8a-5:68-2665) (S.mediterranea.slc8a-3|m.132_Sm.slc8a-3|g.132 Sm.slc8a-3:1114-2832) (S.mediterranea.slc8a-3|m.133_Sm.slc8a-3| g.133 Sm.slc8a-3:123-1193) (S.mediterranea.slc8a-4|m.140_Sm.slc8a-4| g.140 Sm.slc8a-4:1-450) (S.mediterranea.slc5a-2|m.86_Sm.slc5a-2| g.86 Sm.slc5a-2:62-1897) (S.mediterranea.slc5a-1|m.80_Sm.slc5a-1| g.80 Sm.slc5a-1:66-1862) (S.mediterranea.slc5a-3|m.97_Sm.slc5a-3| g.97 Sm.slc5a-3:53-1801) (S.mediterranea.slc5a-4|m.102_Sm.slc5a-4| g.102 Sm.slc5a-4:22-1860) (N.vectensis 173595) (N.vectensis 230013) (N.vectensis 1228981) (N.vectensis 16302) (N.vectensis XP_001636607.1) (N.vectensis 156223712) (N.vectensis 89958) (N.vectensis 31622) (N.vectensis XP_001634186.1) (N.vectensis 156221266) (N.vectensis 238013) (N.vectensis 104399) (N.vectensis A7SQZ6) (N.vectensis A7S331) (N.vectensis XM_001623843.1) (N.vectensis XM_001637857.1) (N.vectensis XP_001641754.1) (N.vectensis XM_001641374.1) (N.vectensis XM_001626153.1) (N.vectensis XP_001626203.1) (N.vectensis XM_001632684.1) (N.vectensis XP_001632734.1) (N.vectensis XM_001635065.1) (N.vectensis XP_001635115.1) (N.vectensis A7SNT1) (N.vectensis A7SHZ4) (N.vectensis A7S542) (N.vectensis A7SC64) (N.vectensis A7S539) (N.vectensis A7SX54) (N.vectensis A7SWE4) (N.vectensis A7S2I0) (N.vectensis XP_001638856.1) (N.vectensis 156225980) (N.vectensis XP_001627572.1) (N.vectensis 156214486) (N.vectensis XP_001637705.1) (N.vectensis 156224819) (A.mediterranea A0A1J0SZ44) (A.mediterranea A0A1J0SLW4) (A.mediterranea A0A1J0TAF6) (A.queenslandica I1FF21) (A.queenslandica I1FF19) (A.queenslandica I1FF22) (A.queenslandica I1FS59) (H.sapiens P43004) (H.sapiens P43003) (H.sapiens P48664) (H.sapiens O00341) (H.sapiens P43005) (H.sapiens P43007) (H.sapiens Q15758) (H.sapiens Q2Y0W8) (H.sapiens Q6U841) (H.sapiens Q9Y6M7) (H.sapiens Q9Y6M7) (H.sapiens Q9Y6M7) (H.sapiens Q9Y6M7) (H.sapiens Q9Y6R1) (H.sapiens Q9BY07) (H.sapiens Q96Q91) (H.sapiens P04920) (H.sapiens P48751) (H.sapiens P02730) (H.sapiens Q8NBS3) (H.sapiens P50443) (H.sapiens Q9H2B4) (H.sapiens O43511) (H.sapiens P40879) (H.sapiens P58743) (H.sapiens Q9BXS9) (H.sapiens Q7LBE3) (H.sapiens Q96RN1) (H.sapiens Q8TE54) (H.sapiens Q86WA9) (H.sapiens Q8NG04) (H.sapiens Q13183) (H.sapiens Q86YT5) (H.sapiens Q8WWT9) (H.sapiens Q9BZW2) (H.sapiens Q9UKG4) (H.sapiens P55011) (H.sapiens Q13621) (H.sapiens P55017) (H.sapiens Q9BXP2) (H.sapiens Q9UHW9) (H.sapiens Q9UP95) (H.sapiens Q9H2X9) (H.sapiens Q9Y666) (H.sapiens A0AV02) (H.sapiens Q9UBY0) (H.sapiens Q6AI14) (H.sapiens P19634) (H.sapiens P48764) (H.sapiens Q14940) (H.sapiens Q92581) (H.sapiens Q96T83) (H.sapiens Q8IVB4) (H.sapiens Q9Y2E8) (H.sapiens P32418) (H.sapiens P57103) (H.sapiens Q9UPR5) (H.sapiens P13866) (H.sapiens Q9NY91) (H.sapiens P31639) (H.sapiens Q2M3M2) (H.sapiens A0PJK1) (H.sapiens Q8WWX8) (H.sapiens P53794) (H.sapiens Q8N695) (H.sapiens Q1EHB4) (H.sapiens Q92911) (H.sapiens Q9Y289) (H.sapiens Q9GZV3) (I. pulchra MN101683 SLC12a) (I. pulchra MN101684 SLC12b) (I. pulchra MN101675 SLC9) (I. pulchra MN101674 SLC8) (I. pulchra MN101685 SLC13) (I. pulchra MN101678 SLC4c) (I. pulchra MN101676 SLC4a) (I. pulchra MN101677 SLC4b) (I. pulchra MN101681 SLC5a) (I. pulchra MN101682 SLC5b) (I. pulchra MN101679 SLC26a) (I. pulchra MN101680 SLC26b) (I. pulchra MN101672 SLC1b) (I. pulchra MN101671 SLC1a) (I. pulchra MN101673 SLC1c) (M.stichopi MN101691 SLC26b) (M.stichopi MN101690 SLC26a) (M.stichopi MN101696 SLC12c) (M.stichopi MN101695 SLC12b) (M.stichopi MN101694 SLC12a) (M.stichopi MN101697 SLC13a) (M.stichopi MN101698 SLC13b) (M.stichopi MN101699 SLC13c) (M.stichopi MN101700 SLC13d) (M.stichopi MN101688 SLC4b) (M.stichopi MN101689 SLC4c) (M.stichopi MN101687 SLC4a) (M.stichopi MN101692 SLC8) (M.stichopi MN101693 SLC9) (M.stichopi MN101701 SLC5a) (M.stichopi MN101702 SLC5b) (M.stichopi MN101686 SLC1)

AQUAPORINS
(A.thaliana P43286|PIP21) (A.thaliana P43287|PIP22) (A.thaliana P93004|PIP27) (A.thaliana P61837|PIP11) (A.thaliana Q08733|PIP13) (A.thaliana P30302|PIP23) (A.thaliana Q39196|PIP14) (A.thaliana Q06611|PIP12) (A.thaliana Q9FF53|PIP24) (A.thaliana Q9ZV07|PIP26) (A.thaliana Q9ZVX8|PIP28) (A.thaliana Q9SV31|PIP25) (A.thaliana Q8LAA6|PIP15) (X.tropicalis F6SCI3) (X.tropicalis Q5EBG0) (X.tropicalis F6RVN7) (X.tropicalis F6QPA9) (X.tropicalis (X.tropicalis F7CQ45) (X.tropicalis F7CFD3) (X.tropicalis F6QEC2) (X.tropicalis F6QNV6) (X.tropicalis F6Z564) (X.tropicalis A0A060PX33) (X.tropicalis Q28DL6) (X.tropicalis Q5FW23) (X.tropicalis A0A1B8YA67) (X.tropicalis A4IGW1) (X.tropicalis Q6DJ01) (X.tropicalis F6RVG6) (X.tropicalis F6YXD3) (X.tropicalis B0BM29) (X.tropicalis F6V0M7 (X.tropicalis A0A1B8XXZ7) (X.tropicalis F6S813) (X.tropicalis A0A1B8XXT0) (X.tropicalis F6QCC5) (X.tropicalis B4F717) (X.tropicalis F6Z1F2) (X.tropicalis F6Q4J3) (H.sapiens P29972) (H.sapiens P41181) (H.sapiens Q92482) (H.sapiens P55087) (H.sapiens P55064) (H.sapiens Q13520) (H.sapiens O14520) (H.sapiens O94778) (H.sapiens O43315) (H.sapiens Q96PS8) (H.sapiens Q8NBQ7) (H.sapiens Q8IXF9) (H.sapiens A6NM10) (C.gigas K1QMA6) (C.gigas K1QP58) (C.gigas K1QV92)

(C.gigas K1QSP9) (C.gigas K1PA25) (C.gigas K1QNC6) (C.gigas K1QC31) (C.gigas K1RM00) (C.gigas K1R4H0) (C.gigas K1QUZ8) (C.gigas K1RGW0) (C.gigas K1RAK5) (C.gigas K1QBQ1) (C.gigas K1QCC4) (C.gigas K1RA04) (D.melanogaster P23645) (D.melanogaster P23645) (D.melanogaster E1JH55) (D.melanogaster Q7KY01) (D.melanogaster M9PB86) (D.melanogaster A0A0B4KFZ1) (D.melanogaster A0A0C4DHF9) (D.melanogaster Q9W1M2) (D.melanogaster Q9W1M4) (D.melanogaster Q9W1M3) (D.melanogaster D1Z394) (D.melanogaster Q8MLR2) (D.melanogaster D3DMU9) (D.melanogaster F3YDC9) (D.melanogaster Q6NR72) (D.melanogaster A0A0B4KFN8) (D.melanogaster A0A0B4KET4) (D.melanogaster A0A0B4KFD6) (D.melanogaster A0A0B4LG55) (D.melanogaster A1Z8L8) (D.melanogaster Q95TS2) (D.melanogaster J7K3P9) (D.melanogaster H6V591) (D.melanogaster Q8T0N8) (D.melanogaster H5V8H0) (D.melanogaster H8F4Q9) (C.elegans Q19949) (C.elegans Q8IG23) (C.elegans Q18352) (C.elegans Q21473) (C.elegans Q9XW36) (C.elegans D5MCS1) (C.elegans H2FLH4) (C.elegans Q09369) (C.elegans G5EEK0) (C.elegans O46024) (C.elegans Q17571) (C.elegans Q7JMQ6) (C.elegans Q7JL16) (C.elegans Q7Z137) (C.elegans Q7Z138) (C.elegans Q18469) (C.elegans Q7YWQ1) (C.elegans A0A131MBS2) (C.elegans A0A061ADS9) (C.elegans A0A2K5AU16) (C.elegans A0A2K5AU20) (D.discoideum Q54WT8) (D.discoideum Q9U8P7) (D.discoideum Q54V53) (D.discoideum Q8SSP2) (D.discoideum Q54FQ9) (T.adhaerens B3RKX8) (T.adhaerens B3RR23) (A.queenslandica A0A1X7VPT7) (A.queenslandica A0A1X7UWA0) (A.queenslandica A0A1X7VQ25) (A.queenslandica A0A1X7UXK7) (A.queenslandica A0A1X7VNT9) (S.purpuratus W4ZC70) (S.purpuratus W4Y8N5) (S.purpuratus W4XM79) (S.purpuratus W4ZGE5) (S.purpuratus W4YZV4) (S.purpuratus W4XVQ3) (S.purpuratus W4YZV5) (S.purpuratus W4Y910) (S.purpuratus W4Z8B2) (S.purpuratus W4XXY7) (S.purpuratus W4YYT0) (S.purpuratus W4Z759) (S.purpuratus W4XQ97) (S.purpuratus W4XRA4) (S.purpuratus W4YD56) (N.vectensis A7S565) (N.vectensis A7S514) (N.vectensis A7S515) (N.vectensis A7T0G8) (N.vectensis A7RU64) (N.vectensis A7RU63) (N.vectensis A7S5L0) (N.vectensis A7T581) (N.vectensis A7T6P4) (N.vectensis A7SKN0) (M.stichopi. MN101662 AQf) (M.stichopi MN101660 AQd) (M.stichopi MN101659 AQc) (M.stichopi MN101661 AQe) (M.stichopi MN101657 AQa) (M.stichopi MN101658 AQb) (I. pulchra MN101651 AQb) (I. pulchra MN101655 AQf) (I. pulchra MN101650 AQa) (I. pulchra MN101652 AQc) (I. pulchra MN101654 AQe) (I. pulchra MN101653 AQd) (I. pulchra MN101656 AQg)

SLIPINS
(C.elegans Q27433GN) (C.elegans Q19958) (C.elegans Q21190) (C.elegans Q22165) (C.elegans Q19200) (C.elegans Q20657) (C.elegans G5ED76) (C.elegans H2FLJ1) (C.elegans Q9XWC6) (C.elegans H2L024) (D.melanogaster Q9VZA4) (D.melanogaster Q8MZ13) (D.melanogaster Q9VWL0) (D.melanogaster Q9W1F7) (H.sapiens Q9NP85) (H.sapiens P27105) (H.sapiens Q9UJZ1) (H.sapiens A0A024R882) (H.sapiens Q8TAV4) (H.sapiens Q9UBI4) (X.tropicalis Q6GLC6) (X.tropicalis Q6P362) (X.tropicalis F6VV69) (X.tropicalis F6WE98) (N.vectensis jgi|Nemve1|91851) (N.vectensis jgi|Nemve1|247670) (N.vectensis jgi|Nemve1|41366) (N.vectensis jgi|Nemve1|218685) (N.vectensis jgi|Nemve1|147236) (N.vectensis jgi|Nemve1|98373) (N.vectensis jgi|Nemve1|164373) (N.vectensis jgi|Nemve1|98301) (Bacteria A0E8T9 [Paramecium) (Bacteria Q5A411 [Candida) (Bacteria I7M6K2 [Tetrahymena) (A.queenslandica I1FPX0) (A.queenslandica I1FPW9) (A.queenslandica I1EJ07) (A.queenslandica I1FUT2) (A.queenslandica I1FXM9) (T.adhaerens B3SEH1) (T.adhaerens B3RKW0) (T.adhaerens B3RW09) (T.adhaerens B3RSS8) (T.adhaerens B3RKV9) (I. pulchra MN101663 Stomatin/podocin a) (I. pulchra MN101664 Stomatin/podocin b) (I. pulchra MN101665 Stomatin/podocin c) (M.stichopi MN101670 Stomatin/podocin e) (M.stichopi MN101669 Stomatin/podocin d) (M.stichopi MN101668 Stomatin/podocin c) (M.stichopi MN101667 Stomatin/podocin b) (M.stichopi MN101666 Stomatin/podocin a)

CD2AP
(H.sapiens Q9Y5K6) (D.melanogaster Q9Y154) (A.queenslandica XP_003386167.1) (N.vectensis XP_001630773.1) (H.sapiens P02549) (H.sapiens P11277) (D.melanogaster Q9Y154) (S.kowalevskii XP_006826005.1) (H.vulgaris XP_0021658) (T.adhaerens RDD45964.1) (I. pulchra MN101646) (M.stichopi MN101647)

NEPHRIN/KIRRE
(H.sapiens Q96J84) (H.sapiens Q8IZU9) (H.sapiens Q6UWL6) (H.sapiens O60500) (A.mellifera XP_026296514.1) (C.gigas EKC26159.1) (T.castaneum XP_008190700.1) (X.tropicalis F7EAD2) (X.tropicalis F7BA02) (D.melanogaster Q08180) (D.melanogaster Q9N9Y9) (D.melanogaster Q9V787) (D.melanogaster Q9V4Y0) (A.cerana PBC28977.1) (M.lignano PAA87588.1) (M.lignano PAA94659.1) (S.kowalevskii NP_001164704.1) (C.elegans B1Q236) (C.elegans Q9U3P2) (B.glabrata A0A075T6F5) (I. pulchra MN101641 Nephrin/Kirre2) (I. pulchra MN101640 Nephrin/Kirre1) (I. pulchra MN101642 Nephrin/Kirre3) (M.stichopi MN101644 Nephrin/Kirre2) (M.stichopi MN101645 Nephrin/Kirre3) (M.stichopi MN101643 Nephrin/Kirre1)

ZO1
(H.sapiens Q07157) (H.sapiens Q9UDY2) (H.sapiens Q2VPE5) (D.melanogaster Q94880) (T.adhaerens B3S7T9) (C.elegans Q8I103) (S.purpuratus XP_782687.2) (N.vectensis A7S398) (D.melanogaster P31007) (M.musculus Q62108) (GQ290472.1_Capsaspora) (GQ290473_Capsaspora) (I. pulchra MN101648) (M.stichopi MN101649)

CA
(N.vectensis A7T609) (N.vectensis A6QR78) (N.vectensis A7S2D8 CA a1) (N.vectensis A7SHT0) (N.vectensis A7S717) (N.vectensis A7SKA8 CA a3) (N.vectensis A7RRH8) (N.vectensis A7SHS9 CA a2) (N.vectensis

A7RR00) (N.vectensis A7S762) (H.sapiens P00918) (H.sapiens P00915) (H.sapiens Q8N1Q1) (H.sapiens P07451) (H.sapiens P43166) (H.sapiens P35218) (H.sapiens Q9Y2D0) (H.sapiens O43570) (H.sapiens Q9ULX7) (H.sapiens Q16790) (H.sapiens P35219) (H.sapiens P22748) (H.sapiens P23280) (H.sapiens O75493) (H.sapiens Q9NS85) (T.castaneum D6WET4) (T.castaneum A0A139WMN7) (T.castaneum D7EIC1) (T.castaneum D2A0V8) (T.castaneum D2A110) (T.castaneum A0A139WMC5) (T.castaneum D6W9D1) (T.castaneum D6W9D0) (T.castaneum D6W9D3) (A.queenslandica A6QR77) (A.queenslandica I1FTL5) (A.queenslandica A6QR75) (A.queenslandica I1FAY3) (A.queenslandica I1FAY2) (A.queenslandica I1FAY4) (A.queenslandica A6QR76) (A.queenslandica I1GDV3) (A.queenslandica I1G206) (T.adhaerens B3RXW0) (T.adhaerens B3RJD2) (T.adhaerens B3RVV0) (T.adhaerens B3RKE2) (T.adhaerens B3RKE1) (T.adhaerens B3RWG7) (T.adhaerens B3RKE3) (T.adhaerens B3RKE1) (T.adhaerens B3RKE0) (C.teleta R7T953) (C.teleta R7U445) (C.teleta R7UU94) (C.teleta R7V8T6) (C.teleta R7TR34) (C.teleta R7UAK2) (C.teleta R7TGJ8) (C.teleta R7V5W2) (C.teleta R7TB34) (C.teleta R7U704) (C.teleta R7U0D8) (C.teleta R7UH28) (S.purpuratus W4Y433) (S.purpuratus W4YCH9) (S.purpuratus W4YCI0) (S.purpuratus W4YNP4) (S.purpuratus W4Z2K3) (S.purpuratus W4XCN3) (S.purpuratus W4YB71) (S.purpuratus W4XBF4) (S.purpuratus W4Z9I4) (S.purpuratus Q0QBU7) (S.purpuratus W4Y9V4) (S.purpuratus W4XYY9) (S.purpuratus W4XL56) (S.purpuratus W4ZC38) (I. pulchra MN101628 CA a) (I. pulchra MN101630 CA c) (I. pulchra MN101629 CA b) (I. pulchra MN101631 CA d) (I. pulchra MN101633 CA x) (I. pulchra MN101632 CA h) (M.stichopi MN101634 CA a) (M.stichopi MN101635 CA b) (M.stichopi MN101637 CA d) (M.stichopi MN101636 CA c)

V-ATPASE B
(N.vectensis A7SRN1) (N.vectensis A7S0L1 V-atpase B) (T.adhaerens B3SAY3) (T.adhaerens B3RW75) (C.teleta R7TIR2) (C.teleta R7V2Q5) (A.queenslandica I1GEG2) (A.queenslandica I1FHW5) (H.sapiens P21281) (H.sapiens P15313) (H.sapiens P38606) (S.cerevisiae P16140) (S.cerevisiae P17255) (D.melanogaster P31409) (D.melanogaster Q27331) (D.melanogaster P48602) (C.gigas EKC36437.1) (S.kowalevskii NP_001171741.1) (A.californica XP_005091800.1) (D.discoideum Q76NU1) (D.discoideum P54647) (I. pulchra MN101619 V-atpase B) (M.stichopi MN101621 V-atpase B2) (M.stichopi MN101620 V-atpase B1)

NKA
(D.purpureum F1A2S2) (D.purpureum Q95024) (A.queenslandica XP_011404074.1) (C.elegans G5EFV6) (C.elegans P90735) (T.adhaerens B3RIH3) (N.vectensis A7S6M1 NKA b) (N.vectensis A7SII2 NKA a) (H.sapiens P50993) (H.sapiens P13637) (H.sapiens P05023) (H.sapiens Q13733) (C.teleta R7V8F1) (C.teleta R7VBZ9) (S.purpuratus W4ZCC9) (D.melanogaster P13607) (M.stichopi MN101625) (I. pulchra MN101626 NKA a) (I. pulchra MN101627 NKA b)

HCN
(H.sapiens O60741) (H.sapiens Q9UL51) (H.sapiens Q9P1Z3) (H.sapiens Q9Y3Q4) (B.dorsalis A0A034V7Z9) (B.dorsalis A0A034V8L7) (S.purpuratus NP_999729.1) (S.purpuratus NP_001028182.1) (C.intestinalis H6V963) (C.intestinalis H6V964) (D.melanogaster AAD42059.1) (N.vectensis A0A0S2KP17) (N.vectensis A0A0S2KP23) (A.queenslandica XP_003385808.1) (A.queenslandica XP_011410082.2) (T.adhaerens XP_002111266.1) (T.adhaerens XP_002116397.1) (I. pulchra MN101638) (M.stichopi MN101639)

Transcripts numbers from Xenacoelomorph transcriptomes

AMT/RH
(H.miamia 98027843) (H.miamia 98057167) (H.miamia 98018981) (H.miamia 98054821) (C.macropyga 395.1) (C.macropyga 24654.1) (C.macropyga 14995.1) (C.macropyga 16173.1) (C.macropyga 10395.1) (C.macropyga 4405.1) (C.macropyga 1268.1) (C.macropyga 474.1) (X.bocki 7955.1) (X.profunda 10079.1) (N.westbladi 55127.0) (N.westbladi 37503.0) (N.westbladi 48661.0) (N.westbladi 49524.0) (D.longitubus c31154) (D.longitubus c29820) (D.longitubus c34329) (D.gymnopharyngeus c4043) (C.submaculatum c14126) (C.submaculatum c14702) (C.submaculatum c18521)

AQUAPORINS
(X.bocki 30275.1) (X.bocki 11732.1) (X.bocki 16293.1) (X.bocki 2579.1) (X.bocki 3609.1) (X.bocki 6004.1) (X.profunda16190.1) (X.profunda 2887.1) (X.profunda 5191.1) (X.profunda 16480.1) (C.macropyga 11373.1) (C.macropyga 18775.1) (C.macropyga 14142.1) (C.macropyga 16171.1) (C.macropyga 11010.1) (C.macropyga 20222.1) (C.macropyga 16428.1) (C.macropyga 1448.1) (C.macropyga 17222.1) (C.macropyga 15603.1) (E.macrobursalium c7828) (C.submaculatum c13797) (C.submaculatum c17016) (C.submaculatum c15544) (D.gymnopharyngeus c13920) (D.gymnopharyngeus c11428) (D.longitubus c31442) (D.longitubus c33092) (D.longitubus c29236) (H.miamia 98039895) (H.miamia 98046060) (H.miamia 98013247)

SLIPINS
(X.bocki 20883.1) (X.bocki 12758.1) (X.bocki 1047.1) (Ascoparia sp. 9383.0) (Ascoparia sp. 17607.0) (Ascoparia sp. 20287.1) (Ascoparia sp. 28149.0) (Sterreria sp. c17109) (C.macropyga 229.1) (C.macropyga 14931.1) (C.macropyga 729.1) (C.macropyga 10774.1) (C.submaculatum c17900) (C.submaculatum c18531) (C.submaculatum c19891) (N.westbladi 47675.) (N.westbladi 52579.0) (N.westbladi 25494.0)

(D.gymnopharyngeus c2158) (D.gymnopharyngeus c13414) (D.longitubus c30579) (D.longitubus c33065) (D.longitubus c36799) (E.macrobursalium c5714) (E.macrobursalium c6060) (E.macrobursalium c8407) (H.miamia 98000817) (H.miamia 98001079) (H.miamia 98003185) (H.miamia 98003968) (H.miamia 98009885) (H.miamia 98030377) (H.miamia 98031305) (H.miamia 98032202)

CD2AP
(X.bocki 1192.1) (N.westbladi 47339.0) (N.westbladi 51312.0) (D.gymnopharyngeus c14992)

NEPHRIN/KIRRE
(H.miamia 9805464) (H.miamia 98022051) (C.macropyga 2490.1) (C.macropyga 2933.1) (C.macropyga 5188.1) (X.bocki 5576.1) (X.bocki 12500.1) (X.profunda c18867) (X.profunda 14967.1) (X.profunda 13792.1) (N.westbladi 48390.0) (N.westbladi 51630.0) (D.longitubus c36603) (D.longitubus c38278) (D.longitubus c37893) (D.gymnopharyngeus c10380) (D.gymnopharyngeus c10580) (C.submaculatum c17370) (C.submaculatum c15556) (C.submaculatum c17370) (C.submaculatum c17306) (E.macrobursalium c4372)

ZO1
(H.miamia 98057262) (C.macropyga 5972.1) (X.bocki 3789.1) (N.westbladi 51947.0) (D.longitubus c38032) (D.gymnopharyngeus c12822) (D.gymnopharyngeus c13251) (C.submaculatum c19373)

CA
(Ascoparia sp. 5478.0) (Ascoparia sp. 18043.0) (Ascoparia sp. 25649.0) (Ascoparia sp. 2777.0) (C.submaculatum c1687) (C.submaculatum c13257) (C.submaculatum c5059) (C.submaculatum c13664) (D.gymnopharyngeus c13170) (D.gymnopharyngeus c10609) (D.gymnopharyngeus c11439) (D.gymnopharyngeus c13170) (C.macropyga 16271.1) (C.macropyga 3418.1) (C.macropyga 19581.1) (C.macropyga 21507.1) (C.macropyga 18799.1) (C.macropyga 18136.1) (C.macropyga 13579.1) (C.macropyga 7849.1) (C.macropyga 1531.1) (C.macropyga 9483.1) (D.longitubus c35395) (D.longitubus c14121) (D.longitubus c28511) (D.longitubus c28413) (D.longitubus c33567) (E.macrobursalium c491) (E.macrobursalium c1233) (E.macrobursalium c7687) (E.macrobursalium c4985) (E.macrobursalium c122) (E.macrobursalium c6561) (E.macrobursalium c3860) (N.westbladi 29328.0) (N.westbladi 45461.0) (N.westbladi 39569.0) (N.westbladi 25845.0) (N.westbladi 45772.0) (N.westbladi 12921.0) (N.westbladi 50761.0) (H.miamia 98030173) (H.miamia 98042177) (H.miamia 98029235) (H.miamia 98046701) (H.miamia 98057584) (H.miamia 98008272) (H.miamia 98044099) (Sterreria sp. c22223) (X.profunda 20645.1) (X.profunda 8011.1) (X.profunda c9812) (X.profunda 22298.1) (X.profunda 10281.1) (X.profunda 19114.2) (X.bocki 3894.1) (X.bocki 4398.1) (X.bocki 1891.1) (X.bocki 13708.1) (X.bocki 25911.1) (X.bocki 46303.1) (X.bocki 29622.1) (X.bocki 29916.1) (M.stichopi 16170.1) (M.stichopi 9713.1) (M.stichopi 27008.1) (I. pulchra 7996.1) (I. pulchra 12647.1) (I. pulchra 23555.1) (I. pulchra 2042.1)

V-ATPASE
(X.bocki 1513.1) (X.profunda 15609) (N.westbladi 51758) (E.macrobursalium c7821) (C.macropyga 487.1) (D.longitubus c35334) (D.longitubus c35800) (D.longitubus c28900) (C.submaculatum c21601)

NKA
(H.miamia 98034285) (D.longitubus c37851) (C.submaculatum c18356) (C.macropyga 93.1) (X.bocki 812.1) (X.profunda 6567.1) (N.westbladi 52442.0)
