# Supplementary material for: Active mode of excretion across digestive tissues predates the origin of excretory organs
Source: PLoS Biol. 2019 Jul 29;17(7):e3000408. doi: 10.1371/journal.pbio.3000408 (PMC6687202; doi:10.1371/journal.pbio.3000408)
Supplement: S4 Table — QPCR, quantitative PCR. (PDF) [file pbio.3000408.s014.pdf]

I. pulchra

GENE	average fold change	replica 1	replica 2	replica 3	min	max	negative error	positive error
**rhesus**	2,217183893	1,7168389	2,56415513	2,37055765	1,7168389	2,56415513	0,500344997	0,346971237
**NKA a**	2,596248698	2,17808859	2,6920789	2,9185786	2,17808859	2,9185786	0,418160107	0,322329902
**NKA b**	1,693353664	1,59134502	1,59429737	1,8944186	1,59134502	1,8944186	0,102008643	0,201064933
**v-ATPase B**	0,494488592	0,50926761	0,44218674	0,53201143	0,44218674	0,53201143	0,052301849	0,037522836
**HCN**	1,254995092	1,1888023	1,02014386	1,55603912	1,02014386	1,55603912	0,234851234	0,301044031
**amt1/4 a**	2,155580868	2,27056	2,15934753	2,03683508	2,03683508	2,27056	0,118745787	0,114979129
**amt-like**	1,600769598	1,64926114	1,521413	1,63163465	1,521413	1,64926114	0,079356595	0,048491541
**amt1/4 b**	2,691183113	2,94608692	2,57428365	2,55317876	2,55317876	2,94608692	0,138004349	0,25490381
**amt2/3 a**	1,860833749	1,76944979	1,32323389	2,48981757	1,32323389	2,48981757	0,537599859	0,628983821
**amt2/3 b**	2,072839483	2,45194857	1,83274149	1,93382839	1,83274149	2,45194857	0,240097992	0,379109085
**amt2/3 c**	1,314142054	1,24229458	1,15235077	1,54778081	1,15235077	1,54778081	0,161791289	0,233638758
**aquaporin a**	2,577641717	2,85374828	2,75857472	2,12060215	2,12060215	2,85374828	0,457039564	0,276106565
**aquaporin b**	2,793732272	2,78000575	3,45075469	2,15043638	2,15043638	3,45075469	0,643295893	0,657022417
**aquaporin c**	2,70322904	2,88856343	2,80330324	2,41782045	2,41782045	2,88856343	0,285408591	0,185334389
**aquaporin d**	1,776819335	1,54068	1,98835987	1,80141813	1,54068	1,98835987	0,236139336	0,211540536
**aquaporin e**	2,077006893	2,01976667	2,50102388	1,71023012	1,71023012	2,50102388	0,366776771	0,424016989
**aquaporin f**	1,318446722	1,06018873	1,45500979	1,44014164	1,06018873	1,45500979	0,25825799	0,136563071
**aquaporin g**	1,709907854	1,15172762	1,85739378	2,12060215	1,15172762	2,12060215	0,558180229	0,4106943
**ca a**	1,81344051	1,85094045	1,8379878	1,75139329	1,75139329	1,85094045	0,062047223	0,037499938
**ca b**	0,44152293	0,53183025	0,38334267	0,40939586	0,38334267	0,53183025	0,058180255	0,090307325
**ca c**	2,101369012	2,34990803	1,99340395	1,96079506	1,96079506	2,34990803	0,140573957	0,248539015
**ca d**	1,903583872	2,30433541	1,49964688	1,90676933	1,49964688	2,30433541	0,403936991	0,400751534
**ca e**	1,202587419	1,13455072	1,23432496	1,23888658	1,13455072	1,23888658	0,068036702	0,036299156
**ca f**	1,658881375	1,58664991	1,94214714	1,44784707	1,44784707	1,94214714	0,211034303	0,28326577
**ca g**	1,874941708	2,42800103	1,3377446	1,85907949	1,3377446	2,42800103	0,537197105	0,553059325
**ca h**	2,264203899	2,16749309	2,32859122	2,29652739	2,16749309	2,32859122	0,096710812	0,064387323
**ca x**	1,883258685	1,58149009	1,68487764	1,91528729	1,58149009	1,91528729	0,301768597	0,032028609

N. vectensis

GENE	replica 1	replica 2	replica 3	replica 4	replica 5	average fold change	min	max	negative error	positive error
**rhesus 1**	0,66256924	0,58543965	0,5453938	0,2487322	0,42097777	0,492622531	0,2487322	0,66256924	0,243890335	0,16994671
**rhesus 2**	0,72148271	0,37254871	0,57360891	0,18700431	0,3513349	0,441195908	0,18700431	0,72148271	0,254191602	0,280286803
**rhesus 3**	1,16015502	0,99525448	0,67506186	0,60440554	1,29971411	0,946918203	0,60440554	1,29971411	0,342512661	0,352795906
**nka a**	1,40156068	1,12475528	1,2076442	0,90980731	0,94960978	1,118675449	0,90980731	1,40156068	0,208868139	0,282885229
**nka b**	0,8768764	0,9891905	1,16772962	1,90742149	0,67601966	1,123447537	0,67601966	1,90742149	0,447427872	0,783973957
**amt2/3 a**	1,00285841	0,49106638	0,44097256	0,59328951	0,42250718	0,590138809	0,42250718	1,00285841	0,167631628	0,412719604
**amt2/3 b**	1,30287673	1,06698811	0,57725253	0,52395472	1,27166109	0,948546639	0,52395472	1,30287673	0,424591915	0,354330096
**amt2/3 e**	1,64275158	1,10108814	1,47424144	2,81035319	3,15621983	2,036930837	1,10108814	3,15621983	0,935842699	1,11928899
**amt1/4 b**	2,14755411	3,61492989	2,33638672	1,6762092		2,443769981	1,6762092	3,61492989	0,767560779	1,171159911
**amt2/3 c**	1,15684685	1,6376369		0,5248662		1,106449982	0,5248662	1,6376369	0,581583786	0,531186919
**amt1/4 a**	1,41776293	1,01899733	1,21231911		0,70347258	1,088137987	0,70347258	1,41776293	0,384665408	0,329624941
**amt2/3 d**	0,88755083	0,7055751	1,01447442		0,51000849	0,77940221	0,51000849	1,01447442	0,269393718	0,235072214
**V ATPASE B**	1,30911533	1,51601338	2,00097628	2,94070826		1,941703313	1,30911533	2,94070826	0,632587983	0,999004949
**CA 2**	1,83671485	0,88322688	0,61075626	1,07265708		1,100838767	0,61075626	1,83671485	0,490082508	0,735876079
**CA 3**	1,90235733	0,8797604	2,32532928	0,97401599	1,19622438	1,455537476	0,8797604	2,32532928	0,575777081	0,869791801
**CA 1**	0,65628293	0,83646141	0,58710676	0,51367514	0,38731996	0,596169239	0,38731996	0,83646141	0,208849278	0,240292173
