# Supplementary material for: Active mode of excretion across digestive tissues predates the origin of excretory organs
Source: PLoS Biol. 2019 Jul 29;17(7):e3000408. doi: 10.1371/journal.pbio.3000408 (PMC6687202; doi:10.1371/journal.pbio.3000408)
Supplement: S5 Table — QPCR, quantitative PCR. (PDF) [file pbio.3000408.s015.pdf]

I.pulchra

GENE	QPCR PRIMER F	QPCR PRIMER R
AqA	GCTATTCTCATCCACCTGCTCG	GCTTGGCTCTTGAAATCTTCTGG
AqB	GATAACACGCCTCAATGCCTTTC	TCACATCGTAGTTGTCTCCTCCTTC
AqC	CGAGCGAAGCAGATGACAACTTG	GCAGGCAGATAGAGGAGACCTTTC
AqD	TGGCACAACCACTGGGTCTC	TTCTCGGCGTCTTCGGGAGTAG
AqE	TCAACCCCGACCTGGACAAC	CGACGAAGTAGAGGATTCCC
AqF	GCAACATCAACCCAAGCGTC	GAACAACCAGCCAGAGAACGAAC
AqG	TGGGACTGGGACCACTCGTTAC	CGAATGTCACCAATGAGATGCTCC
Rhesus	GAAGTTGTTCAGTGGGGAGAAGG	CTTGATGAGTGACATTGCCGTG
V-ATPase B	AGCCCATCAACCCCTACTCC	TCCTCCTTGTCCAGACTCTTG
NKA a	TGGTCGGAGTTTCATCAGAGACAC	CGAAGCAGAGTATGGAGCCTATCC
NKA b	ATCGCCAGAGAAATCACCCAC	CAATGAGAAAGACCACAGCCTCG
CA a	ACTATTCCCACCAATCGCCC	TGACGAGTTCTCCGTGTGTAACG
CA b	AGAGTGGCAGACCCCTGTTGAC	GGCGAAGTCGTCGTTGTTCTC
CA c	ATGACGAGCTTGTTCTGCG	TCGTAACACAGCGGGTTGTCTG
CA d	CAACTTTGGTGCCTTGACGC	TGAGATTCGGAGATGTAGAGGGG
CA x	TTCTGTCGGAGAGGGCTTGTGTTG	TACATCGGGTCGCTCACCATTC
CA e	ATCAGTTCCTCGCCCACTACTTC	TTCCTCCAGCAGCATCTTGC
CA f	AGTTACGAATCGGCGTTGGG	TCCACCTTCTTCTGCTGGTCAC
CA g	GGATAGAATGCTCTCACCCTACTGC	TGGCTGCGAAAGAACTAAGGC
CA h	CGCTTCTTCATCTTCAAGTGCC	TTGCTCATCTCCACCAACCG
HCN	TCCTGGACGAGATGAACGACTG	TACCACTCCGCTCACGAAAAGG
amt1/4 a	TGGCACTGGGAAGAAGACTGGAAC	GGAGAGGATGGTGTTGACGATG
amt-like	TGCTCATCACACAACACCGC	CCCGTCGTTCAATAGTTCCTCG
amt1/4 b	GACACCAGGGATTACGCATTTG	GGCAACCGAGTAGATGAGATACGA
amt2/3 a	GCTGGTGGCAAATGGAAACTG	CGAGCACTCCGTTGATGATGTAG
amt2/3 b	CAAAGATGACCACCAACACTGAAG	GTGATGACAGCGAGCGAGTTATC
amt2/3 c	TGCCCTCGCAACATTCATACTC	AACGCTGTGCTCCATCAAGTCC
SLC1A	TGAAGCAGTCAGGATGGGAGTC	GTGAGGTAGAGGAGGAGGTCGC
SLC1B	GCAGCATCGCCGTCTGTTATTAC	TTTCCCATCACAACCCTGGAC
SLC1C	AGCACGAGGGACCGATGTATC	GGACCTTTCCGAAGACGACG
SLC5A	GCATTCGTCACTCACTCACCTTTAC	CCATCGCTGTTCCTCCTGC
SLC5B	ATGCTGGCGGTCAGTGTGG	AGGACGGCGGTGGAGTAGAC
SLC13	CGTGAGGTTCAAGAAGGGGC	GAGAAGGAGGGCGTTTGGG
SLC4A	AGATGGGGGAGCGAGGACAG	TGGAAGGAGAGGGAGGAGACG
SLC4B	TCCAATGCGTCGGCAAAAG	TCCACGAAACATCTCAGCAGC
SLC4C	GCTTCAACTACAACCTCCACAACAC	GGCGAATCAACTGGGACGG
SLC8	TTCACGGCAACCACTCTCTCC	TCCCAATGTGTAGACGAGCAGC
SLC9	ATGGAGTGTTAGACCGAGAAATGG	ACGATGAAGGGCAGGGCGAC
SLC12A	TCAGAGGGAGGCAGGGACTC	GATGTTGAGCAGGCACCGTG
SLC12B	ACATCGGAGATTCATCCTCGC	CGGGTGTTGCCATTGTTGAG
SLC26A	TCCCAACACCGAAAGATGCTC	ACTGGAGATAGCGATGCCAATG
SLC26B	TCGCCTTGACGGAGACGAG	AGACAGCACGACCTCAGCCC
UBIQ	ACCCTCACTGGAAAAACCATCAC	TGTAATCAGACAGCGTTCGGC
18S	TGAATCTGCCTGCTGATGAACC	GCTGATGTCACAACCAACCCAG

N.vectensis

**GENE**	**QPCR PRIMER F**	**QPCR PRIMER R**
Rhesus 1	GGTCTCCTTCTTATCCTGTTCGTTG	CCGCTAAAACCATACTTCTTGAGG
Rhesus 2	CGTTCTTACGAAAGCACGCCTAC	AAACTTGTCATTCCCGCCCTCG
Rhesus 3	TCTTCTTCGCTTTCCCTTTCCTAC	GAATACTCCAGAGCAGATGATGGC
V-ATPase B	CAAAACAGTCTCTGGTGTCAATGG	CTTGGCGTCAATCCCTGATG
NKA a	TGCGAGATTCCTTTCAACTCTACC	TGTTCCTTGCCATTCACGAGG
NKA b	TCTTCTCAACCAACGCTGTGG	CAATGGCAATGGGAGTCTTTCC
CA 1	TGGATTGTGTGTGCTTGGTGTC	TATTTCACTGAGGGGAGGCGTC
CA 2	AAACTGGTTCCTGGCTTGTCATC	GACGAGTATTGGGTTCTTGAGCAC
CA 3	CCTCAAGAACAATGGGCACG	TCCGTCTATCAGATGCTCCGAG
amt2/3 a	TGTCTTCGTGACGGGAATCTTG	TGGCGATACAACCTACTGGGTC
amt2/3 b	ATCGGGATTTGGACTGCTGG	ATGCGTTACTGCTCGGGCTATCTC
amt2/3 c	CGTCTTTGGGGCTGGAACATTC	ACCGTGCTTAGGGACATCAAGG
amt1/4 a	TAGACCAGTGTAGCCACCACCATC	AGAGTAGTGCGAGCAAAAAGAACC
amt2/3 d	TGAAGAGGGCGAGGACAAATC	TCGGTGACTCCAAAAGTGCTCC
amt1/4 b	CTCGTGCCTTCTGACTGGATTC	ATGAACAATACCGCTGCCCG
amt2/3 e	CGGAATGGTCTCCAGTAAGAACG	CCTCGGCATCGGTGAAAAAG
ATPsynthase	TGCTGGGAAAGTTCTGGACCAATG	ACACCCTCCTTGACGGTAACATTC
EF1b	TGCTGCA TCAGAACAGAAACCTGC	TAAGCCTTCAAGCGTTCTTGCCTG
