# Supplementary material for: Active mode of excretion across digestive tissues predates the origin of excretory organs
Source: PLoS Biol. 2019 Jul 29;17(7):e3000408. doi: 10.1371/journal.pbio.3000408 (PMC6687202; doi:10.1371/journal.pbio.3000408)
Supplement: S6 Table — (PDF) [file pbio.3000408.s016.pdf]

I. pulchra
pmol NH4 per animal per hour

	Control	DMSO*	Azetazolamide	Concanamycin C *	Quabain	Colchicine	after_2h_Control	after_2h_50µM_NH4	after_2h_100µM_NH4	after_2h_200µM_NH4	after_2h_500µM_NH4
	42,62742521	62,33342696	31,4531089	27,01785193	14,70956306	12,6353423	26,0191263	26,0191263	40,56200919	54,53441086	78,95135429
	49,62515341	72,56609707	23,20801818	27,01785193	13,63306143	10,8536109	34,40401354	47,95069188	51,87748694	78,899369	69,33049955
	32,48673637	56,60929704	47,95069188	44,13147428	15,00184555	24,6844436	44,13147428	52,10043147	48,95418782	72,61512478	69,33049955
	44,13147428	66,83141339	61,50836799	56,60929704	24,68444357	22,718356	48,95418782	39,23799171	47,73407262	55,2929522	70,64345419
	52,10043147	48,95418782	46,19561677	46,19561677	26,47819255	23,1575641	43,59249133	41,20853741	47,73407262	55,2929522	74,19119324
	43,59249133	48,95418782	46,19561677	51,87748694	26,47819255	24,7622786	34,6161893				
	48,95418782	41,20853741		45,4514832			34,6161893				
	41,13605215	37,36167527		47,73407262			37,36167527				
	48,95418782						39,23799171				
	45,25537608										
	42,32261055										
	43,27804462										
	41,20853741										
	43,27804462										
	41,20853741										
Average	44,01061937	54,35235285	42,75190341	43,25439184	20,16421645	19,8019326	38,10370432	41,30335575	47,37236584	63,32696181	72,48940017
t-test		0,052230575	0,83165359	0,077211158	5,20263E-05	4,747E-05	0,039419879	0,545957446	0,008449637	0,005072575	7,26935E-08

Control	after_7days_1mM_NH4
23,71588874	259,6623324
51,42606959	270,8004128
30,69639541	334,0805279
25,84572563	270,8004128
51,42606959	282,4162554
28,16683535	307,1640794

N. vectensis
µmol NH4 per animal per hour

	Control	DMSO*	Azetazolamide	Concanamycin C*	Quabaine	Colchicine	after_2h_Control	after_2h_50µM_NH4	after_2h_100µM_NH4	after_2h_200µM_NH4	after_2h_500µM_NH4
	1,025711758	1,097886282	0,895284951	1,097886282	1,097886282	1,25782843	0,843166231	1,30135867	0,730611563	2,137012551	4,039038963
	1,097886282	1,257828435	1,441071263	1,097886282	1,441071263	0,95828195	0,744836051	1,30135867	0,828719668	2,137012551	3,845896683
	0,895284951	1,025711758	0,958281953	0,958281953	1,097886282	1,34633592	0,644118001	0,778120924	1,881189488	2,598688211	1,76495759
	1,175139389	1,346335921	1,542472687	1,025711758	1,542472687	1,02571176	0,730611563	0,882608688	1,78408251	2,729195244	2,001959922
	1,149594015	1,384596622	1,774305093	1,384596622	1,384596622	1,38459662	1,443259768			0,940001942	2,874573407
	1,080483741	1,384596622	1,667638993	1,223124743	1,384596622	1,38459662	1,231095327			1,461008078	2,874573407
	1,129761765	0,984755602	1,316054456	1,043560279	0,929264572	0,9847556					
	1,186498797	0,876900465	1,316054456	0,984755602	0,876900465	1,04356028					
	1,066227441										
	1,066227441										
	1,298103409										
	1,231095327										
	1,105876476										
	1,105876476										
Average	1,115269091	1,169826463	1,363895481	1,10197544	1,219334349	1,1732084	0,93951449	1,065861738	1,306150807	2,000486429	2,900166662
t-test		0,484990173	0,061284884	0,79864075	0,29095443	0,76278546	0,241984791	0,526863466	0,330013078	0,010633659	0,00248882

Control	after_7days_1mM_NH4
1,032285075	14,6402541
1,332124272	14,6402541
1,332124272	13,2205821
0,932183929	13,9123212
1,202947584	14,6402541
1,265889282	14,6402541
